# Evaluation of mental disorder with prioritization of its type by utilizing the bipolar complex fuzzy decision-making approach based on Schweizer-Sklar prioritized aggregation operators

**DOI:** 10.7717/peerj-cs.1434

**Published:** 2023-11-07

**Authors:** Tahir Mahmood, Ubaid ur Rehman, Xindong Peng, Zeeshan Ali

**Affiliations:** 1 Department of Mathematics and Statistics, International Islamic University, Islamabad, Pakistan; 2 School of Information Engineering, Shaoguan University, Shaoguan, China

**Keywords:** Artificial Intelligence, Decision making, Bipolar disorder, Bipolar complex fuzzy sets, Schweizer-Sklar prioritized aggregation operators

## Abstract

A clinically important loss in a person’s understanding, emotive power, or conduct is a symptom of a mental disorder. It generally occurs for genetic, psychological, and/or cognitive reasons and is accompanied by discomfort or limitationin significant functional areas. It can be handled using techniques similar to those used to treat chronic conditions (i.e., precautions, examination, medication, and recovery). Mental diseases take a variety of forms. Mental disorder is also identified as mental illness. The latter is a more usual phrase that incorporates psychological problems, psychosocial disorders, and (other) states of mind linked to considerable discomfort, operational limitations, or danger of loss of sanity. To rank the most prevalent types of mental disorders is a multi-attribute decision-making issue and thus this article aims to analyze the artificial intelligence-based evaluation of mental disorders and rank the most prevalent types of mental disorders. For this purpose, here we invent certain aggregation operators under the environment of the bipolar complex fuzzy set such as bipolar complex fuzzy Schweizer-Sklar prioritized weighted averaging, bipolar complex fuzzy Schweizer-Sklar prioritized ordered weighted averaging, bipolar complex fuzzy Schweizer-Sklar prioritized weighted geometric, bipolar complex fuzzy Schweizer-Sklar prioritized ordered weighted geometric operators. After that, we devise a procedure of decision-making for bipolar complex fuzzy information by employing the introduced operators and then take artificial data in the model of bipolar complex fuzzy set to rank the most prevalent types of mental disorders. Additionally, this article contains a comparative study of the introduced work with a few current works for exhibiting the priority and superiority of the introduced work.

## Introduction

Artificial intelligence is currently playing a vital role in every field of real and scientific life. It is useful in management sciences, social sciences, information sciences, and in medical sciences. Further, symptoms including different emotions, thoughts, or attitudes are identified as mental disorders. Distress and struggles dealing with routine tasks at work, family, or social environments can be indications of mental diseases. Emotional responses, thoughts, interaction, understanding, tolerance, faith, and consciousness rely on excellent mental health. Relationships, individual and emotional well-being, and giving back to the community or citizens all depend on good mental health. A key element of physical well-being is a mental state. Physical health both influences and is affected by it. People who suffer from a mental disorder are repeatedly hesitant to examine it. There can be a social stigma regarding a mental health disorder; however, like diabetes or cardiac disease, it is a chronic condition. Medication is a common treatment for mental health problems. Our knowledge of how the human brain functions is always growing, and there are therapies available to assist patients in controlling and managing mental health disorders. No matter one’s age, sexuality, region, wealth, social background, nationality, culture, belief or spirituality, sexual identity, genetic factors, or other characteristics of ethnic traditions, anyone can be impacted by a mental disorder. Although mental disorders can strike anyone at any age, three-quarters of all cases began before the age of 24. Various types of mental disorders occur. Some are minor and only marginally affect daily living, such as some fears (abnormal fears). Certain mental problems are so serious that a patient may require inpatient care. The best approaches to treating a health problem rely on it and how intense it is, just as with other ailments.

In countless genuine life disputes, noticed values of information are frequently confusing and doubtful due to partial and/or non-accessible information. To resolve the vagueness in information, ambiguity structuring has a critical role in making a model of the DM approach for humans when the information is partial or vague. A fuzzy set (FS) is a mathematical set containing the property that an object or element can be placed in a set, not in a set, or partially placed in a set introduced by Zadeh (1965). The FS is a critical technique to catch the inaccuracy and vagueness in numerous issues. It is interpreted by the truth degree between 0 and 1. The bipolar fuzzy set (BFS) investigated by Zhang (1994) contains the positive truth degree and negative truth degree and is an extension of FS. The range of positive truth degree $ \left[ 0,1 \right] $ and the range of the negative truth degree is $ \left[ -1,0 \right] $. The BFS can interpret the vagueness data from two different aspects that are positive and negative, therefore preserving the reliability of DM information.

In numerous genuine-world issues, there is a requirement for extra fuzzy information (2nd dimension) to tackle the doubtful and confusing data. In this sense, the complex fuzzy set (CFS) was deduced by Ramot et al. (2002). It is an amazing technique to interpret the 2nd dimension (extra fuzzy information). It is interpreted by the truth degree placed in the unit disc of the complex plane and models the information in the polar form. Additionally, Tamir, Jin & Kandel (2011) deduced a new interpretation of a CFS and described the information in the cartesian form. It is interpreted by the truth degree placed in the unit square of a complex plane. There are countless situations where the above-mentioned theories are not applicable, for example, in a situation where a person wants to consider the positive and negative views along with extra fuzzy information. In this sort of situation, the bipolar complex fuzzy set (BCFS) deduced by Mahmood & Rehman (2022) is a wonderful mathematical tool to handle the positive and negative views along with extra fuzzy information. It is interpreted by the positive truth degree and negative truth degree and is the extension of all the above-mentioned theories. The range of positive truth degree $ \left[ 0,1 \right] +\iota \left[ 0,1 \right] $ and the range of the negative truth degree is $ \left[ -1,0 \right] +\iota \left[ -1,0 \right] $.

### Literature Review

Roth (1955) interpreted the history of mental disorders. Harris & Barraclough (1998) investigated the mortality of mental disorders. Similarly, various other scholars have investigated mental disorders. For instance, Waern et al. (2002) studied mental disorders in elderly suicides, Verhaak et al. (2005) investigated chronic disease and mental disorder, Monahan & Steadman (1983) described mental disorder and crime, Morse (2011) investigated criminal law and mental disorder, Robbins, Monahan & Silver (2003) studied gender, violence, and mental disorder, Eaton, Muntaner & Sapag (1999) studied mental disorder and socioeconomic stratification. Silvana, Akbar & Audina (2018) classified the features of the mental disorder by utilizing fuzzy information. Liu et al. (2021) have diagnosed mental disorder in children by utilizing the fuzzy mathematical model. Chaudhuri, Chandrika & Kumari (2016) investigated mental health by employing neuro-fuzzy methods. van Draanen et al. (2022) investigated mental disorders and opioid overdose. The mental disorders due to COVID-19 and other epidemics were investigated by Leung et al. (2022). Denche-Zamorano et al. (2022) studied the enhancement of the risk of mental disorders in youth because of the lack of physical activities. Merikangas, Nakamura & Kessler (2022) investigated the epidemiology of mental disorders. Sumathi & Poorna (2017) predicted mental health problems through fuzzy clustering. The ECG-based mental depression evaluation through fuzzy computing was interpreted by Chiang (2015). Wang et al. (2017) investigated fuzzy maps on mental disorders. Kai, Vijayashree & Jayashree (2017) studied a diagnosis framework relying on fuzzy logic for mental disorders.

Zhang, Pandurangi & Peace (2007) studied major depressive and bipolar disorder. Han et al. (2018) investigated bipolar fuzzy cognition and bipolar disorder. Jun & Kavikumar (2011) studied finite state machines in bipolar fuzzy (BF) information. Bloch (2012) introduced a mathematical morphology under BFS. Wei et al. (2018), Jana, Pal & Wang (2019), and Riaz et al. (2022) introduced various aggregation operators (AOs) in the setting of BFS. Alghamdi, Alshehri & Akram (2018) studied a MADM (multi-attribute DM) approach for BFS. Akram & Akmal (2016) studied the application of BFS in the model of the graph. Samanta & Pal (2012) introduced the BF hypergraph. Bi et al. (2019) investigated arithmetic and Bi, Dai & Hu (2018) studied geometric AOs under CFS. The notion of BCFS is utilized in numerous fields such as pattern recognition (Ur Rehman & Mahmood, 2022) and medical diagnosis (Ur Rehman & Mahmood, 2022), and numerous DM approaches are in the structure of BCF information such as Mahmood et al. (2021) investigated the MADM procedure, Mahmood et al. (2022b) studied decision support systems, Mahmood & Ur Rehman (2022a) introduced MADM relying on Dombi AOs, and Rehman et al. (2022) devised the Analytical Hierarchy process. Furthermore, Mahmood, Rehman & Ali (2022) deduced Aczel-Alina, Mahmood & Ur Rehman (2022b) originated the Maclurin symmetric mean, Mahmood et al. (2022a) introduced the Bonferroni mean, and Mahmood, Rehman & Naeem (2023) introduced Heronian mean AOs based on the BCF information.

### Motivation

The AOs depend on various sorts of operators and t-norms and t-conorms (TTs) like prioritized operators interpreted by Yager (2008), Schweizer-Sklar deduced by Deschrijver & Kerre (2002), the Bonferroni mean presented by Bonferroni (1950), the Maclaurin mean investigated by Maclaurin (1729), the Hamacher originated by Hamacher (1978) and the Dombi TTs deduced by Dombi (1982). In particular, the SS t-norm and t-conorm Deschrijver & Kerre (2002) contain a variable parameter that affects the related operations and makes them more flexible than the rest of the operators. Numerous authors utilized the SS t-norm and t-conorm to investigate AOs in various areas, as Liu & Wang (2018) introduced SS AOs for interval-valued intuitionistic FS (IVIFS), Biswas & Deb (2021) investigated SS AOs for Pythagorean FS, Tian et al. (2022) investigated for picture FS and Liu, Khan & Mahmood (2019) deduced for neutrosophic FS. Furthermore, the prioritized operators (PROs) can consider the prioritization relatedness over attributes or criteria and make the AOs more effective and useful. The prioritization among attributes can be structured by developing the weights linked to the attributes relying on the fulfillment of the superior priority criteria. Here, a concern arises of what would be happened if the decision-maker or expert has to consider both poles of the objects and extra fuzzy information in a single structure and need a variable parameter as well as the prioritization relatedness over attributes or criteria to make the aggregation procedure more effective and beneficial. None of the AOs and notions in the prevailing literature can cope with such situations and DM issues involving such sort of information and need variable parameters as well as the prioritization relatedness over attributes or criteria in the aggregation of the information. To remove this concern and fill this research gap in this script, we investigate:

• the prioritized AOs by employing SS t-norm and t-conorm in the setting of BCFS such as BCF Schweizer-Sklar prioritized weighted averaging (BCFSSPRWA), BCF Schweizer-Sklar prioritized ordered weighted averaging (BCFSSPROWA), BCF Schweizer-Sklar prioritized weighted geometric (BCFSSPRWG), and BCF Schweizer-Sklar prioritized ordered weighted geometric (BCFSSPRWG) operators;
• a technique of DM under the environment of BCFS by employing the investigated AOs.
• Further, we analyze mental disorders and rank the most prevalent types of mental disorders by employing the notion of BCF decision-making technique depending on the introduced operators.

The introduced work is the generalization of various prevailing ideas in the literature such as FS, BFS, and CFS. Thus, the introduced AOs can easily aggregate the information interpreted in the setting of FS, BFS, and CFS and the introduced DM technique can cope with DM issues under the environment of FS, BFS, and CFS.

‘Literature Review’ contains the basic review of the mental disorder, BCFS, prioritized operators, and SS t-norm and t-conorms. ‘Preliminaries’ contains the SS operational laws relying on BCF numbers (BCFNs) and BCFSSPRWA, BCFSSPROWA, BCFSSPRWG, and BCFSSPROWG operators based on these SS operational laws. ‘BCF Schweizer-Sklar Prioritized AOs’ contains the ranking of the kinds of mental disorder with the help of the approach of DM based on the introduced operators in the setting of BCF information. ‘Comparative Study’ contains the comparative study and ‘Conclusion’ contains the conclusion of this article.

## Preliminaries

In this part of the article, we analyze the mental disorder, BCFS, and its basic notions, prioritized operators, and SS t-norm and t-conorms.

### Mental disorder

A broad variety of psychiatric conditions that influence your feelings, thoughts, and conduct patterns are described as mental diseases, occasionally identified as mental health disorders. Recession, social phobia, schizophrenic psychosis, eating disorders, and behavioral addictions are certain examples of mental disorders. In certain circumstances, many people fight for their psychological health. But when alarming signs persist and frequently causes alarm and weaken your ability to execute tasks, a mental health issue becomes a mental disorder. One may experience unhappiness as a consequence of a mental disorder, which can also affect everyday activities involving interpersonal relationships, jobs, and academics. For the majority of the time, a mix of drugs and behavioral therapy improves managing symptoms (psychotherapy).

### Symptoms

Varying on the problems, the setting, and other elements, there can be a broad range of indications and manifestations of mental disorders. Emotional responses, beliefs, and behavior patterns can be impacted by the symptoms of mental disorders. Indicators and symptoms, for instance, include (1) issues with comprehension and interpersonal relationships, (2) intense wrath, fury, or aggression, (3) disruption of realism (fantasies), doubt, or mental confusion, (4) suicidality, (5) extreme concern, uneasiness, or regret (6), significant dietary alterations, (7) excessive exhaustion, extreme fatigue, or sleeping issues, (8) being unable to deal with stress or daily difficulties, (9) feeling low or sad, (10) up and downs of mood disturbances, (11) sexual desire modification, (12) alcoholism or drug use issue, (13) thinking that is jumbled up or has diminished focus, (14) abandoning people and interests.

Medical ailments like headaches, backaches, stomachaches, or other inexplicable sensations and discomfort can occasionally be mistaken for signs of mental illness.

### Causes

Numerous social and hereditary variables are known to play a role in the development of mental diseases in general:

• **Cognitive chemistry:** The brain’s chemical messengers, known as neurotransmitters, send indicators to several regions of the body and mind. The functionality of neurotransmitters and nervous structures shifts when the brain pathways containing these substances are negotiated, which creates anxiety and other mental issue.
• **Inherited traits:** Individuals with mental disorders possibly have biological relations who are equally suffering. Your situation as well as certain traits may both improve your risk of mental disease.
• **Social exposure before birth:** The mental disease may occasionally be connected to prenatal contact with external chronic stress, inflammatory disorders, chemicals, alcoholism, or narcotics.

### Prevention

Mental disorders cannot be entirely avoided. However, if you suffer from a mental disorder, managing anxiety, building tolerance, and improving confidence can all assist you in keeping your symptoms in check. The following actions should be taken:

• **Ask for help when in need:** If you hesitate unless signs become extreme, it may be more difficult to manage mental health issues. Maintenance period therapy may also actually prevent the return of a symptom.
• **Take good care of yourself:** A healthy lifestyle includes sleeping properly, consuming food well, and working out frequently. Maintain a consistent plan as much as you can. If you have worries about food and exercise, or when you have problems dropping asleep, ask your healthcare doctor.
• **Routine check-ups:** If you do not feel healthy, do not avoid checkups or appointments with your primary care physician. It is possible that you will have to be examined for brand-new health issues or that you are dealing with pharmaceutical side effects.
• **Considering alarming signs:** To determine what might cause you problems, talk to a physician or psychotherapist. Develop a plan thus you will recognize how to proceed if your signs reoccur. If your signs or feelings alter, consult with your physician or psychotherapist. To look out for warning indicators, think about enlisting the help of family or friends.

### Bipolar complex fuzzy set

**Definition 1: (Mahmood & Rehman, 2022)** Consider the following structure:

(1) \begin{eqnarray*}\Xi & = \left\{ \left( \zeta , \left( {\mathfrak{Y}}_{\Xi }^{P} \left( \zeta \right) ,{\mathfrak{Y}}_{\Xi }^{N} \left( \zeta \right) \right) \right) {|}\zeta \in \Pi \right\} \nonumber\\\displaystyle & = \left\{ \left( \zeta , \left( {\mathfrak{Y}}_{\Xi }^{RP} \left( \zeta \right) +\iota {\mathfrak{Y}}_{\Xi }^{IP} \left( \zeta \right) ,{\mathfrak{Y}}_{\Xi }^{RN} \left( \zeta \right) +\iota {\mathfrak{Y}}_{\Xi }^{IN} \left( \zeta \right) \right) \right) {|}\zeta \in \Pi \right\} \end{eqnarray*}

where Π is a set, ${\mathfrak{Y}}_{\Xi }^{P} \left( \zeta \right) $ is a positive truth degree, ${\mathfrak{Y}}_{\Xi }^{N} \left( \zeta \right) $ is a negative truth degree contained in a unit square of a complex plane, then Ξ is interpreted as BCFS. The set $\Xi = \left( {\mathfrak{Y}}_{\Xi }^{P},{\mathfrak{Y}}_{\Xi }^{N} \right) = \left( {\mathfrak{Y}}_{\Xi }^{RP}+\iota {\mathfrak{Y}}_{\Xi }^{IP},{\mathfrak{Y}}_{\Xi }^{RN}+\iota {\mathfrak{Y}}_{\Xi }^{IN} \right) $ is interpreted as BCFN.

**Definition 2: (Mahmood & Ur Rehman, 2022a)** Consider ${\Xi }_{1}= \left( {\mathfrak{Y}}_{{\Xi }_{1}}^{P},{\mathfrak{Y}}_{{\Xi }_{1}}^{N} \right) $ $= \left( {\mathfrak{Y}}_{{\Xi }_{1}}^{RP}+\iota {\mathfrak{Y}}_{{\Xi }_{1}}^{IP},{\mathfrak{Y}}_{{\Xi }_{1}}^{RN}+\iota {\mathfrak{Y}}_{{\Xi }_{1}}^{IN} \right) $ and ${\Xi }_{2}= \left( {\mathfrak{Y}}_{{\Xi }_{2}}^{P},{\mathfrak{Y}}_{{\Xi }_{2}}^{N} \right) = \left( {\mathfrak{Y}}_{{\Xi }_{2}}^{RP}+\iota {\mathfrak{Y}}_{{\Xi }_{2}}^{IP},{\mathfrak{Y}}_{{\Xi }_{2}}^{RN}+\iota {\mathfrak{Y}}_{{\Xi }_{2}}^{IN} \right) $, as two BCFNs with *α*′ ≥ 0, then we have

1.
${\Xi }_{1}\oplus {\Xi }_{2}= \left( {\scriptsize \begin{array}{@{}c@{}} \displaystyle {\mathfrak{Y}}_{{\Xi }_{1}}^{RP}+{\mathfrak{Y}}_{{\Xi }_{2}}^{RP}-{\mathfrak{Y}}_{{\Xi }_{1}}^{RP}{\mathfrak{Y}}_{{\Xi }_{2}}^{RP}\\ \displaystyle +~\iota \left( {\mathfrak{Y}}_{{\Xi }_{1}}^{IP}+{\mathfrak{Y}}_{{\Xi }_{2}}^{IP}-{\mathfrak{Y}}_{{\Xi }_{1}}^{IP}{\mathfrak{Y}}_{{\Xi }_{2}}^{IP} \right) ,\\ \displaystyle - \left( {\mathfrak{Y}}_{{\Xi }_{1}}^{RN}{\mathfrak{Y}}_{{\Xi }_{2}}^{RN} \right) +\iota \left( - \left( {\mathfrak{Y}}_{{\Xi }_{1}}^{IN}{\mathfrak{Y}}_{{\Xi }_{2}}^{IN} \right) \right) \end{array}} \right) $

2.
${\Xi }_{1}\otimes {\Xi }_{2}= \left( {\scriptsize \begin{array}{@{}c@{}} \displaystyle {\mathfrak{Y}}_{{\Xi }_{1}}^{RP}{\mathfrak{Y}}_{{\Xi }_{2}}^{RP}+\iota {\mathfrak{Y}}_{{\Xi }_{1}}^{IP}{\mathfrak{Y}}_{{\Xi }_{2}}^{IP},\\ \displaystyle {\mathfrak{Y}}_{{\Xi }_{1}}^{RN}+{\mathfrak{Y}}_{{\Xi }_{2}}^{RN}+{\mathfrak{Y}}_{{\Xi }_{1}}^{RN}{\mathfrak{Y}}_{{\Xi }_{2}}^{RN}\\ \displaystyle \iota \left( {\mathfrak{Y}}_{{\Xi }_{1}}^{IN}+{\mathfrak{Y}}_{{\Xi }_{2}}^{IN}+{\mathfrak{Y}}_{{\Xi }_{1}}^{IN}{\mathfrak{Y}}_{{\Xi }_{2}}^{IN} \right) \end{array}} \right) $

3.
${\Xi }_{1}= \left( {\scriptsize \begin{array}{@{}c@{}} \displaystyle 1-{ \left( 1-{\mathfrak{Y}}_{{\Xi }_{1}}^{RP} \right) }^{{\alpha }^{{^{\prime}}}}+\iota \left( 1-{ \left( 1-{\mathfrak{Y}}_{{\Xi }_{1}}^{IP} \right) }^{{\alpha }^{{^{\prime}}}} \right) ,\\ \displaystyle -{ \left\vert {\mathfrak{Y}}_{{\Xi }_{1}}^{RN} \right\vert }^{{\alpha }^{{^{\prime}}}}+\iota \left( -{ \left\vert {\mathfrak{Y}}_{{\Xi }_{1}}^{IN} \right\vert }^{{\alpha }^{{^{\prime}}}} \right) \end{array}} \right) $

4. ${\Xi }_{1}^{{\alpha }^{{^{\prime}}}}= \left( {\scriptsize \begin{array}{@{}c@{}} \displaystyle { \left( {\mathfrak{Y}}_{{\Xi }_{1}}^{RP} \right) }^{{\alpha }^{{^{\prime}}}}+\iota { \left( {\mathfrak{Y}}_{{\Xi }_{1}}^{IP} \right) }^{{\alpha }^{{^{\prime}}}},\\ \displaystyle -1+{ \left( 1+{\mathfrak{Y}}_{{\Xi }_{1}}^{RN} \right) }^{{\alpha }^{{^{\prime}}}}+\iota \left( -1+{ \left( 1+{\mathfrak{Y}}_{{\Xi }_{1}}^{IN} \right) }^{{\alpha }^{{^{\prime}}}} \right) \end{array}} \right) $.

**Definition 3: (Mahmood & Ur Rehman, 2022a)** Consider a BCFN $\Xi = \left( {\mathfrak{Y}}_{\Xi }^{P},{\mathfrak{Y}}_{\Xi }^{N} \right) = \left( {\mathfrak{Y}}_{\Xi }^{RP}+\iota {\mathfrak{Y}}_{\Xi }^{IP},{\mathfrak{Y}}_{\Xi }^{RN}+\iota {\mathfrak{Y}}_{\Xi }^{IN} \right) $, then the score value is introduced as

(2) \begin{eqnarray*}{\mathfrak{S}}_{\mathfrak{B}} \left( \Xi \right) = \frac{1}{4} \left( 2+{\mathfrak{Y}}_{\Xi }^{RP}+{\mathfrak{Y}}_{\Xi }^{IP}+{\mathfrak{Y}}_{\Xi }^{RN}+{\mathfrak{Y}}_{\Xi }^{IN} \right) ,{\mathfrak{S}}_{\mathfrak{B}} \left( \Xi \right) \in \left[ 0,1 \right] \end{eqnarray*}

**Definition 4: (Mahmood & Ur Rehman, 2022a)** Consider a BCFN $\Xi = \left( {\mathfrak{Y}}_{\Xi }^{P},{\mathfrak{Y}}_{\Xi }^{N} \right) = \left( {\mathfrak{Y}}_{\Xi }^{RP}+\iota {\mathfrak{Y}}_{\Xi }^{IP},{\mathfrak{Y}}_{\Xi }^{RN}+\iota {\mathfrak{Y}}_{\Xi }^{IN} \right) $, then the accuracy value is introduced as

(3) \begin{eqnarray*}{\mathfrak{H}}_{\mathfrak{B}} \left( \Xi \right) = \frac{{\mathfrak{Y}}_{\Xi }^{RP}+{\mathfrak{Y}}_{\Xi }^{IP}-{\mathfrak{Y}}_{\Xi }^{RN}-{\mathfrak{Y}}_{\Xi }^{IN}}{4} ,{\mathfrak{H}}_{\mathfrak{B}} \left( \Xi \right) \in \left[ 0,1 \right] \end{eqnarray*}

### PRA operator

**Definition 5: (Yager, 2008)** Consider $\mathfrak{C}= \left\{ {\mathfrak{C}}_{1},{\mathfrak{C}}_{2},\ldots ,{\mathfrak{C}}_{\check {\mathrm{k}}} \right\} $ as a group of attributes and take a prioritization between these attributes interpreted by a linear order that is ${\mathfrak{C}}_{1}> {\mathfrak{C}}_{2}> \ldots > {\mathfrak{C}}_{\check {\mathrm{k}}}$. This means that if ȅ $< \breve {\mathrm{g}}$, then ℭ_*ȅ*_ is prior than ${\mathfrak{C}}_{\breve {\mathrm{g}}}$. ${\mathfrak{C}}_{e} \left( \Xi \right) $ is the assessment argument describing the alternative’s performance by keeping in view the attribute ℭ_𝔄𝔱−̦*e*_ and holding ${\mathfrak{C}}_{\mathfrak{A}\mathfrak{t}-e}\in \left[ 0,1 \right] $. If

(4) \begin{eqnarray*}PRA \left( {\mathfrak{C}}_{1} \left( \Xi \right) ,{\mathfrak{C}}_{2} \left( \Xi \right) ,\ldots ,{\mathfrak{C}}_{\mathrm{J}} \left( \Xi \right) \right) =\begin{array}{@{}c@{}} \displaystyle \check {\mathrm{k}}\\ \displaystyle \oplus \\ \displaystyle e=1 \end{array} \frac{{\mathrm{\v{T} }}_{e}}{\sum _{e=1}^{\check {\mathrm{k}}}{\mathrm{\v{T} }}_{e}} {\mathfrak{C}}_{e} \left( \Xi \right) \end{eqnarray*}

### Schweizer-Sklar operations

**Definition 6: (Deschrijver & Kerre, 2002)** The underneath equations interpret the SS t-norm and t-conorm, respectively, where $\mathfrak{m},\mathfrak{n}\in \left[ 0,1 \right] $ and ℷ < 0.

(5) \begin{eqnarray*}{\mathfrak{V}}_{\mathbb{SS}} \left( \mathfrak{m},\mathfrak{n} \right) ={ \left( {\mathfrak{m}}^{\mathrm{\gimel }}+{\mathfrak{n}}^{\mathrm{\gimel }}-1 \right) }^{ \frac{1}{\mathrm{\gimel }} }\end{eqnarray*}

(6) \begin{eqnarray*}{\mathfrak{V}}_{\mathbb{SS}}^{\ast } \left( \mathfrak{m},\mathfrak{n} \right) =1-{ \left( { \left( 1-\mathfrak{m} \right) }^{\mathrm{\gimel }}+{ \left( 1-\mathfrak{n} \right) }^{\mathrm{\gimel }}-1 \right) }^{ \frac{1}{\mathrm{\gimel }} }\end{eqnarray*}

### BCF Schweizer-Sklar prioritized AOs

This part of the article contains the SS operational laws relying on the SS t-norm and t-conorm under the environment of BCF information. Furthermore, we invent BCFSSPRWA, BCFSSPROWA, BCFSSPRWG, and BCFSSPROWG operators based on these SS operational laws.

**Definition 7:** Consider ${\Xi }_{1}= \left( {\mathfrak{Y}}_{{\Xi }_{1}}^{P},{\mathfrak{Y}}_{{\Xi }_{1}}^{N} \right) = \left( {\mathfrak{Y}}_{{\Xi }_{1}}^{RP}+\iota {\mathfrak{Y}}_{{\Xi }_{1}}^{IP},{\mathfrak{Y}}_{{\Xi }_{1}}^{RN}+\iota {\mathfrak{Y}}_{{\Xi }_{1}}^{IN} \right) $ and ${\Xi }_{2}= \left( {\mathfrak{Y}}_{{\Xi }_{2}}^{P},{\mathfrak{Y}}_{{\Xi }_{2}}^{N} \right) = \left( {\mathfrak{Y}}_{{\Xi }_{2}}^{RP}+\iota {\mathfrak{Y}}_{{\Xi }_{2}}^{IP},{\mathfrak{Y}}_{{\Xi }_{2}}^{RN}+\iota {\mathfrak{Y}}_{{\Xi }_{2}}^{IN} \right) $, are two BCFNs with ℷ < 0 and *α*′ > 0 then we investigate the BCF SS operational laws as

1.
${\Xi }_{1}{\oplus }_{\mathbb{SS}}{\Xi }_{2}= \left( {\scriptsize \begin{array}{@{}c@{}} \displaystyle 1-{ \left( { \left( 1-{\mathfrak{Y}}_{{\Xi }_{1}}^{RP} \right) }^{\mathrm{\gimel }}+{ \left( 1-{\mathfrak{Y}}_{{\Xi }_{2}}^{RP} \right) }^{\mathrm{\gimel }}-1 \right) }^{ \frac{1}{\mathrm{\gimel }} }\\ \displaystyle +\iota \left( 1-{ \left( { \left( 1-{\mathfrak{Y}}_{{\Xi }_{1}}^{IP} \right) }^{\mathrm{\gimel }}+{ \left( 1-{\mathfrak{Y}}_{{\Xi }_{2}}^{IP} \right) }^{\mathrm{\gimel }}-1 \right) }^{ \frac{1}{\mathrm{\gimel }} } \right) ,\\ \displaystyle -{ \left( { \left\vert {\mathfrak{Y}}_{{\Xi }_{1}}^{RN} \right\vert }^{\mathrm{\gimel }}+{ \left\vert {\mathfrak{Y}}_{{\Xi }_{2}}^{RN} \right\vert }^{\mathrm{\gimel }}-1 \right) }^{ \frac{1}{\mathrm{\gimel }} }+\iota \left( -{ \left( { \left\vert {\mathfrak{Y}}_{{\Xi }_{1}}^{IN} \right\vert }^{\mathrm{\gimel }}+{ \left\vert {\mathfrak{Y}}_{{\Xi }_{2}}^{IN} \right\vert }^{\mathrm{\gimel }}-1 \right) }^{ \frac{1}{\mathrm{\gimel }} } \right) \end{array}} \right) $

2.
${\Xi }_{1}{\otimes }_{\mathbb{SS}}{\Xi }_{2}= \left( {\scriptsize \begin{array}{@{}c@{}} \displaystyle { \left( { \left( {\mathfrak{Y}}_{{\Xi }_{1}}^{RP} \right) }^{\mathrm{\gimel }}+{ \left( {\mathfrak{Y}}_{{\Xi }_{2}}^{RP} \right) }^{\mathrm{\gimel }}-1 \right) }^{ \frac{1}{\mathrm{\gimel }} }+\iota \left( { \left( { \left( {\mathfrak{Y}}_{{\Xi }_{1}}^{IP} \right) }^{\mathrm{\gimel }}+{ \left( {\mathfrak{Y}}_{{\Xi }_{2}}^{IP} \right) }^{\mathrm{\gimel }}-1 \right) }^{ \frac{1}{\mathrm{\gimel }} } \right) ,\\ \displaystyle -1+{ \left( { \left( 1+{\mathfrak{Y}}_{{\Xi }_{1}}^{RN} \right) }^{\mathrm{\gimel }}+{ \left( 1+{\mathfrak{Y}}_{{\Xi }_{2}}^{RN} \right) }^{\mathrm{\gimel }}-1 \right) }^{ \frac{1}{\mathrm{\gimel }} }\\ \displaystyle +\iota \left( -1+{ \left( { \left( 1+{\mathfrak{Y}}_{{\Xi }_{1}}^{RN} \right) }^{\mathrm{\gimel }}+{ \left( 1+{\mathfrak{Y}}_{{\Xi }_{2}}^{RN} \right) }^{\mathrm{\gimel }}-1 \right) }^{ \frac{1}{\mathrm{\gimel }} } \right) \end{array}} \right) $

3.
${\alpha }^{{^{\prime}}}{\Xi }_{1}= \left( {\scriptsize \begin{array}{@{}c@{}} \displaystyle 1-{ \left( {\alpha }^{{^{\prime}}}{ \left( 1-{\mathfrak{Y}}_{{\Xi }_{1}}^{RP} \right) }^{\mathrm{\gimel }}- \left( {\alpha }^{{^{\prime}}}-1 \right) \right) }^{ \frac{1}{\mathrm{\gimel }} }+\iota \left( 1-{ \left( {\alpha }^{{^{\prime}}}{ \left( 1-{\mathfrak{Y}}_{{\Xi }_{1}}^{IP} \right) }^{\mathrm{\gimel }}- \left( {\alpha }^{{^{\prime}}}-1 \right) \right) }^{ \frac{1}{\mathrm{\gimel }} } \right) ,\\ \displaystyle -{ \left( {\alpha }^{{^{\prime}}}{ \left\vert {\mathfrak{Y}}_{{\Xi }_{1}}^{RN} \right\vert }^{\mathrm{\gimel }}- \left( {\alpha }^{{^{\prime}}}-1 \right) \right) }^{ \frac{1}{\mathrm{\gimel }} }+\iota \left( -{ \left( {\alpha }^{{^{\prime}}}{ \left\vert {\mathfrak{Y}}_{{\Xi }_{1}}^{IN} \right\vert }^{\mathrm{\gimel }}- \left( {\alpha }^{{^{\prime}}}-1 \right) \right) }^{ \frac{1}{\mathrm{\gimel }} } \right) \end{array}} \right) $

4.
${ \left( {\Xi }_{1} \right) }^{{\alpha }^{{^{\prime}}}}= \left( {\scriptsize \begin{array}{@{}c@{}} \displaystyle { \left( {\alpha }^{{^{\prime}}}{ \left( {\mathfrak{Y}}_{{\Xi }_{1}}^{RP} \right) }^{\mathrm{\gimel }}- \left( {\alpha }^{{^{\prime}}}-1 \right) \right) }^{ \frac{1}{\mathrm{\gimel }} }+\iota { \left( {\alpha }^{{^{\prime}}}{ \left( {\mathfrak{Y}}_{{\Xi }_{1}}^{IP} \right) }^{\mathrm{\gimel }}- \left( {\alpha }^{{^{\prime}}}-1 \right) \right) }^{ \frac{1}{\mathrm{\gimel }} },\\ \displaystyle -1+{ \left( {\alpha }^{{^{\prime}}}{ \left( 1+{\mathfrak{Y}}_{{\Xi }_{1}}^{RN} \right) }^{\mathrm{\gimel }}- \left( {\alpha }^{{^{\prime}}}-1 \right) \right) }^{ \frac{1}{\mathrm{\gimel }} }+\iota \left( -1+{ \left( {\alpha }^{{^{\prime}}}{ \left( 1+{\mathfrak{Y}}_{{\Xi }_{1}}^{IN} \right) }^{\mathrm{\gimel }}- \left( {\alpha }^{{^{\prime}}}-1 \right) \right) }^{ \frac{1}{\mathrm{\gimel }} } \right) \end{array}} \right) $

**Definition 8:** Consider ${\Xi }_{e}= \left( {\mathfrak{Y}}_{{\Xi }_{e}}^{P},{\mathfrak{Y}}_{{\Xi }_{e}}^{N} \right) = \left( {\mathfrak{Y}}_{{\Xi }_{e}}^{RP}+\iota {\mathfrak{Y}}_{{\Xi }_{e}}^{IP},{\mathfrak{Y}}_{{\Xi }_{e}}^{RN}+\iota {\mathfrak{Y}}_{{\Xi }_{e}}^{IN} \right) ,e=1,2,..,\check {\mathrm{k}}$ as a group of BCFNs, then

(7) \begin{eqnarray*}BCFSSPRWA \left( {\Xi }_{1},{\Xi }_{2},\ldots ,{\Xi }_{\check {\mathrm{k}}} \right) =\begin{array}{@{}c@{}} \displaystyle \check {\mathrm{k}}\\ \displaystyle \oplus \\ \displaystyle e=1 \end{array} \frac{{\mathrm{\v{T} }}_{e}}{\sum _{e=1}^{\check {\mathrm{k}}}{\mathrm{\v{T} }}_{e}} {\Xi }_{e}\end{eqnarray*}

is introduced as BCFSSPRWA operator, where Ť_1_ = 1, ${\mathrm{\v{T} }}_{e}={\mathop{\prod }\nolimits }_{e=1}^{\check {\mathrm{k}}-1}{\mathfrak{S}}_{\mathfrak{B}} \left( {\Xi }_{e} \right) ,e=1,2,\ldots ,\check {\mathrm{k}}$ and ${\mathfrak{S}}_{\mathfrak{B}} \left( {\Xi }_{e} \right) $ would deduce the score value of BCFN Ξ_*ȅ*_.

**Theorem 1:** Consider ${\Xi }_{e}= \left( {\mathfrak{Y}}_{{\Xi }_{e}}^{P},{\mathfrak{Y}}_{{\Xi }_{e}}^{N} \right) = \left( {\mathfrak{Y}}_{{\Xi }_{e}}^{RP}+\iota {\mathfrak{Y}}_{{\Xi }_{e}}^{IP},{\mathfrak{Y}}_{{\Xi }_{e}}^{RN}+\iota {\mathfrak{Y}}_{{\Xi }_{e}}^{IN} \right) ,e=1,2,\ldots ,\check {\mathrm{k}}$ as a group of BCFNs, then by utilizing BCFSSPRWA, we achieve the aggregated result and

(8) \begin{eqnarray*}BCFSSPRWA \left( {\Xi }_{1},{\Xi }_{2},\ldots ,{\Xi }_{\check {\mathrm{k}}} \right) \nonumber\\\displaystyle = \left( \begin{array}{@{}c@{}} \displaystyle 1-{ \left( \sum _{e=1}^{\check {\mathrm{k}}} \frac{{\mathrm{\v{T} }}_{e}}{\sum _{e=1}^{\check {\mathrm{k}}}{\mathrm{\v{T} }}_{e}} { \left( 1-{\mathfrak{Y}}_{{\Xi }_{e}}^{RP} \right) }^{\mathrm{\gimel }} \right) }^{ \frac{1}{\mathrm{\gimel }} }+\iota \left( 1-{ \left( \sum _{e=1}^{\check {\mathrm{k}}} \frac{{\mathrm{\v{T} }}_{e}}{\sum _{e=1}^{\check {\mathrm{k}}}{\mathrm{\v{T} }}_{e}} { \left( 1-{\mathfrak{Y}}_{{\Xi }_{e}}^{IP} \right) }^{\mathrm{\gimel }} \right) }^{ \frac{1}{\mathrm{\gimel }} } \right) ,\\ \displaystyle -{ \left( \sum _{e=1}^{\check {\mathrm{k}}} \frac{{\mathrm{\v{T} }}_{e}}{\sum _{e=1}^{\check {\mathrm{k}}}{\mathrm{\v{T} }}_{e}} { \left\vert {\mathfrak{Y}}_{{\Xi }_{e}}^{RN} \right\vert }^{\mathrm{\gimel }} \right) }^{ \frac{1}{\mathrm{\gimel }} }+\iota \left( -{ \left( \sum _{e=1}^{\check {\mathrm{k}}} \frac{{\mathrm{\v{T} }}_{e}}{\sum _{e=1}^{\check {\mathrm{k}}}{\mathrm{\v{T} }}_{e}} { \left\vert {\mathfrak{Y}}_{{\Xi }_{e}}^{IN} \right\vert }^{\mathrm{\gimel }} \right) }^{ \frac{1}{\mathrm{\gimel }} } \right) \end{array} \right) \end{eqnarray*}

**Proof:** Equation (8) can be rewritten as underneath

(9) \begin{eqnarray*}BCFSSPWA \left( {\Xi }_{1},{\Xi }_{2},\ldots ,{\Xi }_{\check {\mathrm{k}}} \right) \nonumber\\\displaystyle = \left( \begin{array}{@{}c@{}} \displaystyle 1-{ \left( \sum _{e=1}^{\check {\mathrm{k}}} \frac{{\mathrm{\v{T} }}_{e}}{\sum _{e=1}^{\check {\mathrm{k}}}{\mathrm{\v{T} }}_{e}} { \left( 1-{\mathfrak{Y}}_{{\Xi }_{e}}^{RP} \right) }^{\mathrm{\gimel }}-\sum _{e=1}^{\check {\mathrm{k}}} \frac{{\mathrm{\v{T} }}_{e}}{\sum _{e=1}^{\check {\mathrm{k}}}{\mathrm{\v{T} }}_{e}} +1 \right) }^{ \frac{1}{\mathrm{\gimel }} }\\ \displaystyle +\iota \left( 1-{ \left( \sum _{e=1}^{\check {\mathrm{k}}} \frac{{\mathrm{\v{T} }}_{e}}{\sum _{e=1}^{\check {\mathrm{k}}}{\mathrm{\v{T} }}_{e}} { \left( 1-{\mathfrak{Y}}_{{\Xi }_{e}}^{IP} \right) }^{\mathrm{\gimel }}-\sum _{e=1}^{\check {\mathrm{k}}} \frac{{\mathrm{\v{T} }}_{e}}{\sum _{e=1}^{\check {\mathrm{k}}}{\mathrm{\v{T} }}_{e}} +1 \right) }^{ \frac{1}{\mathrm{\gimel }} } \right) ,\\ \displaystyle -{ \left( \sum _{e=1}^{\check {\mathrm{k}}} \frac{{\mathrm{\v{T} }}_{e}}{\sum _{e=1}^{\check {\mathrm{k}}}{\mathrm{\v{T} }}_{e}} { \left\vert {\mathfrak{Y}}_{{\Xi }_{e}}^{RN} \right\vert }^{\mathrm{\gimel }}-\sum _{e=1}^{\check {\mathrm{k}}} \frac{{\mathrm{\v{T} }}_{e}}{\sum _{e=1}^{\check {\mathrm{k}}}{\mathrm{\v{T} }}_{e}} +1 \right) }^{ \frac{1}{\mathrm{\gimel }} }\\ \displaystyle +\iota \left( -{ \left( \sum _{e=1}^{\check {\mathrm{k}}} \frac{{\mathrm{\v{T} }}_{e}}{\sum _{e=1}^{\check {\mathrm{k}}}{\mathrm{\v{T} }}_{e}} { \left\vert {\mathfrak{Y}}_{{\Xi }_{e}}^{IN} \right\vert }^{\mathrm{\gimel }} \right) }^{ \frac{1}{\mathrm{\gimel }} }-\sum _{e=1}^{\check {\mathrm{k}}} \frac{{\mathrm{\v{T} }}_{e}}{\sum _{e=1}^{\check {\mathrm{k}}}{\mathrm{\v{T} }}_{e}} +1 \right) \end{array} \right) \end{eqnarray*}

Through mathematical induction, we would exhibit Eq. (9) is holds $\forall \check {\mathrm{k}}$. Let $\check {\mathrm{k}}=2$. Then

\begin{eqnarray*} \frac{{\mathrm{\v{T} }}_{1}}{\sum _{e=1}^{2}{\mathrm{\v{T} }}_{e}} {\Xi }_{1}= \left( \begin{array}{@{}c@{}} \displaystyle 1-{ \left( \frac{{\mathrm{\v{T} }}_{1}}{\sum _{e=1}^{2}{\mathrm{\v{T} }}_{e}} { \left( 1-{\mathfrak{Y}}_{{\Xi }_{1}}^{RP} \right) }^{\mathrm{\gimel }}- \left( {\mathfrak{K}}_{1}-1 \right) \right) }^{ \frac{1}{\mathrm{\gimel }} }\\ \displaystyle +\iota \left( 1-{ \left( \frac{{\mathrm{\v{T} }}_{1}}{\sum _{e=1}^{2}{\mathrm{\v{T} }}_{e}} { \left( 1-{\mathfrak{Y}}_{{\Xi }_{1}}^{IP} \right) }^{\mathrm{\gimel }}- \left( {\mathfrak{K}}_{1}-1 \right) \right) }^{ \frac{1}{\mathrm{\gimel }} } \right) ,\\ \displaystyle -{ \left( \frac{{\mathrm{\v{T} }}_{1}}{\sum _{e=1}^{2}{\mathrm{\v{T} }}_{e}} { \left\vert {\mathfrak{Y}}_{{\Xi }_{1}}^{RN} \right\vert }^{\mathrm{\gimel }}- \left( {\mathfrak{K}}_{1}-1 \right) \right) }^{ \frac{1}{\mathrm{\gimel }} }\\ \displaystyle +\iota \left( -{ \left( \frac{{\mathrm{\v{T} }}_{1}}{\sum _{e=1}^{2}{\mathrm{\v{T} }}_{e}} { \left\vert {\mathfrak{Y}}_{{\Xi }_{1}}^{IN} \right\vert }^{\mathrm{\gimel }}- \left( {\mathfrak{K}}_{1}-1 \right) \right) }^{ \frac{1}{\mathrm{\gimel }} } \right) \end{array} \right) \end{eqnarray*}

and

\begin{eqnarray*} \frac{{\mathrm{\v{T} }}_{2}}{\sum _{e=1}^{2}{\mathrm{\v{T} }}_{e}} {\Xi }_{2}= \left( \begin{array}{@{}c@{}} \displaystyle 1-{ \left( \frac{{\mathrm{\v{T} }}_{2}}{\sum _{e=1}^{2}{\mathrm{\v{T} }}_{e}} { \left( 1-{\mathfrak{Y}}_{{\Xi }_{2}}^{RP} \right) }^{\mathrm{\gimel }}- \left( {\mathfrak{K}}_{2}-1 \right) \right) }^{ \frac{1}{\mathrm{\gimel }} }\\ \displaystyle +\iota \left( 1-{ \left( \frac{{\mathrm{\v{T} }}_{2}}{\sum _{e=1}^{2}{\mathrm{\v{T} }}_{e}} { \left( 1-{\mathfrak{Y}}_{{\Xi }_{2}}^{IP} \right) }^{\mathrm{\gimel }}- \left( {\mathfrak{K}}_{2}-1 \right) \right) }^{ \frac{1}{\mathrm{\gimel }} } \right) ,\\ \displaystyle -{ \left( \frac{{\mathrm{\v{T} }}_{2}}{\sum _{e=1}^{2}{\mathrm{\v{T} }}_{e}} { \left\vert {\mathfrak{Y}}_{{\Xi }_{2}}^{RN} \right\vert }^{\mathrm{\gimel }}- \left( {\mathfrak{K}}_{2}-1 \right) \right) }^{ \frac{1}{\mathrm{\gimel }} }\\ \displaystyle +\iota \left( -{ \left( \frac{{\mathrm{\v{T} }}_{2}}{\sum _{e=1}^{2}{\mathrm{\v{T} }}_{e}} { \left\vert {\mathfrak{Y}}_{{\Xi }_{2}}^{IN} \right\vert }^{\mathrm{\gimel }}- \left( {\mathfrak{K}}_{2}-1 \right) \right) }^{ \frac{1}{\mathrm{\gimel }} } \right) \end{array} \right) \end{eqnarray*}

then,

\begin{eqnarray*} \frac{{\mathrm{\v{T} }}_{1}}{\sum _{e=1}^{2}{\mathrm{\v{T} }}_{e}} {\Xi }_{1}{\oplus }_{\mathbb{SS}} \frac{{\mathrm{\v{T} }}_{2}}{\sum _{e=1}^{2}{\mathrm{\v{T} }}_{e}} {\Xi }_{2}= \left( \begin{array}{@{}c@{}} \displaystyle 1-{ \left( \frac{{\mathrm{\v{T} }}_{1}}{\sum _{e=1}^{2}{\mathrm{\v{T} }}_{e}} { \left( 1-{\mathfrak{Y}}_{{\Xi }_{1}}^{RP} \right) }^{\mathrm{\gimel }}- \left( {\mathfrak{K}}_{1}-1 \right) \right) }^{ \frac{1}{\mathrm{\gimel }} }\\ \displaystyle +\iota \left( 1-{ \left( \frac{{\mathrm{\v{T} }}_{1}}{\sum _{e=1}^{2}{\mathrm{\v{T} }}_{e}} { \left( 1-{\mathfrak{Y}}_{{\Xi }_{1}}^{IP} \right) }^{\mathrm{\gimel }}- \left( {\mathfrak{K}}_{1}-1 \right) \right) }^{ \frac{1}{\mathrm{\gimel }} } \right) ,\\ \displaystyle -{ \left( \frac{{\mathrm{\v{T} }}_{1}}{\sum _{e=1}^{2}{\mathrm{\v{T} }}_{e}} { \left\vert {\mathfrak{Y}}_{{\Xi }_{1}}^{RN} \right\vert }^{\mathrm{\gimel }}- \left( {\mathfrak{K}}_{1}-1 \right) \right) }^{ \frac{1}{\mathrm{\gimel }} }\\ \displaystyle +\iota \left( -{ \left( \frac{{\mathrm{\v{T} }}_{1}}{\sum _{e=1}^{2}{\mathrm{\v{T} }}_{e}} { \left\vert {\mathfrak{Y}}_{{\Xi }_{1}}^{IN} \right\vert }^{\mathrm{\gimel }}- \left( {\mathfrak{K}}_{1}-1 \right) \right) }^{ \frac{1}{\mathrm{\gimel }} } \right) \end{array} \right) \nonumber\\\displaystyle {\oplus }_{\mathbb{SS}} \left( \begin{array}{@{}c@{}} \displaystyle 1-{ \left( \frac{{\mathrm{\v{T} }}_{2}}{\sum _{e=1}^{2}{\mathrm{\v{T} }}_{e}} { \left( 1-{\mathfrak{Y}}_{{\Xi }_{2}}^{RP} \right) }^{\mathrm{\gimel }}- \left( {\mathfrak{K}}_{2}-1 \right) \right) }^{ \frac{1}{\mathrm{\gimel }} }\\ \displaystyle +\iota \left( 1-{ \left( \frac{{\mathrm{\v{T} }}_{2}}{\sum _{e=1}^{2}{\mathrm{\v{T} }}_{e}} { \left( 1-{\mathfrak{Y}}_{{\Xi }_{2}}^{IP} \right) }^{\mathrm{\gimel }}- \left( {\mathfrak{K}}_{2}-1 \right) \right) }^{ \frac{1}{\mathrm{\gimel }} } \right) ,\\ \displaystyle -{ \left( \frac{{\mathrm{\v{T} }}_{2}}{\sum _{e=1}^{2}{\mathrm{\v{T} }}_{e}} { \left\vert {\mathfrak{Y}}_{{\Xi }_{2}}^{RN} \right\vert }^{\mathrm{\gimel }}- \left( {\mathfrak{K}}_{2}-1 \right) \right) }^{ \frac{1}{\mathrm{\gimel }} }\\ \displaystyle +\iota \left( -{ \left( \frac{{\mathrm{\v{T} }}_{2}}{\sum _{e=1}^{2}{\mathrm{\v{T} }}_{e}} { \left\vert {\mathfrak{Y}}_{{\Xi }_{2}}^{IN} \right\vert }^{\mathrm{\gimel }}- \left( {\mathfrak{K}}_{2}-1 \right) \right) }^{ \frac{1}{\mathrm{\gimel }} } \right) \end{array} \right) \nonumber\\\displaystyle = \left( \begin{array}{@{}c@{}} \displaystyle \begin{array}{@{}c@{}} \displaystyle \begin{array}{@{}c@{}} \displaystyle 1-{ \left( \begin{array}{@{}c@{}} \displaystyle { \left( 1- \left( 1-{ \left( \frac{{\mathrm{\v{T} }}_{1}}{\sum _{e=1}^{2}{\mathrm{\v{T} }}_{e}} { \left( 1-{\mathfrak{Y}}_{{\Xi }_{1}}^{RP} \right) }^{\mathrm{\gimel }}- \left( \frac{{\mathrm{\v{T} }}_{1}}{\sum _{e=1}^{2}{\mathrm{\v{T} }}_{e}} -1 \right) \right) }^{ \frac{1}{\mathrm{\gimel }} } \right) \right) }^{\mathrm{\gimel }}\\ \displaystyle +{ \left( 1- \left( 1-{ \left( \frac{{\mathrm{\v{T} }}_{2}}{\sum _{e=1}^{2}{\mathrm{\v{T} }}_{e}} { \left( 1-{\mathfrak{Y}}_{{\Xi }_{2}}^{RP} \right) }^{\mathrm{\gimel }}- \left( \frac{{\mathrm{\v{T} }}_{2}}{\sum _{e=1}^{2}{\mathrm{\v{T} }}_{e}} -1 \right) \right) }^{ \frac{1}{\mathrm{\gimel }} } \right) \right) }^{\mathrm{\gimel }}-1 \end{array} \right) }^{ \frac{1}{\mathrm{\gimel }} }\\ \displaystyle +\iota \left( 1-{ \left( \begin{array}{@{}c@{}} \displaystyle { \left( 1- \left( 1-{ \left( \frac{{\mathrm{\v{T} }}_{1}}{\sum _{e=1}^{2}{\mathrm{\v{T} }}_{e}} { \left( 1-{\mathfrak{Y}}_{{\Xi }_{1}}^{IP} \right) }^{\mathrm{\gimel }}- \left( \frac{{\mathrm{\v{T} }}_{1}}{\sum _{e=1}^{2}{\mathrm{\v{T} }}_{e}} -1 \right) \right) }^{ \frac{1}{\mathrm{\gimel }} } \right) \right) }^{\mathrm{\gimel }}\\ \displaystyle +{ \left( 1- \left( 1-{ \left( \frac{{\mathrm{\v{T} }}_{2}}{\sum _{e=1}^{2}{\mathrm{\v{T} }}_{e}} { \left( 1-{\mathfrak{Y}}_{{\Xi }_{2}}^{IP} \right) }^{\mathrm{\gimel }}- \left( \frac{{\mathrm{\v{T} }}_{2}}{\sum _{e=1}^{2}{\mathrm{\v{T} }}_{e}} -1 \right) \right) }^{ \frac{1}{\mathrm{\gimel }} } \right) \right) }^{\mathrm{\gimel }}-1 \end{array} \right) }^{ \frac{1}{\mathrm{\gimel }} } \right) , \end{array}\\ \displaystyle -{ \left( \begin{array}{@{}c@{}} \displaystyle { \left\vert -{ \left( \frac{{\mathrm{\v{T} }}_{1}}{\sum _{e=1}^{2}{\mathrm{\v{T} }}_{e}} { \left\vert {\mathfrak{Y}}_{{\Xi }_{1}}^{RN} \right\vert }^{\mathrm{\gimel }}- \left( \frac{{\mathrm{\v{T} }}_{1}}{\sum _{e=1}^{2}{\mathrm{\v{T} }}_{e}} -1 \right) \right) }^{ \frac{1}{\mathrm{\gimel }} } \right\vert }^{\mathrm{\gimel }}\\ \displaystyle +{ \left\vert -{ \left( \frac{{\mathrm{\v{T} }}_{2}}{\sum _{e=1}^{2}{\mathrm{\v{T} }}_{e}} { \left\vert {\mathfrak{Y}}_{{\Xi }_{2}}^{RN} \right\vert }^{\mathrm{\gimel }}- \left( \frac{{\mathrm{\v{T} }}_{2}}{\sum _{e=1}^{2}{\mathrm{\v{T} }}_{e}} -1 \right) \right) }^{ \frac{1}{\mathrm{\gimel }} } \right\vert }^{\mathrm{\gimel }}-1 \end{array} \right) }^{ \frac{1}{\mathrm{\gimel }} } \end{array}\\ \displaystyle +\iota \left( -{ \left( \begin{array}{@{}c@{}} \displaystyle { \left\vert -{ \left( \frac{{\mathrm{\v{T} }}_{1}}{\sum _{e=1}^{2}{\mathrm{\v{T} }}_{e}} { \left\vert {\mathfrak{Y}}_{{\Xi }_{1}}^{IN} \right\vert }^{\mathrm{\gimel }}- \left( \frac{{\mathrm{\v{T} }}_{1}}{\sum _{e=1}^{2}{\mathrm{\v{T} }}_{e}} -1 \right) \right) }^{ \frac{1}{\mathrm{\gimel }} } \right\vert }^{\mathrm{\gimel }}\\ \displaystyle +{ \left\vert -{ \left( \frac{{\mathrm{\v{T} }}_{2}}{\sum _{e=1}^{2}{\mathrm{\v{T} }}_{e}} { \left\vert {\mathfrak{Y}}_{{\Xi }_{2}}^{IN} \right\vert }^{\mathrm{\gimel }}- \left( \frac{{\mathrm{\v{T} }}_{2}}{\sum _{e=1}^{2}{\mathrm{\v{T} }}_{e}} -1 \right) \right) }^{ \frac{1}{\mathrm{\gimel }} } \right\vert }^{\mathrm{\gimel }}-1 \end{array} \right) }^{ \frac{1}{\mathrm{\gimel }} } \right) \end{array} \right) \end{eqnarray*}

As $ \frac{{\mathrm{\v{T} }}_{1}}{{\mathop{\sum }\nolimits }_{e=1}^{2}{\mathrm{\v{T} }}_{e}} { \left\vert {\mathfrak{Y}}_{{\Xi }_{1}}^{RN} \right\vert }^{\mathrm{\gimel }}- \left( \frac{{\mathrm{\v{T} }}_{1}}{{\mathop{\sum }\nolimits }_{e=1}^{2}{\mathrm{\v{T} }}_{e}} -1 \right) , \frac{{\mathrm{\v{T} }}_{2}}{{\mathop{\sum }\nolimits }_{e=1}^{2}{\mathrm{\v{T} }}_{e}} { \left\vert {\mathfrak{Y}}_{{\Xi }_{2}}^{RN} \right\vert }^{\mathrm{\gimel }}- \left( \frac{{\mathrm{\v{T} }}_{2}}{{\mathop{\sum }\nolimits }_{e=1}^{2}{\mathrm{\v{T} }}_{e}} -1 \right) $, $ \frac{{\mathrm{\v{T} }}_{1}}{{\mathop{\sum }\nolimits }_{e=1}^{2}{\mathrm{\v{T} }}_{e}} { \left\vert {\mathfrak{Y}}_{{\Xi }_{1}}^{IN} \right\vert }^{\mathrm{\gimel }}- \left( \frac{{\mathrm{\v{T} }}_{1}}{{\mathop{\sum }\nolimits }_{e=1}^{2}{\mathrm{\v{T} }}_{e}} -1 \right) $, and $ \frac{{\mathrm{\v{T} }}_{2}}{{\mathop{\sum }\nolimits }_{e=1}^{2}{\mathrm{\v{T} }}_{e}} { \left\vert {\mathfrak{Y}}_{{\Xi }_{2}}^{IN} \right\vert }^{\mathrm{\gimel }}- \left( \frac{{\mathrm{\v{T} }}_{2}}{{\mathop{\sum }\nolimits }_{e=1}^{2}{\mathrm{\v{T} }}_{e}} -1 \right) $ would be always non-negative so $ \left\vert \frac{{\mathrm{\v{T} }}_{1}}{{\mathop{\sum }\nolimits }_{e=1}^{2}{\mathrm{\v{T} }}_{e}} { \left\vert {\mathfrak{Y}}_{{\Xi }_{1}}^{RN} \right\vert }^{\mathrm{\gimel }}- \left( \frac{{\mathrm{\v{T} }}_{1}}{{\mathop{\sum }\nolimits }_{e=1}^{2}{\mathrm{\v{T} }}_{e}} -1 \right) \right\vert = \frac{{\mathrm{\v{T} }}_{1}}{{\mathop{\sum }\nolimits }_{e=1}^{2}{\mathrm{\v{T} }}_{e}} { \left\vert {\mathfrak{Y}}_{{\Xi }_{1}}^{RN} \right\vert }^{\mathrm{\gimel }}- \left( \frac{{\mathrm{\v{T} }}_{1}}{{\mathop{\sum }\nolimits }_{e=1}^{2}{\mathrm{\v{T} }}_{e}} -1 \right) $, $ \left\vert \frac{{\mathrm{\v{T} }}_{2}}{{\mathop{\sum }\nolimits }_{e=1}^{2}{\mathrm{\v{T} }}_{e}} { \left\vert {\mathfrak{Y}}_{{\Xi }_{2}}^{RN} \right\vert }^{\mathrm{\gimel }}- \left( \frac{{\mathrm{\v{T} }}_{2}}{{\mathop{\sum }\nolimits }_{e=1}^{2}{\mathrm{\v{T} }}_{e}} -1 \right) \right\vert = \frac{{\mathrm{\v{T} }}_{2}}{{\mathop{\sum }\nolimits }_{e=1}^{2}{\mathrm{\v{T} }}_{e}} { \left\vert {\mathfrak{Y}}_{{\Xi }_{2}}^{RN} \right\vert }^{\mathrm{\gimel }}- \left( \frac{{\mathrm{\v{T} }}_{2}}{{\mathop{\sum }\nolimits }_{e=1}^{2}{\mathrm{\v{T} }}_{e}} -1 \right) $, $ \left\vert \frac{{\mathrm{\v{T} }}_{1}}{{\mathop{\sum }\nolimits }_{e=1}^{2}{\mathrm{\v{T} }}_{e}} { \left\vert {\mathfrak{Y}}_{{\Xi }_{1}}^{IN} \right\vert }^{\mathrm{\gimel }}- \left( \frac{{\mathrm{\v{T} }}_{1}}{{\mathop{\sum }\nolimits }_{e=1}^{2}{\mathrm{\v{T} }}_{e}} -1 \right) \right\vert = \frac{{\mathrm{\v{T} }}_{1}}{{\mathop{\sum }\nolimits }_{e=1}^{2}{\mathrm{\v{T} }}_{e}} { \left\vert {\mathfrak{Y}}_{{\Xi }_{1}}^{IN} \right\vert }^{\mathrm{\gimel }}- \left( \frac{{\mathrm{\v{T} }}_{1}}{{\mathop{\sum }\nolimits }_{e=1}^{2}{\mathrm{\v{T} }}_{e}} -1 \right) $, $ \left\vert \frac{{\mathrm{\v{T} }}_{2}}{{\mathop{\sum }\nolimits }_{e=1}^{2}{\mathrm{\v{T} }}_{e}} { \left\vert {\mathfrak{Y}}_{{\Xi }_{2}}^{IN} \right\vert }^{\mathrm{\gimel }}- \left( \frac{{\mathrm{\v{T} }}_{2}}{{\mathop{\sum }\nolimits }_{e=1}^{2}{\mathrm{\v{T} }}_{e}} -1 \right) \right\vert = \frac{{\mathrm{\v{T} }}_{2}}{{\mathop{\sum }\nolimits }_{e=1}^{2}{\mathrm{\v{T} }}_{e}} { \left\vert {\mathfrak{Y}}_{{\Xi }_{2}}^{IN} \right\vert }^{\mathrm{\gimel }}- \left( \frac{{\mathrm{\v{T} }}_{2}}{{\mathop{\sum }\nolimits }_{e=1}^{2}{\mathrm{\v{T} }}_{e}} -1 \right) $.

\begin{eqnarray*}= \left( \begin{array}{@{}c@{}} \displaystyle \begin{array}{@{}c@{}} \displaystyle \begin{array}{@{}c@{}} \displaystyle 1-{ \left( \begin{array}{@{}c@{}} \displaystyle \frac{{\mathrm{\v{T} }}_{1}}{\sum _{e=1}^{2}{\mathrm{\v{T} }}_{e}} { \left( 1-{\mathfrak{Y}}_{{\Xi }_{1}}^{RP} \right) }^{\mathrm{\gimel }}- \left( \frac{{\mathrm{\v{T} }}_{1}}{\sum _{e=1}^{2}{\mathrm{\v{T} }}_{e}} -1 \right) \vskip{1pc}\\ \displaystyle + \frac{{\mathrm{\v{T} }}_{2}}{\sum _{e=1}^{2}{\mathrm{\v{T} }}_{e}} { \left( 1-{\mathfrak{Y}}_{{\Xi }_{2}}^{RP} \right) }^{\mathrm{\gimel }}- \left( {\mathfrak{K}}_{2}-1 \right) -1 \end{array} \right) }^{ \frac{1}{\mathrm{\gimel }} }\\ \displaystyle +\iota \left( 1-{ \left( \begin{array}{@{}c@{}} \displaystyle \frac{{\mathrm{\v{T} }}_{1}}{\sum _{e=1}^{2}{\mathrm{\v{T} }}_{e}} { \left( 1-{\mathfrak{Y}}_{{\Xi }_{1}}^{IP} \right) }^{\mathrm{\gimel }}- \left( \frac{{\mathrm{\v{T} }}_{1}}{\sum _{e=1}^{2}{\mathrm{\v{T} }}_{e}} -1 \right) \vskip{1pc}\\ \displaystyle + \frac{{\mathrm{\v{T} }}_{2}}{\sum _{e=1}^{2}{\mathrm{\v{T} }}_{e}} { \left( 1-{\mathfrak{Y}}_{{\Xi }_{2}}^{IP} \right) }^{\mathrm{\gimel }}- \left( {\mathfrak{K}}_{2}-1 \right) -1 \end{array} \right) }^{ \frac{1}{\mathrm{\gimel }} } \right) , \end{array}\\ \displaystyle -{ \left( \frac{{\mathrm{\v{T} }}_{1}}{\sum _{e=1}^{2}{\mathrm{\v{T} }}_{e}} { \left\vert {\mathfrak{Y}}_{{\Xi }_{1}}^{RN} \right\vert }^{\mathrm{\gimel }}- \left( \frac{{\mathrm{\v{T} }}_{1}}{\sum _{e=1}^{2}{\mathrm{\v{T} }}_{e}} -1 \right) + \frac{{\mathrm{\v{T} }}_{2}}{\sum _{e=1}^{2}{\mathrm{\v{T} }}_{e}} { \left\vert {\mathfrak{Y}}_{{\Xi }_{2}}^{RN} \right\vert }^{\mathrm{\gimel }}- \left( {\mathfrak{K}}_{2}-1 \right) -1 \right) }^{ \frac{1}{\mathrm{\gimel }} } \end{array}\\ \displaystyle +\iota \left( -{ \left( \frac{{\mathrm{\v{T} }}_{1}}{\sum _{e=1}^{2}{\mathrm{\v{T} }}_{e}} { \left\vert {\mathfrak{Y}}_{{\Xi }_{1}}^{IN} \right\vert }^{\mathrm{\gimel }}- \left( \frac{{\mathrm{\v{T} }}_{1}}{\sum _{e=1}^{2}{\mathrm{\v{T} }}_{e}} -1 \right) + \frac{{\mathrm{\v{T} }}_{2}}{\sum _{e=1}^{2}{\mathrm{\v{T} }}_{e}} { \left\vert {\mathfrak{Y}}_{{\Xi }_{2}}^{IN} \right\vert }^{\mathrm{\gimel }}- \left( {\mathfrak{K}}_{2}-1 \right) -1 \right) }^{ \frac{1}{\mathrm{\gimel }} } \right) \end{array} \right) \nonumber\\\displaystyle = \left( \begin{array}{@{}c@{}} \displaystyle 1-{ \left( \sum _{e=1}^{2} \frac{{\mathrm{\v{T} }}_{e}}{\sum _{e=1}^{2}{\mathrm{\v{T} }}_{e}} { \left( 1-{\mathfrak{Y}}_{{\Xi }_{e}}^{RP} \right) }^{\mathrm{\gimel }}-\sum _{e=1}^{2} \frac{{\mathrm{\v{T} }}_{e}}{\sum _{e=1}^{2}{\mathrm{\v{T} }}_{e}} +1 \right) }^{ \frac{1}{\mathrm{\gimel }} }\\ \displaystyle +\iota \left( 1-{ \left( \sum _{e=1}^{2} \frac{{\mathrm{\v{T} }}_{e}}{\sum _{e=1}^{2}{\mathrm{\v{T} }}_{e}} { \left( 1-{\mathfrak{Y}}_{{\Xi }_{e}}^{IP} \right) }^{\mathrm{\gimel }}-\sum _{e=1}^{2} \frac{{\mathrm{\v{T} }}_{e}}{\sum _{e=1}^{2}{\mathrm{\v{T} }}_{e}} +1 \right) }^{ \frac{1}{\mathrm{\gimel }} } \right) ,\\ \displaystyle -{ \left( \sum _{e=1}^{2} \frac{{\mathrm{\v{T} }}_{e}}{\sum _{e=1}^{2}{\mathrm{\v{T} }}_{e}} { \left\vert {\mathfrak{Y}}_{{\Xi }_{e}}^{RN} \right\vert }^{\mathrm{\gimel }}-\sum _{e=1}^{2} \frac{{\mathrm{\v{T} }}_{e}}{\sum _{e=1}^{2}{\mathrm{\v{T} }}_{e}} +1 \right) }^{ \frac{1}{\mathrm{\gimel }} }\\ \displaystyle +\iota \left( -{ \left( \sum _{e=1}^{2}{\mathfrak{K}}_{e}{ \left\vert {\mathfrak{Y}}_{{\Xi }_{e}}^{IN} \right\vert }^{\mathrm{\gimel }} \right) }^{ \frac{1}{\mathrm{\gimel }} }-\sum _{e=1}^{2} \frac{{\mathrm{\v{T} }}_{e}}{\sum _{e=1}^{2}{\mathrm{\v{T} }}_{e}} +1 \right) \end{array} \right) \end{eqnarray*}

Thus, Eq. (9) is valid for $\check {\mathrm{k}}=2$. Take Eq. (9) is additionally valid for $\check {\mathrm{k}}=\Gamma $, then

\begin{eqnarray*}BCFSSPRWA \left( {\Xi }_{1},{\Xi }_{2},\ldots ,{\Xi }_{\Gamma } \right) \nonumber\\\displaystyle = \left( \begin{array}{@{}c@{}} \displaystyle 1-{ \left( \sum _{e=1}^{\Gamma } \frac{{\mathrm{\v{T} }}_{e}}{\sum _{e=1}^{\Gamma }{\mathrm{\v{T} }}_{e}} { \left( 1-{\mathfrak{Y}}_{{\Xi }_{e}}^{RP} \right) }^{\mathrm{\gimel }}-\sum _{e=1}^{\Gamma } \frac{{\mathrm{\v{T} }}_{e}}{\sum _{e=1}^{\Gamma }{\mathrm{\v{T} }}_{e}} +1 \right) }^{ \frac{1}{\mathrm{\gimel }} }\\ \displaystyle +\iota \left( 1-{ \left( \sum _{e=1}^{\Gamma } \frac{{\mathrm{\v{T} }}_{e}}{\sum _{e=1}^{\Gamma }{\mathrm{\v{T} }}_{e}} { \left( 1-{\mathfrak{Y}}_{{\Xi }_{e}}^{IP} \right) }^{\mathrm{\gimel }}-\sum _{e=1}^{\Gamma } \frac{{\mathrm{\v{T} }}_{e}}{\sum _{e=1}^{\Gamma }{\mathrm{\v{T} }}_{e}} +1 \right) }^{ \frac{1}{\mathrm{\gimel }} } \right) ,\\ \displaystyle -{ \left( \sum _{e=1}^{\Gamma } \frac{{\mathrm{\v{T} }}_{e}}{\sum _{e=1}^{\Gamma }{\mathrm{\v{T} }}_{e}} { \left\vert {\mathfrak{Y}}_{{\Xi }_{e}}^{RN} \right\vert }^{\mathrm{\gimel }}-\sum _{e=1}^{\Gamma } \frac{{\mathrm{\v{T} }}_{e}}{\sum _{e=1}^{\Gamma }{\mathrm{\v{T} }}_{e}} +1 \right) }^{ \frac{1}{\mathrm{\gimel }} }\\ \displaystyle +\iota \left( -{ \left( \sum _{e=1}^{\Gamma } \frac{{\mathrm{\v{T} }}_{e}}{\sum _{e=1}^{\Gamma }{\mathrm{\v{T} }}_{e}} { \left\vert {\mathfrak{Y}}_{{\Xi }_{e}}^{IN} \right\vert }^{\mathrm{\gimel }} \right) }^{ \frac{1}{\mathrm{\gimel }} }-\sum _{e=1}^{\Gamma } \frac{{\mathrm{\v{T} }}_{e}}{\sum _{e=1}^{\Gamma }{\mathrm{\v{T} }}_{e}} +1 \right) \end{array} \right) \end{eqnarray*}

Next take $\check {\mathrm{k}}=\Gamma +1$, then

\begin{eqnarray*}BCFSSPRWA \left( {\Xi }_{1},{\Xi }_{2},\ldots ,{\Xi }_{\Gamma },{\Xi }_{\Gamma +1} \right) =BCFSSPRWA \left( {\Xi }_{1},{\Xi }_{2},\ldots ,{\Xi }_{\Gamma } \right) {\oplus }_{\mathbb{SS}}{\Xi }_{\Gamma +1}\nonumber\\\displaystyle = \left( \begin{array}{@{}c@{}} \displaystyle 1-{ \left( \sum _{e=1}^{\Gamma } \frac{{\mathrm{\v{T} }}_{e}}{\sum _{e=1}^{\Gamma }{\mathrm{\v{T} }}_{e}} { \left( 1-{\mathfrak{Y}}_{{\Xi }_{e}}^{RP} \right) }^{\mathrm{\gimel }}-\sum _{e=1}^{\Gamma } \frac{{\mathrm{\v{T} }}_{e}}{\sum _{e=1}^{\Gamma }{\mathrm{\v{T} }}_{e}} +1 \right) }^{ \frac{1}{\mathrm{\gimel }} }\\ \displaystyle +\iota \left( 1-{ \left( \sum _{e=1}^{\Gamma } \frac{{\mathrm{\v{T} }}_{e}}{\sum _{e=1}^{\Gamma }{\mathrm{\v{T} }}_{e}} { \left( 1-{\mathfrak{Y}}_{{\Xi }_{e}}^{IP} \right) }^{\mathrm{\gimel }}-\sum _{e=1}^{\Gamma } \frac{{\mathrm{\v{T} }}_{e}}{\sum _{e=1}^{\Gamma }{\mathrm{\v{T} }}_{e}} +1 \right) }^{ \frac{1}{\mathrm{\gimel }} } \right) ,\\ \displaystyle -{ \left( \sum _{e=1}^{\Gamma } \frac{{\mathrm{\v{T} }}_{e}}{\sum _{e=1}^{\Gamma }{\mathrm{\v{T} }}_{e}} { \left\vert {\mathfrak{Y}}_{{\Xi }_{e}}^{RN} \right\vert }^{\mathrm{\gimel }}-\sum _{e=1}^{\Gamma } \frac{{\mathrm{\v{T} }}_{e}}{\sum _{e=1}^{\Gamma }{\mathrm{\v{T} }}_{e}} +1 \right) }^{ \frac{1}{\mathrm{\gimel }} }\\ \displaystyle +\iota \left( -{ \left( \sum _{e=1}^{\Gamma } \frac{{\mathrm{\v{T} }}_{e}}{\sum _{e=1}^{\Gamma }{\mathrm{\v{T} }}_{e}} { \left\vert {\mathfrak{Y}}_{{\Xi }_{e}}^{IN} \right\vert }^{\mathrm{\gimel }} \right) }^{ \frac{1}{\mathrm{\gimel }} }-\sum _{e=1}^{\Gamma } \frac{{\mathrm{\v{T} }}_{e}}{\sum _{e=1}^{\Gamma }{\mathrm{\v{T} }}_{e}} +1 \right) \end{array} \right) \nonumber\\\displaystyle {\oplus }_{\mathbb{SS}} \left( \begin{array}{@{}c@{}} \displaystyle 1-{ \left( \frac{{\mathrm{\v{T} }}_{\Gamma +1}}{\sum _{e=1}^{\Gamma +1}{\mathrm{\v{T} }}_{e}} { \left( 1-{\mathfrak{Y}}_{{\Xi }_{\Gamma +1}}^{RP} \right) }^{\mathrm{\gimel }}- \left( {\mathfrak{K}}_{\Gamma +1}-1 \right) \right) }^{ \frac{1}{\mathrm{\gimel }} }\\ \displaystyle +\iota \left( 1-{ \left( \frac{{\mathrm{\v{T} }}_{\Gamma +1}}{\sum _{e=1}^{\Gamma +1}{\mathrm{\v{T} }}_{e}} { \left( 1-{\mathfrak{Y}}_{{\Xi }_{\Gamma +1}}^{IP} \right) }^{\mathrm{\gimel }}- \left( {\mathfrak{K}}_{\Gamma +1}-1 \right) \right) }^{ \frac{1}{\mathrm{\gimel }} } \right) ,\\ \displaystyle -{ \left( \frac{{\mathrm{\v{T} }}_{\Gamma +1}}{\sum _{e=1}^{\Gamma +1}{\mathrm{\v{T} }}_{e}} { \left\vert {\mathfrak{Y}}_{{\Xi }_{\Gamma +1}}^{RN} \right\vert }^{\mathrm{\gimel }}- \left( {\mathfrak{K}}_{\Gamma +1}-1 \right) \right) }^{ \frac{1}{\mathrm{\gimel }} }\\ \displaystyle +\iota \left( -{ \left( \frac{{\mathrm{\v{T} }}_{\Gamma +1}}{\sum _{e=1}^{\Gamma +1}{\mathrm{\v{T} }}_{e}} { \left\vert {\mathfrak{Y}}_{{\Xi }_{\Gamma +1}}^{IN} \right\vert }^{\mathrm{\gimel }}- \left( {\mathfrak{K}}_{\Gamma +1}-1 \right) \right) }^{ \frac{1}{\mathrm{\gimel }} } \right) \end{array} \right) \nonumber\\\displaystyle = \left( \begin{array}{@{}c@{}} \displaystyle \begin{array}{@{}c@{}} \displaystyle \begin{array}{@{}c@{}} \displaystyle 1-{ \left( \begin{array}{@{}c@{}} \displaystyle { \left( 1- \left( 1-{ \left( \sum _{e=1}^{\Gamma } \frac{{\mathrm{\v{T} }}_{e}}{\sum _{e=1}^{\Gamma }{\mathrm{\v{T} }}_{e}} { \left( 1-{\mathfrak{Y}}_{{\Xi }_{e}}^{RP} \right) }^{\mathrm{\gimel }}-\sum _{e=1}^{\Gamma } \frac{{\mathrm{\v{T} }}_{e}}{\sum _{e=1}^{\Gamma }{\mathrm{\v{T} }}_{e}} +1 \right) }^{ \frac{1}{\mathrm{\gimel }} } \right) \right) }^{\mathrm{\gimel }}\\ \displaystyle +{ \left( 1- \left( 1-{ \left( \frac{{\mathrm{\v{T} }}_{\Gamma +1}}{\sum _{e=1}^{\Gamma +1}{\mathrm{\v{T} }}_{e}} { \left( 1-{\mathfrak{Y}}_{{\Xi }_{\Gamma +1}}^{RP} \right) }^{\mathrm{\gimel }}- \left( \frac{{\mathrm{\v{T} }}_{\Gamma +1}}{\sum _{e=1}^{\Gamma +1}{\mathrm{\v{T} }}_{e}} -1 \right) \right) }^{ \frac{1}{\mathrm{\gimel }} } \right) \right) }^{\mathrm{\gimel }}-1 \end{array} \right) }^{ \frac{1}{\mathrm{\gimel }} }\\ \displaystyle +\iota \left( 1-{ \left( \begin{array}{@{}c@{}} \displaystyle { \left( 1- \left( 1-{ \left( \sum _{e=1}^{\Gamma } \frac{{\mathrm{\v{T} }}_{e}}{\sum _{e=1}^{\Gamma }{\mathrm{\v{T} }}_{e}} { \left( 1-{\mathfrak{Y}}_{{\Xi }_{e}}^{IP} \right) }^{\mathrm{\gimel }}-\sum _{e=1}^{\Gamma } \frac{{\mathrm{\v{T} }}_{e}}{\sum _{e=1}^{\Gamma }{\mathrm{\v{T} }}_{e}} +1 \right) }^{ \frac{1}{\mathrm{\gimel }} } \right) \right) }^{\mathrm{\gimel }}\\ \displaystyle +{ \left( 1- \left( 1-{ \left( \frac{{\mathrm{\v{T} }}_{\Gamma +1}}{\sum _{e=1}^{\Gamma +1}{\mathrm{\v{T} }}_{e}} { \left( 1-{\mathfrak{Y}}_{{\Xi }_{\Gamma +1}}^{IP} \right) }^{\mathrm{\gimel }}- \left( \frac{{\mathrm{\v{T} }}_{\Gamma +1}}{\sum _{e=1}^{\Gamma +1}{\mathrm{\v{T} }}_{e}} -1 \right) \right) }^{ \frac{1}{\mathrm{\gimel }} } \right) \right) }^{\mathrm{\gimel }}-1 \end{array} \right) }^{ \frac{1}{\mathrm{\gimel }} } \right) , \end{array}\\ \displaystyle -{ \left( \begin{array}{@{}c@{}} \displaystyle { \left\vert -{ \left( \sum _{e=1}^{\Gamma } \frac{{\mathrm{\v{T} }}_{e}}{\sum _{e=1}^{\Gamma }{\mathrm{\v{T} }}_{e}} { \left\vert {\mathfrak{Y}}_{{\Xi }_{e}}^{RN} \right\vert }^{\mathrm{\gimel }}-\sum _{e=1}^{\Gamma } \frac{{\mathrm{\v{T} }}_{e}}{\sum _{e=1}^{\Gamma }{\mathrm{\v{T} }}_{e}} +1 \right) }^{ \frac{1}{\mathrm{\gimel }} } \right\vert }^{\mathrm{\gimel }}\\ \displaystyle +{ \left\vert -{ \left( \frac{{\mathrm{\v{T} }}_{\Gamma +1}}{\sum _{e=1}^{\Gamma +1}{\mathrm{\v{T} }}_{e}} { \left\vert {\mathfrak{Y}}_{{\Xi }_{\Gamma +1}}^{RN} \right\vert }^{\mathrm{\gimel }}- \left( \frac{{\mathrm{\v{T} }}_{\Gamma +1}}{\sum _{e=1}^{\Gamma +1}{\mathrm{\v{T} }}_{e}} -1 \right) \right) }^{ \frac{1}{\mathrm{\gimel }} } \right\vert }^{\mathrm{\gimel }}-1 \end{array} \right) }^{ \frac{1}{\mathrm{\gimel }} } \end{array}\\ \displaystyle +\iota \left( -{ \left( \begin{array}{@{}c@{}} \displaystyle { \left\vert -{ \left( \sum _{e=1}^{\Gamma } \frac{{\mathrm{\v{T} }}_{e}}{\sum _{e=1}^{\Gamma }{\mathrm{\v{T} }}_{e}} { \left\vert {\mathfrak{Y}}_{{\Xi }_{e}}^{IN} \right\vert }^{\mathrm{\gimel }}-\sum _{e=1}^{\Gamma } \frac{{\mathrm{\v{T} }}_{e}}{\sum _{e=1}^{\Gamma }{\mathrm{\v{T} }}_{e}} +1 \right) }^{ \frac{1}{\mathrm{\gimel }} } \right\vert }^{\mathrm{\gimel }}\\ \displaystyle +{ \left\vert -{ \left( \frac{{\mathrm{\v{T} }}_{\Gamma +1}}{\sum _{e=1}^{\Gamma +1}{\mathrm{\v{T} }}_{e}} { \left\vert {\mathfrak{Y}}_{{\Xi }_{\Gamma +1}}^{IN} \right\vert }^{\mathrm{\gimel }}- \left( \frac{{\mathrm{\v{T} }}_{\Gamma +1}}{\sum _{e=1}^{\Gamma +1}{\mathrm{\v{T} }}_{e}} -1 \right) \right) }^{ \frac{1}{\mathrm{\gimel }} } \right\vert }^{\mathrm{\gimel }}-1 \end{array} \right) }^{ \frac{1}{\mathrm{\gimel }} } \right) \end{array} \right) \nonumber\\\displaystyle = \left( \begin{array}{@{}c@{}} \displaystyle 1-{ \left( \sum _{e=1}^{\Gamma +1} \frac{{\mathrm{\v{T} }}_{e}}{\sum _{e=1}^{\Gamma }{\mathrm{\v{T} }}_{e}} { \left( 1-{\mathfrak{Y}}_{{\Xi }_{e}}^{RP} \right) }^{\mathrm{\gimel }}-\sum _{e=1}^{\Gamma +1} \frac{{\mathrm{\v{T} }}_{e}}{\sum _{e=1}^{\Gamma }{\mathrm{\v{T} }}_{e}} +1 \right) }^{ \frac{1}{\mathrm{\gimel }} }\\ \displaystyle +\iota \left( 1-{ \left( \sum _{e=1}^{\Gamma +1} \frac{{\mathrm{\v{T} }}_{e}}{\sum _{e=1}^{\Gamma }{\mathrm{\v{T} }}_{e}} { \left( 1-{\mathfrak{Y}}_{{\Xi }_{e}}^{IP} \right) }^{\mathrm{\gimel }}-\sum _{e=1}^{\Gamma +1} \frac{{\mathrm{\v{T} }}_{e}}{\sum _{e=1}^{\Gamma }{\mathrm{\v{T} }}_{e}} +1 \right) }^{ \frac{1}{\mathrm{\gimel }} } \right) ,\\ \displaystyle -{ \left( \sum _{e=1}^{\Gamma +1} \frac{{\mathrm{\v{T} }}_{e}}{\sum _{e=1}^{\Gamma }{\mathrm{\v{T} }}_{e}} { \left\vert {\mathfrak{Y}}_{{\Xi }_{e}}^{RN} \right\vert }^{\mathrm{\gimel }}-\sum _{e=1}^{\Gamma +1} \frac{{\mathrm{\v{T} }}_{e}}{\sum _{e=1}^{\Gamma }{\mathrm{\v{T} }}_{e}} +1 \right) }^{ \frac{1}{\mathrm{\gimel }} }\\ \displaystyle +\iota \left( -{ \left( \sum _{e=1}^{\Gamma +1} \frac{{\mathrm{\v{T} }}_{e}}{\sum _{e=1}^{\Gamma }{\mathrm{\v{T} }}_{e}} { \left\vert {\mathfrak{Y}}_{{\Xi }_{e}}^{IN} \right\vert }^{\mathrm{\gimel }} \right) }^{ \frac{1}{\mathrm{\gimel }} }-\sum _{e=1}^{\Gamma +1} \frac{{\mathrm{\v{T} }}_{e}}{\sum _{e=1}^{\Gamma }{\mathrm{\v{T} }}_{e}} +1 \right) \end{array} \right) \end{eqnarray*}

Thus, the Eq. (9) is valid for $\check {\mathrm{k}}=\Gamma +1$, this implies that Eq. (9) is valid $\forall \check {\mathrm{k}}$.

**Axiom 1: (Idempotency)** Consider ${\Xi }_{e}= \left( {\mathfrak{Y}}_{{\Xi }_{e}}^{P},{\mathfrak{Y}}_{{\Xi }_{e}}^{N} \right) = \left( {\mathfrak{Y}}_{{\Xi }_{e}}^{RP}+\iota {\mathfrak{Y}}_{{\Xi }_{e}}^{IP},{\mathfrak{Y}}_{{\Xi }_{e}}^{RN}+\iota {\mathfrak{Y}}_{{\Xi }_{e}}^{IN} \right) ,e=1,2,..,\check {\mathrm{k}}$ as a group of BCFNs, and if ${\Xi }_{e}=\Xi = \left( {\mathfrak{Y}}_{\Xi }^{P},{\mathfrak{Y}}_{\Xi }^{N} \right) = \left( {\mathfrak{Y}}_{\Xi }^{RP}+\iota {\mathfrak{Y}}_{\Xi }^{IP},{\mathfrak{Y}}_{\Xi }^{RN}+\iota {\mathfrak{Y}}_{\Xi }^{IN} \right) \forall e$, then

\begin{eqnarray*}BCFSSPRWA \left( {\Xi }_{1},{\Xi }_{2},\ldots ,{\Xi }_{\check {\mathrm{k}}} \right) =\Xi \end{eqnarray*}

**Proof:**

\begin{eqnarray*}BCFSSPRWA \left( {\Xi }_{1},{\Xi }_{2},\ldots ,{\Xi }_{\check {\mathrm{k}}} \right) \nonumber\\\displaystyle = \left( \begin{array}{@{}c@{}} \displaystyle 1-{ \left( \sum _{e=1}^{\check {\mathrm{k}}} \frac{{\mathrm{\v{T} }}_{e}}{\sum _{e=1}^{\check {\mathrm{k}}}{\mathrm{\v{T} }}_{e}} { \left( 1-{\mathfrak{Y}}_{{\Xi }_{e}}^{RP} \right) }^{\mathrm{\gimel }} \right) }^{ \frac{1}{\mathrm{\gimel }} }+\iota \left( 1-{ \left( \sum _{e=1}^{\check {\mathrm{k}}} \frac{{\mathrm{\v{T} }}_{e}}{\sum _{e=1}^{\check {\mathrm{k}}}{\mathrm{\v{T} }}_{e}} { \left( 1-{\mathfrak{Y}}_{{\Xi }_{e}}^{IP} \right) }^{\mathrm{\gimel }} \right) }^{ \frac{1}{\mathrm{\gimel }} } \right) ,\\ \displaystyle -{ \left( \sum _{e=1}^{\check {\mathrm{k}}} \frac{{\mathrm{\v{T} }}_{e}}{\sum _{e=1}^{\check {\mathrm{k}}}{\mathrm{\v{T} }}_{e}} { \left\vert {\mathfrak{Y}}_{{\Xi }_{e}}^{RN} \right\vert }^{\mathrm{\gimel }} \right) }^{ \frac{1}{\mathrm{\gimel }} }+\iota \left( -{ \left( \sum _{e=1}^{\check {\mathrm{k}}} \frac{{\mathrm{\v{T} }}_{e}}{\sum _{e=1}^{\check {\mathrm{k}}}{\mathrm{\v{T} }}_{e}} { \left\vert {\mathfrak{Y}}_{{\Xi }_{e}}^{IN} \right\vert }^{\mathrm{\gimel }} \right) }^{ \frac{1}{\mathrm{\gimel }} } \right) \end{array} \right) \end{eqnarray*}

\begin{eqnarray*}= \left( \begin{array}{@{}c@{}} \displaystyle 1-{ \left( \sum _{e=1}^{\check {\mathrm{k}}} \frac{{\mathrm{\v{T} }}_{e}}{\sum _{e=1}^{\check {\mathrm{k}}}{\mathrm{\v{T} }}_{e}} { \left( 1-{\mathfrak{Y}}_{\Xi }^{RP} \right) }^{\mathrm{\gimel }} \right) }^{ \frac{1}{\mathrm{\gimel }} }+\iota \left( 1-{ \left( \sum _{e=1}^{\check {\mathrm{k}}} \frac{{\mathrm{\v{T} }}_{e}}{\sum _{e=1}^{\check {\mathrm{k}}}{\mathrm{\v{T} }}_{e}} { \left( 1-{\mathfrak{Y}}_{\Xi }^{IP} \right) }^{\mathrm{\gimel }} \right) }^{ \frac{1}{\mathrm{\gimel }} } \right) ,\\ \displaystyle -{ \left( \sum _{e=1}^{\check {\mathrm{k}}} \frac{{\mathrm{\v{T} }}_{e}}{\sum _{e=1}^{\check {\mathrm{k}}}{\mathrm{\v{T} }}_{e}} { \left\vert {\mathfrak{Y}}_{\Xi }^{RN} \right\vert }^{\mathrm{\gimel }} \right) }^{ \frac{1}{\mathrm{\gimel }} }+\iota \left( -{ \left( \sum _{e=1}^{\check {\mathrm{k}}} \frac{{\mathrm{\v{T} }}_{e}}{\sum _{e=1}^{\check {\mathrm{k}}}{\mathrm{\v{T} }}_{e}} { \left\vert {\mathfrak{Y}}_{\Xi }^{IN} \right\vert }^{\mathrm{\gimel }} \right) }^{ \frac{1}{\mathrm{\gimel }} } \right) \end{array} \right) \end{eqnarray*}

As ${\mathfrak{Y}}_{\Xi }^{RN}\in \left[ -1,0 \right] $, then $ \left\vert {\mathfrak{Y}}_{\Xi }^{RN} \right\vert =-{\mathfrak{Y}}_{\Xi }^{RN}$ and also ${\mathfrak{Y}}_{\Xi }^{IN}\in \left[ -1,0 \right] $, then $ \left\vert {\mathfrak{Y}}_{\Xi }^{IN} \right\vert =-{\mathfrak{Y}}_{\Xi }^{IN}$. This implies that

\begin{eqnarray*}BCFSSPRWA \left( {\Xi }_{1},{\Xi }_{2},\ldots ,{\Xi }_{\check {\mathrm{k}}} \right) = \left( {\mathfrak{Y}}_{\Xi }^{RP}+\iota {\mathfrak{Y}}_{\Xi }^{IP},{\mathfrak{Y}}_{\Xi }^{RN}+\iota {\mathfrak{Y}}_{\Xi }^{IN} \right) = \left( {\mathfrak{Y}}_{\Xi }^{P},{\mathfrak{Y}}_{\Xi }^{N} \right) =\Xi . \end{eqnarray*}

**Axiom 2: (Monotonicity)** Consider ${\Xi }_{e}= \left( {\mathfrak{Y}}_{{\Xi }_{e}}^{P},{\mathfrak{Y}}_{{\Xi }_{e}}^{N} \right) = \left( {\mathfrak{Y}}_{{\Xi }_{e}}^{RP}+\iota {\mathfrak{Y}}_{{\Xi }_{e}}^{IP},{\mathfrak{Y}}_{{\Xi }_{e}}^{RN}+\iota {\mathfrak{Y}}_{{\Xi }_{e}}^{IN} \right) ,$ and ${\Xi }_{e}^{{}^{{^{\prime}}}}= \left( {\mathfrak{Y}}_{{\Xi }_{BCFS-e}^{{}^{{^{\prime}}}}}^{P},{\mathfrak{Y}}_{{\Xi }_{e}^{{}^{{^{\prime}}}}}^{N} \right) = \left( {\mathfrak{Y}}_{{\Xi }_{e}^{{}^{{^{\prime}}}}}^{RP}+\iota {\mathfrak{Y}}_{{\Xi }_{e}^{{}^{{^{\prime}}}}}^{IP},{\mathfrak{Y}}_{{\Xi }_{e}^{{}^{{^{\prime}}}}}^{RN}+\iota {\mathfrak{Y}}_{{\Xi }_{e}^{{}^{{^{\prime}}}}}^{IN} \right) ,e=1,2,...,\check {\mathrm{k}}$ astwo groups of BCFNs, and if ${\mathfrak{Y}}_{{\Xi }_{e}}^{RP}\leq {\mathfrak{Y}}_{{\Xi }_{e}^{{}^{{^{\prime}}}}}^{RP}$, ${\mathfrak{Y}}_{{\Xi }_{e}}^{IP}\leq {\mathfrak{Y}}_{{\Xi }_{e}^{{}^{{^{\prime}}}}}^{IP}$, ${\mathfrak{Y}}_{{\Xi }_{e}}^{RN}\leq {\mathfrak{Y}}_{{\Xi }_{e}^{{}^{{^{\prime}}}}}^{RN}$, and ${\mathfrak{Y}}_{{\Xi }_{e}}^{IN}\leq {\mathfrak{Y}}_{{\Xi }_{e}^{{}^{{^{\prime}}}}}^{IN}$ , then

\begin{eqnarray*}BCFSSPRWA \left( {\Xi }_{1},{\Xi }_{2},\ldots ,{\Xi }_{\check {\mathrm{k}}} \right) \leq BCFSSPRWA \left( {\Xi }_{1}^{{}^{{^{\prime}}}},{\Xi }_{2}^{{}^{{^{\prime}}}},\ldots ,{\Xi }_{\check {\mathrm{ k}}}^{{}^{{^{\prime}}}} \right) \end{eqnarray*}

**Proof**

\begin{eqnarray*}BCFSSPRWA \left( {\Xi }_{1},{\Xi }_{2},\ldots ,{\Xi }_{\check {\mathrm{k}}} \right) \nonumber\\\displaystyle = \left( \begin{array}{@{}c@{}} \displaystyle 1-{ \left( \sum _{e=1}^{\check {\mathrm{k}}} \frac{{\mathrm{\v{T} }}_{e}}{\sum _{e=1}^{\check {\mathrm{k}}}{\mathrm{\v{T} }}_{e}} { \left( 1-{\mathfrak{Y}}_{{\Xi }_{e}}^{RP} \right) }^{\mathrm{\gimel }} \right) }^{ \frac{1}{\mathrm{\gimel }} }+\iota \left( 1-{ \left( \sum _{e=1}^{\check {\mathrm{k}}} \frac{{\mathrm{\v{T} }}_{e}}{\sum _{e=1}^{\check {\mathrm{k}}}{\mathrm{\v{T} }}_{e}} { \left( 1-{\mathfrak{Y}}_{{\Xi }_{e}}^{IP} \right) }^{\mathrm{\gimel }} \right) }^{ \frac{1}{\mathrm{\gimel }} } \right) ,\\ \displaystyle -{ \left( \sum _{e=1}^{\check {\mathrm{k}}} \frac{{\mathrm{\v{T} }}_{e}}{\sum _{e=1}^{\check {\mathrm{k}}}{\mathrm{\v{T} }}_{e}} { \left\vert {\mathfrak{Y}}_{{\Xi }_{e}}^{RN} \right\vert }^{\mathrm{\gimel }} \right) }^{ \frac{1}{\mathrm{\gimel }} }+\iota \left( -{ \left( \sum _{e=1}^{\check {\mathrm{k}}} \frac{{\mathrm{\v{T} }}_{e}}{\sum _{e=1}^{\check {\mathrm{k}}}{\mathrm{\v{T} }}_{e}} { \left\vert {\mathfrak{Y}}_{{\Xi }_{e}}^{IN} \right\vert }^{\mathrm{\gimel }} \right) }^{ \frac{1}{\mathrm{\gimel }} } \right) \end{array} \right) \end{eqnarray*}

and

\begin{eqnarray*}BCFSSPRWA \left( {\Xi }_{1}^{{}^{{^{\prime}}}},{\Xi }_{2}^{{}^{{^{\prime}}}},\ldots ,{\Xi }_{\check {\mathrm{ k}}}^{{}^{{^{\prime}}}} \right) \nonumber\\\displaystyle = \left( \begin{array}{@{}c@{}} \displaystyle 1-{ \left( \sum _{e=1}^{\check {\mathrm{k}}} \frac{{\mathrm{\v{T} }}_{e}}{\sum _{e=1}^{\check {\mathrm{k}}}{\mathrm{\v{T} }}_{e}} { \left( 1-{\mathfrak{Y}}_{{\Xi }_{e}^{{}^{{^{\prime}}}}}^{RP} \right) }^{\mathrm{\gimel }} \right) }^{ \frac{1}{\mathrm{\gimel }} }+\iota \left( 1-{ \left( \sum _{e=1}^{\check {\mathrm{k}}} \frac{{\mathrm{\v{T} }}_{e}}{\sum _{e=1}^{\check {\mathrm{k}}}{\mathrm{\v{T} }}_{e}} { \left( 1-{\mathfrak{Y}}_{{\Xi }_{e}^{{}^{{^{\prime}}}}}^{IP} \right) }^{\mathrm{\gimel }} \right) }^{ \frac{1}{\mathrm{\gimel }} } \right) ,\\ \displaystyle -{ \left( \sum _{e=1}^{\check {\mathrm{k}}} \frac{{\mathrm{\v{T} }}_{e}}{\sum _{e=1}^{\check {\mathrm{k}}}{\mathrm{\v{T} }}_{e}} { \left\vert {\mathfrak{Y}}_{{\Xi }_{e}^{{}^{{^{\prime}}}}}^{RN} \right\vert }^{\mathrm{\gimel }} \right) }^{ \frac{1}{\mathrm{\gimel }} }+\iota \left( -{ \left( \sum _{e=1}^{\check {\mathrm{k}}} \frac{{\mathrm{\v{T} }}_{e}}{\sum _{e=1}^{\check {\mathrm{k}}}{\mathrm{\v{T} }}_{e}} { \left\vert {\mathfrak{Y}}_{{\Xi }_{e}^{{}^{{^{\prime}}}}}^{IN} \right\vert }^{\mathrm{\gimel }} \right) }^{ \frac{1}{\mathrm{\gimel }} } \right) \end{array} \right) \end{eqnarray*}

since ${\mathfrak{Y}}_{{\Xi }_{e}}^{RP}\leq {\mathfrak{Y}}_{{\Xi }_{e}^{{}^{{^{\prime}}}}}^{RP}\forall e$ thus

\begin{eqnarray*}{ \left( 1-{\mathfrak{Y}}_{{\Xi }_{e}}^{RP} \right) }^{\mathrm{\gimel }}\leq { \left( 1-{\mathfrak{Y}}_{{\Xi }_{e}^{{}^{{^{\prime}}}}}^{RP} \right) }^{\mathrm{\gimel }}\nonumber\\\displaystyle \Rightarrow { \left( \sum _{e=1}^{\check {\mathrm{k}}} \frac{{\mathrm{\v{T} }}_{e}}{\sum _{e=1}^{\check {\mathrm{k}}}{\mathrm{\v{T} }}_{e}} { \left( 1-{\mathfrak{Y}}_{{\Xi }_{e}}^{RP} \right) }^{\mathrm{\gimel }} \right) }^{ \frac{1}{\mathrm{\gimel }} }\leq { \left( \sum _{e=1}^{\check {\mathrm{k}}} \frac{{\mathrm{\v{T} }}_{e}}{\sum _{e=1}^{\check {\mathrm{k}}}{\mathrm{\v{T} }}_{e}} { \left( 1-{\mathfrak{Y}}_{{\Xi }_{e}^{{}^{{^{\prime}}}}}^{RP} \right) }^{\mathrm{\gimel }} \right) }^{ \frac{1}{\mathrm{\gimel }} }\nonumber\\\displaystyle \Rightarrow 1-{ \left( \sum _{e=1}^{\check {\mathrm{k}}} \frac{{\mathrm{\v{T} }}_{e}}{\sum _{e=1}^{\check {\mathrm{k}}}{\mathrm{\v{T} }}_{e}} { \left( 1-{\mathfrak{Y}}_{{\Xi }_{e}}^{RP} \right) }^{\mathrm{\gimel }} \right) }^{ \frac{1}{\mathrm{\gimel }} }\leq 1-{ \left( \sum _{e=1}^{\check {\mathrm{k}}} \frac{{\mathrm{\v{T} }}_{e}}{\sum _{e=1}^{\check {\mathrm{k}}}{\mathrm{\v{T} }}_{e}} { \left( 1-{\mathfrak{Y}}_{{\Xi }_{e}^{{}^{{^{\prime}}}}}^{RP} \right) }^{\mathrm{\gimel }} \right) }^{ \frac{1}{\mathrm{\gimel }} } \end{eqnarray*}

As ${\mathfrak{Y}}_{{\Xi }_{e}}^{IP}\leq {\mathfrak{Y}}_{{\Xi }_{e}^{{}^{{^{\prime}}}}}^{IP}\forall e$ similarly we have

\begin{eqnarray*}1-{ \left( \sum _{e=1}^{\check {\mathrm{k}}} \frac{{\mathrm{\v{T} }}_{e}}{\sum _{e=1}^{\check {\mathrm{k}}}{\mathrm{\v{T} }}_{e}} { \left( 1-{\mathfrak{Y}}_{{\Xi }_{e}}^{IP} \right) }^{\mathrm{\gimel }} \right) }^{ \frac{1}{\mathrm{\gimel }} }\leq 1-{ \left( \sum _{e=1}^{\check {\mathrm{k}}} \frac{{\mathrm{\v{T} }}_{e}}{\sum _{e=1}^{\check {\mathrm{k}}}{\mathrm{\v{T} }}_{e}} { \left( 1-{\mathfrak{Y}}_{{\Xi }_{e}^{{}^{{^{\prime}}}}}^{IP} \right) }^{\mathrm{\gimel }} \right) }^{ \frac{1}{\mathrm{\gimel }} } \end{eqnarray*}

therefore,

\begin{eqnarray*}1-{ \left( \sum _{e=1}^{\check {\mathrm{k}}} \frac{{\mathrm{\v{T} }}_{e}}{\sum _{e=1}^{\check {\mathrm{k}}}{\mathrm{\v{T} }}_{e}} { \left( 1-{\mathfrak{Y}}_{{\Xi }_{e}}^{RP} \right) }^{\mathrm{\gimel }} \right) }^{ \frac{1}{\mathrm{\gimel }} }+\iota \left( 1-{ \left( \sum _{e=1}^{\check {\mathrm{k}}} \frac{{\mathrm{\v{T} }}_{e}}{\sum _{e=1}^{\check {\mathrm{k}}}{\mathrm{\v{T} }}_{e}} { \left( 1-{\mathfrak{Y}}_{{\Xi }_{e}}^{IP} \right) }^{\mathrm{\gimel }} \right) }^{ \frac{1}{\mathrm{\gimel }} } \right) \nonumber\\\displaystyle \leq 1-{ \left( \sum _{e=1}^{\check {\mathrm{k}}} \frac{{\mathrm{\v{T} }}_{e}}{\sum _{e=1}^{\check {\mathrm{k}}}{\mathrm{\v{T} }}_{e}} { \left( 1-{\mathfrak{Y}}_{{\Xi }_{e}^{{}^{{^{\prime}}}}}^{RP} \right) }^{\mathrm{\gimel }} \right) }^{ \frac{1}{\mathrm{\gimel }} }+\iota \left( 1-{ \left( \sum _{e=1}^{\check {\mathrm{k}}} \frac{{\mathrm{\v{T} }}_{e}}{\sum _{e=1}^{\check {\mathrm{k}}}{\mathrm{\v{T} }}_{e}} { \left( 1-{\mathfrak{Y}}_{{\Xi }_{e}^{{}^{{^{\prime}}}}}^{IP} \right) }^{\mathrm{\gimel }} \right) }^{ \frac{1}{\mathrm{\gimel }} } \right) \end{eqnarray*}

Next since ${\mathfrak{Y}}_{{\Xi }_{e}}^{RN}\leq {\mathfrak{Y}}_{{\Xi }_{e}^{{}^{{^{\prime}}}}}^{RN}\forall e$ and ${\mathfrak{Y}}_{{\Xi }_{e}}^{RN},{\mathfrak{Y}}_{{\Xi }_{e}^{{}^{{^{\prime}}}}}^{RN}\in \left[ -1,0 \right] $, thus

\begin{eqnarray*} \left\vert {\mathfrak{Y}}_{{\Xi }_{e}}^{RN} \right\vert \geq \left\vert {\mathfrak{Y}}_{{\Xi }_{e}^{{}^{{^{\prime}}}}}^{RN} \right\vert \Rightarrow \sum _{e=1}^{\check {\mathrm{k}}} \frac{{\mathrm{\v{T} }}_{e}}{\sum _{e=1}^{\check {\mathrm{k}}}{\mathrm{\v{T} }}_{e}} { \left\vert {\mathfrak{Y}}_{{\Xi }_{e}}^{RN} \right\vert }^{\mathrm{\gimel }}\geq \sum _{e=1}^{\check {\mathrm{k}}} \frac{{\mathrm{\v{T} }}_{e}}{\sum _{e=1}^{\check {\mathrm{k}}}{\mathrm{\v{T} }}_{e}} { \left\vert {\mathfrak{Y}}_{{\Xi }_{e}^{{}^{{^{\prime}}}}}^{RN} \right\vert }^{\mathrm{\gimel }}\nonumber\\\displaystyle \Rightarrow { \left( \sum _{e=1}^{\check {\mathrm{k}}} \frac{{\mathrm{\v{T} }}_{e}}{\sum _{e=1}^{\check {\mathrm{k}}}{\mathrm{\v{T} }}_{e}} { \left\vert {\mathfrak{Y}}_{{\Xi }_{e}}^{RN} \right\vert }^{\mathrm{\gimel }} \right) }^{ \frac{1}{\mathrm{\gimel }} }\geq { \left( \sum _{e=1}^{\check {\mathrm{k}}} \frac{{\mathrm{\v{T} }}_{e}}{\sum _{e=1}^{\check {\mathrm{k}}}{\mathrm{\v{T} }}_{e}} { \left\vert {\mathfrak{Y}}_{{\Xi }_{e}^{{}^{{^{\prime}}}}}^{RN} \right\vert }^{\mathrm{\gimel }} \right) }^{ \frac{1}{\mathrm{\gimel }} }\nonumber\\\displaystyle \Rightarrow -{ \left( \sum _{e=1}^{\check {\mathrm{k}}} \frac{{\mathrm{\v{T} }}_{e}}{\sum _{e=1}^{\check {\mathrm{k}}}{\mathrm{\v{T} }}_{e}} { \left\vert {\mathfrak{Y}}_{{\Xi }_{e}}^{RN} \right\vert }^{\mathrm{\gimel }} \right) }^{ \frac{1}{\mathrm{\gimel }} }\leq -{ \left( \sum _{e=1}^{\check {\mathrm{k}}} \frac{{\mathrm{\v{T} }}_{e}}{\sum _{e=1}^{\check {\mathrm{k}}}{\mathrm{\v{T} }}_{e}} { \left\vert {\mathfrak{Y}}_{{\Xi }_{e}^{{}^{{^{\prime}}}}}^{RN} \right\vert }^{\mathrm{\gimel }} \right) }^{ \frac{1}{\mathrm{\gimel }} } \end{eqnarray*}

As ${\mathfrak{Y}}_{{\Xi }_{e}}^{IN}\leq {\mathfrak{Y}}_{{\Xi }_{e}^{{}^{{^{\prime}}}}}^{IN}\forall e$ similarly we have

\begin{eqnarray*}-{ \left( \sum _{e=1}^{\check {\mathrm{k}}} \frac{{\mathrm{\v{T} }}_{e}}{\sum _{e=1}^{\check {\mathrm{k}}}{\mathrm{\v{T} }}_{e}} { \left\vert {\mathfrak{Y}}_{{\Xi }_{e}}^{IN} \right\vert }^{\mathrm{\gimel }} \right) }^{ \frac{1}{\mathrm{\gimel }} }\geq -{ \left( \sum _{e=1}^{\check {\mathrm{k}}} \frac{{\mathrm{\v{T} }}_{e}}{\sum _{e=1}^{\check {\mathrm{k}}}{\mathrm{\v{T} }}_{e}} { \left\vert {\mathfrak{Y}}_{{\Xi }_{e}^{{}^{{^{\prime}}}}}^{IN} \right\vert }^{\mathrm{\gimel }} \right) }^{ \frac{1}{\mathrm{\gimel }} } \end{eqnarray*}

therefore,

\begin{eqnarray*}-{ \left( \sum _{e=1}^{\check {\mathrm{k}}}{\mathfrak{K}}_{e}{ \left\vert {\mathfrak{Y}}_{{\Xi }_{e}}^{PN} \right\vert }^{\mathrm{\gimel }} \right) }^{ \frac{1}{\mathrm{\gimel }} }+\iota \left( -{ \left( \sum _{e=1}^{\check {\mathrm{k}}}{\mathfrak{K}}_{e}{ \left\vert {\mathfrak{Y}}_{{\Xi }_{e}}^{IN} \right\vert }^{\mathrm{\gimel }} \right) }^{ \frac{1}{\mathrm{\gimel }} } \right) \geq -{ \left( \sum _{e=1}^{\check {\mathrm{k}}}{\mathfrak{K}}_{e}{ \left\vert {\mathfrak{Y}}_{{\Xi }_{e}^{{}^{{^{\prime}}}}}^{RN} \right\vert }^{\mathrm{\gimel }} \right) }^{ \frac{1}{\mathrm{\gimel }} }\nonumber\\\displaystyle \chskip[1pc]+\iota \left( -{ \left( \sum _{e=1}^{\check {\mathrm{k}}}{\mathfrak{K}}_{e}{ \left\vert {\mathfrak{Y}}_{{\Xi }_{e}^{{}^{{^{\prime}}}}}^{IN} \right\vert }^{\mathrm{\gimel }} \right) }^{ \frac{1}{\mathrm{\gimel }} } \right) \end{eqnarray*}

therefore,

\begin{eqnarray*}BCFSSPRWA \left( {\Xi }_{1},{\Xi }_{2},\ldots ,{\Xi }_{\check {\mathrm{k}}} \right) \leq BCFSSPRWA \left( {\Xi }_{1}^{{}^{{^{\prime}}}},{\Xi }_{2}^{{}^{{^{\prime}}}},\ldots ,{\Xi }_{\check {\mathrm{ k}}}^{{}^{{^{\prime}}}} \right) \end{eqnarray*}

**Axiom 3: (Boundedness)** Consider ${\Xi }_{e}= \left( {\mathfrak{Y}}_{{\Xi }_{e}}^{P},{\mathfrak{Y}}_{{\Xi }_{e}}^{N} \right) = \left( {\mathfrak{Y}}_{{\Xi }_{e}}^{RP}+\iota {\mathfrak{Y}}_{{\Xi }_{e}}^{IP},{\mathfrak{Y}}_{{\Xi }_{e}}^{RN}+\iota {\mathfrak{Y}}_{{\Xi }_{e}}^{IN} \right) ,e=1,2,...,\check {\mathrm{k}}$ as a group of BCFNs and if ${\Xi }^{-}= \left( {\scriptsize \begin{array}{@{}c@{}} \displaystyle {\min }_{e} \left\{ {\mathfrak{Y}}_{{\Xi }_{e}}^{RP} \right\} +\iota {\min }_{e} \left\{ {\mathfrak{Y}}_{{\Xi }_{BCFS-e}}^{IP} \right\} ,\\ \displaystyle {\max }_{e} \left\{ {\mathfrak{Y}}_{{\Xi }_{e}}^{RN} \right\} +\iota {\max }_{e} \left\{ {\mathfrak{Y}}_{{\Xi }_{e}}^{IN} \right\} \end{array}} \right) ,$ and ${\Xi }^{+}= \left( {\scriptsize \begin{array}{@{}c@{}} \displaystyle {\max }_{e} \left\{ {\mathfrak{Y}}_{{\Xi }_{e}}^{RP} \right\} +\iota {\max }_{e} \left\{ {\mathfrak{Y}}_{{\Xi }_{e}}^{IP} \right\} ,\\ \displaystyle {\min }_{e} \left\{ {\mathfrak{Y}}_{{\Xi }_{e}}^{RN} \right\} +\iota {\min }_{e} \left\{ {\mathfrak{Y}}_{{\Xi }_{e}}^{IN} \right\} \end{array}} \right) $, then

\begin{eqnarray*}{\Xi }^{-}\leq BCFSSPRWA \left( {\Xi }_{1},{\Xi }_{2},\ldots ,{\Xi }_{\check {\mathrm{k}}} \right) \leq {\Xi }^{+} \end{eqnarray*}

**Proof:** By employing Axiom 1 and 2, we have

\begin{eqnarray*}BCFSSPRWA \left( {\Xi }_{1},{\Xi }_{2},\ldots ,{\Xi }_{\check {\mathrm{k}}} \right) & \leq BCFSSPRWA \left( {\Xi }_{1}^{+},{\Xi }_{2}^{+},\ldots ,{\Xi }_{\check {\mathrm{ k}}}^{+} \right) ={\Xi }^{+} \end{eqnarray*}

\begin{eqnarray*}BCFSSPRWA \left( {\Xi }_{1},{\Xi }_{2},\ldots ,{\Xi }_{\check {\mathrm{k}}} \right) & \geq BCFSSPRWA \left( {\Xi }_{1}^{-},{\Xi }_{2}^{-},\ldots ,{\Xi }_{\check {\mathrm{ k}}}^{-} \right) ={\Xi }^{-} \end{eqnarray*}

We get

\begin{eqnarray*}{\Xi }^{+}\leq BCFSSPRWA \left( {\Xi }_{1},{\Xi }_{2},\ldots ,{\Xi }_{\check {\mathrm{k}}} \right) \leq {\Xi }^{+} \end{eqnarray*}

**Definition 9:** Consider ${\Xi }_{e}= \left( {\mathfrak{Y}}_{{\Xi }_{e}}^{P},{\mathfrak{Y}}_{{\Xi }_{e}}^{N} \right) = \left( {\mathfrak{Y}}_{{\Xi }_{e}}^{RP}+\iota {\mathfrak{Y}}_{{\Xi }_{e}}^{IP},{\mathfrak{Y}}_{{\Xi }_{e}}^{RN}+\iota {\mathfrak{Y}}_{{\Xi }_{e}}^{IN} \right) ,e=1,2,..,\check {\mathrm{k}}$ as a group of BCFNs, then

(10) \begin{eqnarray*}BCFSSPROWA \left( {\Xi }_{1},{\Xi }_{2},\ldots ,{\Xi }_{\check {\mathrm{k}}} \right) =\begin{array}{@{}c@{}} \displaystyle \check {\mathrm{k}}\\ \displaystyle \oplus \\ \displaystyle e=1 \end{array} \frac{{\mathrm{\v{T} }}_{e}}{\sum _{e=1}^{\check {\mathrm{k}}}{\mathrm{\v{T} }}_{e}} {\Xi }_{\varsigma \left( e \right) }\end{eqnarray*}

is introduced as BCFSSPROWA operator, where Ť_1_ = 1, ${\mathrm{\v{T} }}_{e}={\mathop{\prod }\nolimits }_{e=1}^{\check {\mathrm{k}}-1}{\mathfrak{S}}_{\mathfrak{B}} \left( {\Xi }_{e} \right) ,e=1,2,\ldots ,\check {\mathrm{k}},{\mathfrak{S}}_{\mathfrak{B}} \left( {\Xi }_{e} \right) $ would deduce the score value of BCFN Ξ_*ȅ*_ and $ \left( \varsigma \left( 1 \right) ,\varsigma \left( 2 \right) ,..,\varsigma \left( \mathrm{\v{T} } \right) \right) $ is a permutation of $ \left( 1,2,..,\check {\mathrm{k}} \right) $ with ∀̦*e* ${\Xi }_{\varsigma \left( e-1 \right) }\geq {\Xi }_{\varsigma \left( e \right) }$.

**Theorem 2:** Consider ${\Xi }_{e}= \left( {\mathfrak{Y}}_{{\Xi }_{e}}^{P},{\mathfrak{Y}}_{{\Xi }_{e}}^{N} \right) = \left( {\mathfrak{Y}}_{{\Xi }_{e}}^{RP}+\iota {\mathfrak{Y}}_{{\Xi }_{e}}^{IP},{\mathfrak{Y}}_{{\Xi }_{e}}^{RN}+\iota {\mathfrak{Y}}_{{\Xi }_{e}}^{IN} \right) ,e=1,2,...,\check {\mathrm{k}}$ as a group of BCFNs, then by utilizing BCFSSPROWA, we achieve the aggregated result and

(11) \begin{eqnarray*}BCFSSPROWA \left( {\Xi }_{1},{\Xi }_{2},\ldots ,{\Xi }_{\check {\mathrm{k}}} \right) \nonumber\\\displaystyle = \left( \begin{array}{@{}c@{}} \displaystyle 1-{ \left( \sum _{e=1}^{\check {\mathrm{k}}} \frac{{\mathrm{\v{T} }}_{e}}{\sum _{e=1}^{\check {\mathrm{k}}}{\mathrm{\v{T} }}_{e}} { \left( 1-{\mathfrak{Y}}_{{\Xi }_{\varsigma \left( e \right) }}^{RP} \right) }^{\mathrm{\gimel }} \right) }^{ \frac{1}{\mathrm{\gimel }} }+\iota \left( 1-{ \left( \sum _{e=1}^{\check {\mathrm{k}}} \frac{{\mathrm{\v{T} }}_{e}}{\sum _{e=1}^{\check {\mathrm{k}}}{\mathrm{\v{T} }}_{e}} { \left( 1-{\mathfrak{Y}}_{{\Xi }_{\varsigma \left( e \right) }}^{IP} \right) }^{\mathrm{\gimel }} \right) }^{ \frac{1}{\mathrm{\gimel }} } \right) ,\\ \displaystyle -{ \left( \sum _{e=1}^{\check {\mathrm{k}}} \frac{{\mathrm{\v{T} }}_{e}}{\sum _{e=1}^{\check {\mathrm{k}}}{\mathrm{\v{T} }}_{e}} { \left\vert {\mathfrak{Y}}_{{\Xi }_{\varsigma \left( e \right) }}^{RN} \right\vert }^{\mathrm{\gimel }} \right) }^{ \frac{1}{\mathrm{\gimel }} }+\iota \left( -{ \left( \sum _{e=1}^{\check {\mathrm{k}}} \frac{{\mathrm{\v{T} }}_{e}}{\sum _{e=1}^{\check {\mathrm{k}}}{\mathrm{\v{T} }}_{e}} { \left\vert {\mathfrak{Y}}_{{\Xi }_{\varsigma \left( e \right) }}^{IN} \right\vert }^{\mathrm{\gimel }} \right) }^{ \frac{1}{\mathrm{\gimel }} } \right) \end{array} \right) \end{eqnarray*}

**Axiom 4: (Idempotency)** Consider ${\Xi }_{e}= \left( {\mathfrak{Y}}_{{\Xi }_{e}}^{P},{\mathfrak{Y}}_{{\Xi }_{e}}^{N} \right) = \left( {\mathfrak{Y}}_{{\Xi }_{e}}^{RP}+\iota {\mathfrak{Y}}_{{\Xi }_{e}}^{IP},{\mathfrak{Y}}_{{\Xi }_{e}}^{RN}+\iota {\mathfrak{Y}}_{{\Xi }_{e}}^{IN} \right) ,e=1,2,..,\check {\mathrm{k}}$ as a group of BCFNs, and if ${\Xi }_{e}=\Xi = \left( {\mathfrak{Y}}_{\Xi }^{P},{\mathfrak{Y}}_{\Xi }^{N} \right) = \left( {\mathfrak{Y}}_{\Xi }^{RP}+\iota {\mathfrak{Y}}_{\Xi }^{IP},{\mathfrak{Y}}_{\Xi }^{RN}+\iota {\mathfrak{Y}}_{\Xi }^{IN} \right) \forall e$, then

\begin{eqnarray*}BCFSSPROWA \left( {\Xi }_{1},{\Xi }_{2},\ldots ,{\Xi }_{\check {\mathrm{k}}} \right) =\Xi \end{eqnarray*}

**Axiom 5: (Monotonicity)** Consider ${\Xi }_{e}= \left( {\mathfrak{Y}}_{{\Xi }_{e}}^{P},{\mathfrak{Y}}_{{\Xi }_{e}}^{N} \right) = \left( {\mathfrak{Y}}_{{\Xi }_{e}}^{RP}+\iota {\mathfrak{Y}}_{{\Xi }_{e}}^{IP},{\mathfrak{Y}}_{{\Xi }_{e}}^{RN}+\iota {\mathfrak{Y}}_{{\Xi }_{e}}^{IN} \right) ,$ and ${\Xi }_{e}^{{}^{{^{\prime}}}}= \left( {\mathfrak{Y}}_{{\Xi }_{e}^{{}^{{^{\prime}}}}}^{P},{\mathfrak{Y}}_{{\Xi }_{e}^{{}^{{^{\prime}}}}}^{N} \right) = \left( {\mathfrak{Y}}_{{\Xi }_{e}^{{}^{{^{\prime}}}}}^{RP}+\iota {\mathfrak{Y}}_{{\Xi }_{e}^{{}^{{^{\prime}}}}}^{IP},{\mathfrak{Y}}_{{\Xi }_{e}^{{}^{{^{\prime}}}}}^{RN}+\iota {\mathfrak{Y}}_{{\Xi }_{e}^{{}^{{^{\prime}}}}}^{IN} \right) ,e=1,2,..,\check {\mathrm{k}}$ as two groups of BCFNs, and if ${\mathfrak{Y}}_{{\Xi }_{e}}^{RP}\leq {\mathfrak{Y}}_{{\Xi }_{e}^{{}^{{^{\prime}}}}}^{RP}$, ${\mathfrak{Y}}_{{\Xi }_{e}}^{IP}\leq {\mathfrak{Y}}_{{\Xi }_{e}^{{}^{{^{\prime}}}}}^{IP}$, ${\mathfrak{Y}}_{{\Xi }_{e}}^{RN}\leq {\mathfrak{Y}}_{{\Xi }_{e}^{{}^{{^{\prime}}}}}^{RN}$, and ${\mathfrak{Y}}_{{\Xi }_{e}}^{IN}\leq {\mathfrak{Y}}_{{\Xi }_{e}^{{}^{{^{\prime}}}}}^{IN}$ , then

\begin{eqnarray*}BCFSSPROWA \left( {\Xi }_{1},{\Xi }_{2},\ldots ,{\Xi }_{\check {\mathrm{k}}} \right) \leq BCFSSPROWA \left( {\Xi }_{1}^{{}^{{^{\prime}}}},{\Xi }_{2}^{{}^{{^{\prime}}}},\ldots ,{\Xi }_{\check {\mathrm{ k}}}^{{}^{{^{\prime}}}} \right) \end{eqnarray*}

**Axiom 6: (Boundedness)** Consider ${\Xi }_{e}= \left( {\mathfrak{Y}}_{{\Xi }_{e}}^{P},{\mathfrak{Y}}_{{\Xi }_{e}}^{N} \right) = \left( {\mathfrak{Y}}_{{\Xi }_{e}}^{RP}+\iota {\mathfrak{Y}}_{{\Xi }_{e}}^{IP},{\mathfrak{Y}}_{{\Xi }_{e}}^{RN}+\iota {\mathfrak{Y}}_{{\Xi }_{e}}^{IN} \right) ,e=1,2,..,\check {\mathrm{k}}$ as a group of BCFNs and if ${\Xi }^{-}= \left( {\scriptsize \begin{array}{@{}c@{}} \displaystyle {\min }_{e} \left\{ {\mathfrak{Y}}_{{\Xi }_{e}}^{RP} \right\} +\iota {\min }_{e} \left\{ {\mathfrak{Y}}_{{\Xi }_{e}}^{IP} \right\} ,\\ \displaystyle {\max }_{e} \left\{ {\mathfrak{Y}}_{{\Xi }_{e}}^{RN} \right\} +\iota {\max }_{e} \left\{ {\mathfrak{Y}}_{{\Xi }_{e}}^{IN} \right\} \end{array}} \right) ,$ and ${\Xi }^{+}= \left( {\scriptsize \begin{array}{@{}c@{}} \displaystyle {\max }_{e} \left\{ {\mathfrak{Y}}_{{\Xi }_{e}}^{RP} \right\} +\iota {\max }_{e} \left\{ {\mathfrak{Y}}_{{\Xi }_{e}}^{IP} \right\} ,\\ \displaystyle {\min }_{e} \left\{ {\mathfrak{Y}}_{{\Xi }_{e}}^{RN} \right\} +\iota {\min }_{e} \left\{ {\mathfrak{Y}}_{{\Xi }_{e}}^{IN} \right\} \end{array}} \right) $, then

\begin{eqnarray*}{\Xi }^{-}\leq BCFSSPROWA \left( {\Xi }_{1},{\Xi }_{2},\ldots ,{\Xi }_{\check {\mathrm{k}}} \right) \leq {\Xi }^{+} \end{eqnarray*}

**Definition 10:** Consider ${\Xi }_{e}= \left( {\mathfrak{Y}}_{{\Xi }_{e}}^{P},{\mathfrak{Y}}_{{\Xi }_{e}}^{N} \right) = \left( {\mathfrak{Y}}_{{\Xi }_{e}}^{RP}+\iota {\mathfrak{Y}}_{{\Xi }_{e}}^{IP},{\mathfrak{Y}}_{{\Xi }_{e}}^{RN}+\iota {\mathfrak{Y}}_{{\Xi }_{e}}^{IN} \right) ,e=1,2,..,\check {\mathrm{k}}$ as a group of BCFNs, then

(12) \begin{eqnarray*}BCFSSPRWG \left( {\Xi }_{1},{\Xi }_{2},\ldots ,{\Xi }_{\check {\mathrm{k}}} \right) =\begin{array}{@{}c@{}} \displaystyle \check {\mathrm{k}}\\ \displaystyle \otimes \\ \displaystyle e=1 \end{array}{ \left( {\Xi }_{e} \right) }^{ \frac{{\mathrm{\v{T} }}_{e}}{\sum _{e=1}^{\check {\mathrm{k}}}{\mathrm{\v{T} }}_{e}} }\end{eqnarray*}

is introduced as BCFSSPRWG operator, where Ť_1_ = 1, ${\mathrm{\v{T} }}_{e}={\mathop{\prod }\nolimits }_{e=1}^{\mathrm{\v{T} }-1}{\mathfrak{S}}_{\mathfrak{B}} \left( {\Xi }_{e} \right) ,e=1,2,\ldots ,\check {\mathrm{k}}$ and ${\mathfrak{S}}_{\mathfrak{B}} \left( {\Xi }_{e} \right) $ would deduce the score value of BCFN Ξ_*ȅ*_.

**Theorem 3:** Consider ${\Xi }_{e}= \left( {\mathfrak{Y}}_{{\Xi }_{e}}^{P},{\mathfrak{Y}}_{{\Xi }_{e}}^{N} \right) = \left( {\mathfrak{Y}}_{{\Xi }_{e}}^{RP}+\iota {\mathfrak{Y}}_{{\Xi }_{e}}^{IP},{\mathfrak{Y}}_{{\Xi }_{e}}^{RN}+\iota {\mathfrak{Y}}_{{\Xi }_{e}}^{IN} \right) ,e=1,2,..,\check {\mathrm{k}}$ as a group of BCFNs, then by utilizing BCFSSPRWG, we achieve the aggregated result and

(13) \begin{eqnarray*}BCFSSPRWG \left( {\Xi }_{1},{\Xi }_{2},\ldots ,{\Xi }_{\check {\mathrm{k}}} \right) =\nonumber\\\displaystyle \left( \begin{array}{@{}c@{}} \displaystyle { \left( \sum _{e=1}^{\check {\mathrm{k}}} \frac{{\mathrm{\v{T} }}_{e}}{\sum _{e=1}^{\check {\mathrm{k}}}{\mathrm{\v{T} }}_{e}} { \left( {\mathfrak{Y}}_{{\Xi }_{e}}^{RP} \right) }^{\mathrm{\gimel }} \right) }^{ \frac{1}{\mathrm{\gimel }} }+\iota { \left( \sum _{e=1}^{\check {\mathrm{k}}} \frac{{\mathrm{\v{T} }}_{e}}{\sum _{e=1}^{\check {\mathrm{k}}}{\mathrm{\v{T} }}_{e}} { \left( {\mathfrak{Y}}_{{\Xi }_{e}}^{IP} \right) }^{\mathrm{\gimel }} \right) }^{ \frac{1}{\mathrm{\gimel }} },\\ \displaystyle -1+{ \left( \sum _{e=1}^{\check {\mathrm{k}}} \frac{{\mathrm{\v{T} }}_{e}}{\sum _{e=1}^{\check {\mathrm{k}}}{\mathrm{\v{T} }}_{e}} { \left( 1+{\mathfrak{Y}}_{{\Xi }_{e}}^{RN} \right) }^{\mathrm{\gimel }} \right) }^{ \frac{1}{\mathrm{\gimel }} }+\iota \left( -1+{ \left( \sum _{e=1}^{\check {\mathrm{k}}} \frac{{\mathrm{\v{T} }}_{e}}{\sum _{e=1}^{\check {\mathrm{k}}}{\mathrm{\v{T} }}_{e}} { \left( 1+{\mathfrak{Y}}_{{\Xi }_{e}}^{IN} \right) }^{\mathrm{\gimel }} \right) }^{ \frac{1}{\mathrm{\gimel }} } \right) \end{array} \right) \end{eqnarray*}

**Proof:** Equation (13) can be rewritten as underneath

(14) \begin{eqnarray*}BCFSSPRWG \left( {\Xi }_{1},{\Xi }_{2},\ldots ,{\Xi }_{\check {\mathrm{k}}} \right) =\nonumber\\\displaystyle \left( \begin{array}{@{}c@{}} \displaystyle { \left( \sum _{e=1}^{\check {\mathrm{k}}} \frac{{\mathrm{\v{T} }}_{e}}{\sum _{e=1}^{\check {\mathrm{k}}}{\mathrm{\v{T} }}_{e}} { \left( {\mathfrak{Y}}_{{\Xi }_{e}}^{RP} \right) }^{\mathrm{\gimel }}-\sum _{e=1}^{\check {\mathrm{k}}} \frac{{\mathrm{\v{T} }}_{e}}{\sum _{e=1}^{\check {\mathrm{k}}}{\mathrm{\v{T} }}_{e}} +1 \right) }^{ \frac{1}{\mathrm{\gimel }} }\\ \displaystyle +\iota { \left( \sum _{e=1}^{\check {\mathrm{k}}} \frac{{\mathrm{\v{T} }}_{e}}{\sum _{e=1}^{\check {\mathrm{k}}}{\mathrm{\v{T} }}_{e}} { \left( {\mathfrak{Y}}_{{\Xi }_{e}}^{IP} \right) }^{\mathrm{\gimel }}-\sum _{e=1}^{\check {\mathrm{k}}}{\mathfrak{K}}_{e}+1 \right) }^{ \frac{1}{\mathrm{\gimel }} },\\ \displaystyle -1+{ \left( \sum _{e=1}^{\check {\mathrm{k}}} \frac{{\mathrm{\v{T} }}_{e}}{\sum _{e=1}^{\check {\mathrm{k}}}{\mathrm{\v{T} }}_{e}} { \left( 1+{\mathfrak{Y}}_{{\Xi }_{e}}^{RN} \right) }^{\mathrm{\gimel }}-\sum _{e=1}^{\check {\mathrm{k}}}{\mathfrak{K}}_{e}+1 \right) }^{ \frac{1}{\mathrm{\gimel }} }\\ \displaystyle +\iota \left( -1+{ \left( \sum _{e=1}^{\check {\mathrm{k}}} \frac{{\mathrm{\v{T} }}_{e}}{\sum _{e=1}^{\check {\mathrm{k}}}{\mathrm{\v{T} }}_{e}} { \left( 1+{\mathfrak{Y}}_{{\Xi }_{e}}^{IN} \right) }^{\mathrm{\gimel }}-\sum _{e=1}^{\check {\mathrm{k}}}{\mathfrak{K}}_{e}+1 \right) }^{ \frac{1}{\mathrm{\gimel }} } \right) \end{array} \right) \end{eqnarray*}

Through mathematical induction, we would exhibit Eq. (14) is holds $\forall \check {\mathrm{k}}$. Let $\check {\mathrm{k}}=2$. Then

\begin{eqnarray*}{ \left( {\Xi }_{1} \right) }^{ \frac{{\mathrm{\v{T} }}_{e}}{\sum _{e=1}^{2}{\mathrm{\v{T} }}_{e}} }= \left( \begin{array}{@{}c@{}} \displaystyle { \left( \frac{{\mathrm{\v{T} }}_{1}}{\sum _{e=1}^{2}{\mathrm{\v{T} }}_{e}} { \left( {\mathfrak{Y}}_{{\Xi }_{1}}^{RP} \right) }^{\mathrm{\gimel }}- \left( \frac{{\mathrm{\v{T} }}_{1}}{\sum _{e=1}^{2}{\mathrm{\v{T} }}_{e}} -1 \right) \right) }^{ \frac{1}{\mathrm{\gimel }} }\\ \displaystyle +\iota { \left( \frac{{\mathrm{\v{T} }}_{1}}{\sum _{e=1}^{2}{\mathrm{\v{T} }}_{e}} { \left( {\mathfrak{Y}}_{{\Xi }_{1}}^{IP} \right) }^{\mathrm{\gimel }}- \left( \frac{{\mathrm{\v{T} }}_{1}}{\sum _{e=1}^{2}{\mathrm{\v{T} }}_{e}} -1 \right) \right) }^{ \frac{1}{\mathrm{\gimel }} },\\ \displaystyle -1+{ \left( \frac{{\mathrm{\v{T} }}_{1}}{\sum _{e=1}^{2}{\mathrm{\v{T} }}_{e}} { \left( 1+{\mathfrak{Y}}_{{\Xi }_{1}}^{RN} \right) }^{\mathrm{\gimel }}- \left( \frac{{\mathrm{\v{T} }}_{1}}{\sum _{e=1}^{2}{\mathrm{\v{T} }}_{e}} -1 \right) \right) }^{ \frac{1}{\mathrm{\gimel }} }\\ \displaystyle +\iota \left( -1+{ \left( \frac{{\mathrm{\v{T} }}_{1}}{\sum _{e=1}^{2}{\mathrm{\v{T} }}_{e}} { \left( 1+{\mathfrak{Y}}_{{\Xi }_{1}}^{IN} \right) }^{\mathrm{\gimel }}- \left( \frac{{\mathrm{\v{T} }}_{1}}{\sum _{e=1}^{2}{\mathrm{\v{T} }}_{e}} -1 \right) \right) }^{ \frac{1}{\mathrm{\gimel }} } \right) \end{array} \right) \end{eqnarray*}

and

\begin{eqnarray*}{ \left( {\Xi }_{2} \right) }^{ \frac{{\mathrm{\v{T} }}_{2}}{\sum _{e=1}^{2}{\mathrm{\v{T} }}_{e}} }= \left( \begin{array}{@{}c@{}} \displaystyle { \left( \frac{{\mathrm{\v{T} }}_{2}}{\sum _{e=1}^{2}{\mathrm{\v{T} }}_{e}} { \left( {\mathfrak{Y}}_{{\Xi }_{2}}^{RP} \right) }^{\mathrm{\gimel }}- \left( \frac{{\mathrm{\v{T} }}_{2}}{\sum _{e=1}^{2}{\mathrm{\v{T} }}_{e}} -1 \right) \right) }^{ \frac{1}{\mathrm{\gimel }} }\\ \displaystyle +\iota { \left( \frac{{\mathrm{\v{T} }}_{2}}{\sum _{e=1}^{2}{\mathrm{\v{T} }}_{e}} { \left( {\mathfrak{Y}}_{{\Xi }_{2}}^{IP} \right) }^{\mathrm{\gimel }}- \left( \frac{{\mathrm{\v{T} }}_{2}}{\sum _{e=1}^{2}{\mathrm{\v{T} }}_{e}} -1 \right) \right) }^{ \frac{1}{\mathrm{\gimel }} },\\ \displaystyle -1+{ \left( \frac{{\mathrm{\v{T} }}_{2}}{\sum _{e=1}^{2}{\mathrm{\v{T} }}_{e}} { \left( 1+{\mathfrak{Y}}_{{\Xi }_{2}}^{RN} \right) }^{\mathrm{\gimel }}- \left( \frac{{\mathrm{\v{T} }}_{2}}{\sum _{e=1}^{2}{\mathrm{\v{T} }}_{e}} -1 \right) \right) }^{ \frac{1}{\mathrm{\gimel }} }\\ \displaystyle +\iota \left( -1+{ \left( \frac{{\mathrm{\v{T} }}_{2}}{\sum _{e=1}^{2}{\mathrm{\v{T} }}_{e}} { \left( 1+{\mathfrak{Y}}_{{\Xi }_{2}}^{IN} \right) }^{\mathrm{\gimel }}- \left( \frac{{\mathrm{\v{T} }}_{2}}{\sum _{e=1}^{2}{\mathrm{\v{T} }}_{e}} -1 \right) \right) }^{ \frac{1}{\mathrm{\gimel }} } \right) \end{array} \right) \end{eqnarray*}

then,

\begin{eqnarray*}{ \left( {\Xi }_{1} \right) }^{ \frac{{\mathrm{\v{T} }}_{e}}{\sum _{e=1}^{2}{\mathrm{\v{T} }}_{e}} }{\otimes }_{\mathbb{ SS}}{ \left( {\Xi }_{2} \right) }^{ \frac{{\mathrm{\v{T} }}_{2}}{\sum _{e=1}^{2}{\mathrm{\v{T} }}_{e}} }= \left( \begin{array}{@{}c@{}} \displaystyle { \left( \frac{{\mathrm{\v{T} }}_{1}}{\sum _{e=1}^{2}{\mathrm{\v{T} }}_{e}} { \left( {\mathfrak{Y}}_{{\Xi }_{1}}^{RP} \right) }^{\mathrm{\gimel }}- \left( \frac{{\mathrm{\v{T} }}_{1}}{\sum _{e=1}^{2}{\mathrm{\v{T} }}_{e}} -1 \right) \right) }^{ \frac{1}{\mathrm{\gimel }} }\\ \displaystyle +\iota { \left( \frac{{\mathrm{\v{T} }}_{1}}{\sum _{e=1}^{2}{\mathrm{\v{T} }}_{e}} { \left( {\mathfrak{Y}}_{{\Xi }_{1}}^{IP} \right) }^{\mathrm{\gimel }}- \left( \frac{{\mathrm{\v{T} }}_{1}}{\sum _{e=1}^{2}{\mathrm{\v{T} }}_{e}} -1 \right) \right) }^{ \frac{1}{\mathrm{\gimel }} },\\ \displaystyle -1+{ \left( \frac{{\mathrm{\v{T} }}_{1}}{\sum _{e=1}^{2}{\mathrm{\v{T} }}_{e}} { \left( 1+{\mathfrak{Y}}_{{\Xi }_{1}}^{RN} \right) }^{\mathrm{\gimel }}- \left( \frac{{\mathrm{\v{T} }}_{1}}{\sum _{e=1}^{2}{\mathrm{\v{T} }}_{e}} -1 \right) \right) }^{ \frac{1}{\mathrm{\gimel }} }\\ \displaystyle +\iota \left( -1+{ \left( \frac{{\mathrm{\v{T} }}_{1}}{\sum _{e=1}^{2}{\mathrm{\v{T} }}_{e}} { \left( 1+{\mathfrak{Y}}_{{\Xi }_{1}}^{IN} \right) }^{\mathrm{\gimel }}- \left( \frac{{\mathrm{\v{T} }}_{1}}{\sum _{e=1}^{2}{\mathrm{\v{T} }}_{e}} -1 \right) \right) }^{ \frac{1}{\mathrm{\gimel }} } \right) \end{array} \right) \nonumber\\\displaystyle {\otimes }_{\mathbb{SS}} \left( \begin{array}{@{}c@{}} \displaystyle { \left( \frac{{\mathrm{\v{T} }}_{2}}{\sum _{e=1}^{2}{\mathrm{\v{T} }}_{e}} { \left( {\mathfrak{Y}}_{{\Xi }_{2}}^{RP} \right) }^{\mathrm{\gimel }}- \left( \frac{{\mathrm{\v{T} }}_{2}}{\sum _{e=1}^{2}{\mathrm{\v{T} }}_{e}} -1 \right) \right) }^{ \frac{1}{\mathrm{\gimel }} }\\ \displaystyle +\iota { \left( \frac{{\mathrm{\v{T} }}_{2}}{\sum _{e=1}^{2}{\mathrm{\v{T} }}_{e}} { \left( {\mathfrak{Y}}_{{\Xi }_{2}}^{IP} \right) }^{\mathrm{\gimel }}- \left( \frac{{\mathrm{\v{T} }}_{2}}{\sum _{e=1}^{2}{\mathrm{\v{T} }}_{e}} -1 \right) \right) }^{ \frac{1}{\mathrm{\gimel }} },\\ \displaystyle -1+{ \left( \frac{{\mathrm{\v{T} }}_{2}}{\sum _{e=1}^{2}{\mathrm{\v{T} }}_{e}} { \left( 1+{\mathfrak{Y}}_{{\Xi }_{2}}^{RN} \right) }^{\mathrm{\gimel }}- \left( \frac{{\mathrm{\v{T} }}_{2}}{\sum _{e=1}^{2}{\mathrm{\v{T} }}_{e}} -1 \right) \right) }^{ \frac{1}{\mathrm{\gimel }} }\\ \displaystyle +\iota \left( -1+{ \left( \frac{{\mathrm{\v{T} }}_{2}}{\sum _{e=1}^{2}{\mathrm{\v{T} }}_{e}} { \left( 1+{\mathfrak{Y}}_{{\Xi }_{2}}^{IN} \right) }^{\mathrm{\gimel }}- \left( \frac{{\mathrm{\v{T} }}_{2}}{\sum _{e=1}^{2}{\mathrm{\v{T} }}_{e}} -1 \right) \right) }^{ \frac{1}{\mathrm{\gimel }} } \right) \end{array} \right) \nonumber\\\displaystyle = \left( \begin{array}{@{}c@{}} \displaystyle \begin{array}{@{}c@{}} \displaystyle \begin{array}{@{}c@{}} \displaystyle { \left( \begin{array}{@{}c@{}} \displaystyle { \left( { \left( \frac{{\mathrm{\v{T} }}_{1}}{\sum _{e=1}^{2}{\mathrm{\v{T} }}_{e}} { \left( {\mathfrak{Y}}_{{\Xi }_{1}}^{RP} \right) }^{\mathrm{\gimel }}- \left( \frac{{\mathrm{\v{T} }}_{1}}{\sum _{e=1}^{2}{\mathrm{\v{T} }}_{e}} -1 \right) \right) }^{ \frac{1}{\mathrm{\gimel }} } \right) }^{\mathrm{\gimel }}\\ \displaystyle +{ \left( { \left( \frac{{\mathrm{\v{T} }}_{2}}{\sum _{e=1}^{2}{\mathrm{\v{T} }}_{e}} { \left( {\mathfrak{Y}}_{{\Xi }_{2}}^{RP} \right) }^{\mathrm{\gimel }}- \left( \frac{{\mathrm{\v{T} }}_{2}}{\sum _{e=1}^{2}{\mathrm{\v{T} }}_{e}} -1 \right) \right) }^{ \frac{1}{\mathrm{\gimel }} } \right) }^{\mathrm{\gimel }}-1 \end{array} \right) }^{ \frac{1}{\mathrm{\gimel }} }\\ \displaystyle +\iota \left( { \left( \begin{array}{@{}c@{}} \displaystyle { \left( { \left( \frac{{\mathrm{\v{T} }}_{1}}{\sum _{e=1}^{2}{\mathrm{\v{T} }}_{e}} { \left( {\mathfrak{Y}}_{{\Xi }_{1}}^{IP} \right) }^{\mathrm{\gimel }}- \left( \frac{{\mathrm{\v{T} }}_{1}}{\sum _{e=1}^{2}{\mathrm{\v{T} }}_{e}} -1 \right) \right) }^{ \frac{1}{\mathrm{\gimel }} } \right) }^{\mathrm{\gimel }}\\ \displaystyle +{ \left( { \left( \frac{{\mathrm{\v{T} }}_{2}}{\sum _{e=1}^{2}{\mathrm{\v{T} }}_{e}} { \left( {\mathfrak{Y}}_{{\Xi }_{2}}^{IP} \right) }^{\mathrm{\gimel }}- \left( \frac{{\mathrm{\v{T} }}_{2}}{\sum _{e=1}^{2}{\mathrm{\v{T} }}_{e}} -1 \right) \right) }^{ \frac{1}{\mathrm{\gimel }} } \right) }^{\mathrm{\gimel }}-1 \end{array} \right) }^{ \frac{1}{\mathrm{\gimel }} } \right) , \end{array}\\ \displaystyle -1+{ \left( \begin{array}{@{}c@{}} \displaystyle { \left( 1+ \left( -1+{ \left( \frac{{\mathrm{\v{T} }}_{1}}{\sum _{e=1}^{2}{\mathrm{\v{T} }}_{e}} { \left( 1+{\mathfrak{Y}}_{{\Xi }_{1}}^{RN} \right) }^{\mathrm{\gimel }}- \left( \frac{{\mathrm{\v{T} }}_{1}}{\sum _{e=1}^{2}{\mathrm{\v{T} }}_{e}} -1 \right) \right) }^{ \frac{1}{\mathrm{\gimel }} } \right) \right) }^{\mathrm{\gimel }}\\ \displaystyle +{ \left( 1+ \left( -1+{ \left( \frac{{\mathrm{\v{T} }}_{2}}{\sum _{e=1}^{2}{\mathrm{\v{T} }}_{e}} { \left( 1+{\mathfrak{Y}}_{{\Xi }_{2}}^{RN} \right) }^{\mathrm{\gimel }}- \left( \frac{{\mathrm{\v{T} }}_{2}}{\sum _{e=1}^{2}{\mathrm{\v{T} }}_{e}} -1 \right) \right) }^{ \frac{1}{\mathrm{\gimel }} } \right) \right) }^{\mathrm{\gimel }}-1 \end{array} \right) }^{ \frac{1}{\mathrm{\gimel }} } \end{array}\\ \displaystyle +\iota \left( -1+{ \left( \begin{array}{@{}c@{}} \displaystyle { \left( 1+ \left( -1+{ \left( \frac{{\mathrm{\v{T} }}_{1}}{\sum _{e=1}^{2}{\mathrm{\v{T} }}_{e}} { \left( 1+{\mathfrak{Y}}_{{\Xi }_{1}}^{IN} \right) }^{\mathrm{\gimel }}- \left( \frac{{\mathrm{\v{T} }}_{1}}{\sum _{e=1}^{2}{\mathrm{\v{T} }}_{e}} -1 \right) \right) }^{ \frac{1}{\mathrm{\gimel }} } \right) \right) }^{\mathrm{\gimel }}\\ \displaystyle +{ \left( 1+ \left( -1+{ \left( \frac{{\mathrm{\v{T} }}_{2}}{\sum _{e=1}^{2}{\mathrm{\v{T} }}_{e}} { \left( 1+{\mathfrak{Y}}_{{\Xi }_{2}}^{IN} \right) }^{\mathrm{\gimel }}- \left( \frac{{\mathrm{\v{T} }}_{2}}{\sum _{e=1}^{2}{\mathrm{\v{T} }}_{e}} -1 \right) \right) }^{ \frac{1}{\mathrm{\gimel }} } \right) \right) }^{\mathrm{\gimel }}-1 \end{array} \right) }^{ \frac{1}{\mathrm{\gimel }} } \right) \end{array} \right) \nonumber\\\displaystyle = \left( \begin{array}{@{}c@{}} \displaystyle \begin{array}{@{}c@{}} \displaystyle { \left( \begin{array}{@{}c@{}} \displaystyle \frac{{\mathrm{\v{T} }}_{1}}{\sum _{e=1}^{2}{\mathrm{\v{T} }}_{e}} { \left( {\mathfrak{Y}}_{{\Xi }_{1}}^{RP} \right) }^{\mathrm{\gimel }}- \left( \frac{{\mathrm{\v{T} }}_{1}}{\sum _{e=1}^{2}{\mathrm{\v{T} }}_{e}} -1 \right) \\ \displaystyle + \frac{{\mathrm{\v{T} }}_{2}}{\sum _{e=1}^{2}{\mathrm{\v{T} }}_{e}} { \left( {\mathfrak{Y}}_{{\Xi }_{2}}^{RP} \right) }^{\mathrm{\gimel }}- \left( \frac{{\mathrm{\v{T} }}_{2}}{\sum _{e=1}^{2}{\mathrm{\v{T} }}_{e}} -1 \right) -1 \end{array} \right) }^{ \frac{1}{\mathrm{\gimel }} }\\ \displaystyle +\iota { \left( \begin{array}{@{}c@{}} \displaystyle \frac{{\mathrm{\v{T} }}_{1}}{\sum _{e=1}^{2}{\mathrm{\v{T} }}_{e}} { \left( {\mathfrak{Y}}_{{\Xi }_{1}}^{IP} \right) }^{\mathrm{\gimel }}- \left( \frac{{\mathrm{\v{T} }}_{1}}{\sum _{e=1}^{2}{\mathrm{\v{T} }}_{e}} -1 \right) \\ \displaystyle + \frac{{\mathrm{\v{T} }}_{2}}{\sum _{e=1}^{2}{\mathrm{\v{T} }}_{e}} { \left( {\mathfrak{Y}}_{{\Xi }_{2}}^{IP} \right) }^{\mathrm{\gimel }}- \left( \frac{{\mathrm{\v{T} }}_{2}}{\sum _{e=1}^{2}{\mathrm{\v{T} }}_{e}} -1 \right) -1 \end{array} \right) }^{ \frac{1}{\mathrm{\gimel }} },\\ \displaystyle -1+{ \left( \begin{array}{@{}c@{}} \displaystyle \frac{{\mathrm{\v{T} }}_{1}}{\sum _{e=1}^{2}{\mathrm{\v{T} }}_{e}} { \left( 1+{\mathfrak{Y}}_{{\Xi }_{1}}^{RN} \right) }^{\mathrm{\gimel }}- \left( \frac{{\mathrm{\v{T} }}_{1}}{\sum _{e=1}^{2}{\mathrm{\v{T} }}_{e}} -1 \right) \\ \displaystyle + \frac{{\mathrm{\v{T} }}_{2}}{\sum _{e=1}^{2}{\mathrm{\v{T} }}_{e}} { \left( 1+{\mathfrak{Y}}_{{\Xi }_{2}}^{RN} \right) }^{\mathrm{\gimel }}- \left( \frac{{\mathrm{\v{T} }}_{2}}{\sum _{e=1}^{2}{\mathrm{\v{T} }}_{e}} -1 \right) -1 \end{array} \right) }^{ \frac{1}{\mathrm{\gimel }} } \end{array}\\ \displaystyle +\iota \left( -1+{ \left( \begin{array}{@{}c@{}} \displaystyle \frac{{\mathrm{\v{T} }}_{1}}{\sum _{e=1}^{2}{\mathrm{\v{T} }}_{e}} { \left( 1+{\mathfrak{Y}}_{{\Xi }_{1}}^{IN} \right) }^{\mathrm{\gimel }}- \left( \frac{{\mathrm{\v{T} }}_{1}}{\sum _{e=1}^{2}{\mathrm{\v{T} }}_{e}} -1 \right) \\ \displaystyle + \frac{{\mathrm{\v{T} }}_{2}}{\sum _{e=1}^{2}{\mathrm{\v{T} }}_{e}} { \left( 1+{\mathfrak{Y}}_{{\Xi }_{2}}^{IN} \right) }^{\mathrm{\gimel }}- \left( \frac{{\mathrm{\v{T} }}_{2}}{\sum _{e=1}^{2}{\mathrm{\v{T} }}_{e}} -1 \right) -1 \end{array} \right) }^{ \frac{1}{\mathrm{\gimel }} } \right) \end{array} \right) \nonumber\\\displaystyle = \left( \begin{array}{@{}c@{}} \displaystyle \begin{array}{@{}c@{}} \displaystyle { \left( \frac{{\mathrm{\v{T} }}_{1}}{\sum _{e=1}^{2}{\mathrm{\v{T} }}_{e}} { \left( {\mathfrak{Y}}_{{\Xi }_{1}}^{RP} \right) }^{\mathrm{\gimel }}+ \frac{{\mathrm{\v{T} }}_{2}}{\sum _{e=1}^{2}{\mathrm{\v{T} }}_{e}} { \left( {\mathfrak{Y}}_{{\Xi }_{2}}^{RP} \right) }^{\mathrm{\gimel }}- \frac{{\mathrm{\v{T} }}_{1}}{\sum _{e=1}^{2}{\mathrm{\v{T} }}_{e}} - \frac{{\mathrm{\v{T} }}_{2}}{\sum _{e=1}^{2}{\mathrm{\v{T} }}_{e}} +1 \right) }^{ \frac{1}{\mathrm{\gimel }} }\\ \displaystyle +\iota { \left( \frac{{\mathrm{\v{T} }}_{1}}{\sum _{e=1}^{2}{\mathrm{\v{T} }}_{e}} { \left( {\mathfrak{Y}}_{{\Xi }_{1}}^{IP} \right) }^{\mathrm{\gimel }}+ \frac{{\mathrm{\v{T} }}_{2}}{\sum _{e=1}^{2}{\mathrm{\v{T} }}_{e}} { \left( {\mathfrak{Y}}_{{\Xi }_{2}}^{IP} \right) }^{\mathrm{\gimel }}- \frac{{\mathrm{\v{T} }}_{1}}{\sum _{e=1}^{2}{\mathrm{\v{T} }}_{e}} - \frac{{\mathrm{\v{T} }}_{2}}{\sum _{e=1}^{2}{\mathrm{\v{T} }}_{e}} +1 \right) }^{ \frac{1}{\mathrm{\gimel }} },\\ \displaystyle -1+{ \left( \begin{array}{@{}c@{}} \displaystyle \frac{{\mathrm{\v{T} }}_{1}}{\sum _{e=1}^{2}{\mathrm{\v{T} }}_{e}} { \left( 1+{\mathfrak{Y}}_{{\Xi }_{1}}^{RN} \right) }^{\mathrm{\gimel }}+ \frac{{\mathrm{\v{T} }}_{2}}{\sum _{e=1}^{2}{\mathrm{\v{T} }}_{e}} { \left( 1+{\mathfrak{Y}}_{{\Xi }_{2}}^{RN} \right) }^{\mathrm{\gimel }}\vskip{1pc}\\ \displaystyle - \frac{{\mathrm{\v{T} }}_{1}}{\sum _{e=1}^{2}{\mathrm{\v{T} }}_{e}} - \frac{{\mathrm{\v{T} }}_{2}}{\sum _{e=1}^{2}{\mathrm{\v{T} }}_{e}} +1 \end{array} \right) }^{ \frac{1}{\mathrm{\gimel }} } \end{array}\\ \displaystyle +\iota \left( -1+{ \left( \begin{array}{@{}c@{}} \displaystyle \frac{{\mathrm{\v{T} }}_{1}}{\sum _{e=1}^{2}{\mathrm{\v{T} }}_{e}} { \left( 1+{\mathfrak{Y}}_{{\Xi }_{1}}^{IN} \right) }^{\mathrm{\gimel }}+ \frac{{\mathrm{\v{T} }}_{2}}{\sum _{e=1}^{2}{\mathrm{\v{T} }}_{e}} { \left( 1+{\mathfrak{Y}}_{{\Xi }_{2}}^{IN} \right) }^{\mathrm{\gimel }}\vskip{1pc}\\ \displaystyle - \frac{{\mathrm{\v{T} }}_{1}}{\sum _{e=1}^{2}{\mathrm{\v{T} }}_{e}} - \frac{{\mathrm{\v{T} }}_{2}}{\sum _{e=1}^{2}{\mathrm{\v{T} }}_{e}} +1 \end{array} \right) }^{ \frac{1}{\mathrm{\gimel }} } \right) \end{array} \right) \nonumber\\\displaystyle = \left( \begin{array}{@{}c@{}} \displaystyle { \left( \sum _{e=1}^{2} \frac{{\mathrm{\v{T} }}_{e}}{\sum _{e=1}^{2}{\mathrm{\v{T} }}_{e}} { \left( {\mathfrak{Y}}_{{\Xi }_{e}}^{RP} \right) }^{\mathrm{\gimel }}-\sum _{e=1}^{2} \frac{{\mathrm{\v{T} }}_{e}}{\sum _{e=1}^{2}{\mathrm{\v{T} }}_{e}} +1 \right) }^{ \frac{1}{\mathrm{\gimel }} }\\ \displaystyle +\iota { \left( \sum _{e=1}^{2} \frac{{\mathrm{\v{T} }}_{e}}{\sum _{e=1}^{2}{\mathrm{\v{T} }}_{e}} { \left( {\mathfrak{Y}}_{{\Xi }_{e}}^{IP} \right) }^{\mathrm{\gimel }}-\sum _{e=1}^{2} \frac{{\mathrm{\v{T} }}_{e}}{\sum _{e=1}^{2}{\mathrm{\v{T} }}_{e}} +1 \right) }^{ \frac{1}{\mathrm{\gimel }} },\\ \displaystyle -1+{ \left( \sum _{e=1}^{2} \frac{{\mathrm{\v{T} }}_{e}}{\sum _{e=1}^{2}{\mathrm{\v{T} }}_{e}} { \left( 1+{\mathfrak{Y}}_{{\Xi }_{e}}^{RN} \right) }^{\mathrm{\gimel }}-\sum _{e=1}^{2} \frac{{\mathrm{\v{T} }}_{e}}{\sum _{e=1}^{2}{\mathrm{\v{T} }}_{e}} +1 \right) }^{ \frac{1}{\mathrm{\gimel }} }\\ \displaystyle +\iota \left( -1+{ \left( \sum _{e=1}^{2} \frac{{\mathrm{\v{T} }}_{e}}{\sum _{e=1}^{2}{\mathrm{\v{T} }}_{e}} { \left( 1+{\mathfrak{Y}}_{{\Xi }_{e}}^{IN} \right) }^{\mathrm{\gimel }}-\sum _{e=1}^{2} \frac{{\mathrm{\v{T} }}_{e}}{\sum _{e=1}^{2}{\mathrm{\v{T} }}_{e}} +1 \right) }^{ \frac{1}{\mathrm{\gimel }} } \right) \end{array} \right) \end{eqnarray*}

Thus, Eq. (14) is valid for $\check {\mathrm{k}}=2$. Take Eq. (14) is additionally valid for $\check {\mathrm{k}}=\Gamma $, then

\begin{eqnarray*}BCFSSPRWG \left( {\Xi }_{1},{\Xi }_{2},\ldots ,{\Xi }_{\Gamma } \right) \nonumber\\\displaystyle = \left( \begin{array}{@{}c@{}} \displaystyle { \left( \sum _{e=1}^{\Gamma } \frac{{\mathrm{\v{T} }}_{e}}{\sum _{e=1}^{\Gamma }{\mathrm{\v{T} }}_{e}} { \left( {\mathfrak{Y}}_{{\Xi }_{e}}^{RP} \right) }^{\mathrm{\gimel }}-\sum _{e=1}^{\Gamma } \frac{{\mathrm{\v{T} }}_{e}}{\sum _{e=1}^{\Gamma }{\mathrm{\v{T} }}_{e}} +1 \right) }^{ \frac{1}{\mathrm{\gimel }} }\\ \displaystyle +\iota { \left( \sum _{e=1}^{\Gamma } \frac{{\mathrm{\v{T} }}_{e}}{\sum _{e=1}^{\Gamma }{\mathrm{\v{T} }}_{e}} { \left( {\mathfrak{Y}}_{{\Xi }_{e}}^{IP} \right) }^{\mathrm{\gimel }}-\sum _{e=1}^{\Gamma } \frac{{\mathrm{\v{T} }}_{e}}{\sum _{e=1}^{\Gamma }{\mathrm{\v{T} }}_{e}} +1 \right) }^{ \frac{1}{\mathrm{\gimel }} },\\ \displaystyle -1+{ \left( \sum _{e=1}^{\Gamma } \frac{{\mathrm{\v{T} }}_{e}}{\sum _{e=1}^{\Gamma }{\mathrm{\v{T} }}_{e}} { \left( 1+{\mathfrak{Y}}_{{\Xi }_{e}}^{RN} \right) }^{\mathrm{\gimel }}-\sum _{e=1}^{\Gamma } \frac{{\mathrm{\v{T} }}_{e}}{\sum _{e=1}^{\Gamma }{\mathrm{\v{T} }}_{e}} +1 \right) }^{ \frac{1}{\mathrm{\gimel }} }\\ \displaystyle +\iota \left( -1+{ \left( \sum _{e=1}^{\Gamma } \frac{{\mathrm{\v{T} }}_{e}}{\sum _{e=1}^{\Gamma }{\mathrm{\v{T} }}_{e}} { \left( 1+{\mathfrak{Y}}_{{\Xi }_{e}}^{IN} \right) }^{\mathrm{\gimel }}-\sum _{e=1}^{\Gamma } \frac{{\mathrm{\v{T} }}_{e}}{\sum _{e=1}^{\Gamma }{\mathrm{\v{T} }}_{e}} +1 \right) }^{ \frac{1}{\mathrm{\gimel }} } \right) \end{array} \right) \end{eqnarray*}

Next take $\check {\mathrm{k}}=\Gamma +1$, then

\begin{eqnarray*}BCFSSPRWG \left( {\Xi }_{1},{\Xi }_{2},\ldots ,{\Xi }_{\Gamma },{\Xi }_{\Gamma +1} \right) =BCFSSPWG \left( {\Xi }_{1},{\Xi }_{2},\ldots ,{\Xi }_{\Gamma } \right) \nonumber\\\displaystyle {\otimes }_{\mathbb{SS}}{\Xi }_{\Gamma +1}= \left( \begin{array}{@{}c@{}} \displaystyle { \left( \sum _{e=1}^{\Gamma } \frac{{\mathrm{\v{T} }}_{e}}{\sum _{e=1}^{\Gamma }{\mathrm{\v{T} }}_{e}} { \left( {\mathfrak{Y}}_{{\Xi }_{e}}^{RP} \right) }^{\mathrm{\gimel }}-\sum _{e=1}^{\Gamma } \frac{{\mathrm{\v{T} }}_{e}}{\sum _{e=1}^{\Gamma }{\mathrm{\v{T} }}_{e}} +1 \right) }^{ \frac{1}{\mathrm{\gimel }} }\\ \displaystyle +\iota { \left( \sum _{e=1}^{\Gamma } \frac{{\mathrm{\v{T} }}_{e}}{\sum _{e=1}^{\Gamma }{\mathrm{\v{T} }}_{e}} { \left( {\mathfrak{Y}}_{{\Xi }_{e}}^{IP} \right) }^{\mathrm{\gimel }}-\sum _{e=1}^{\Gamma } \frac{{\mathrm{\v{T} }}_{e}}{\sum _{e=1}^{\Gamma }{\mathrm{\v{T} }}_{e}} +1 \right) }^{ \frac{1}{\mathrm{\gimel }} },\\ \displaystyle -1+{ \left( \sum _{e=1}^{\Gamma } \frac{{\mathrm{\v{T} }}_{e}}{\sum _{e=1}^{\Gamma }{\mathrm{\v{T} }}_{e}} { \left( 1+{\mathfrak{Y}}_{{\Xi }_{e}}^{RN} \right) }^{\mathrm{\gimel }}-\sum _{e=1}^{\Gamma } \frac{{\mathrm{\v{T} }}_{e}}{\sum _{e=1}^{\Gamma }{\mathrm{\v{T} }}_{e}} +1 \right) }^{ \frac{1}{\mathrm{\gimel }} }\\ \displaystyle +\iota \left( -1+{ \left( \sum _{e=1}^{\Gamma } \frac{{\mathrm{\v{T} }}_{e}}{\sum _{e=1}^{\Gamma }{\mathrm{\v{T} }}_{e}} { \left( 1+{\mathfrak{Y}}_{{\Xi }_{e}}^{IN} \right) }^{\mathrm{\gimel }}-\sum _{e=1}^{\Gamma } \frac{{\mathrm{\v{T} }}_{e}}{\sum _{e=1}^{\Gamma }{\mathrm{\v{T} }}_{e}} +1 \right) }^{ \frac{1}{\mathrm{\gimel }} } \right) \end{array} \right) \nonumber\\\displaystyle {\otimes }_{\mathbb{SS}} \left( \begin{array}{@{}c@{}} \displaystyle { \left( \frac{{\mathrm{\v{T} }}_{\Gamma +1}}{\sum _{e=1}^{\Gamma +1}{\mathrm{\v{T} }}_{e}} { \left( {\mathfrak{Y}}_{{\Xi }_{\Gamma +1}}^{RP} \right) }^{\mathrm{\gimel }}- \left( \frac{{\mathrm{\v{T} }}_{\Gamma +1}}{\sum _{e=1}^{\Gamma +1}{\mathrm{\v{T} }}_{e}} -1 \right) \right) }^{ \frac{1}{\mathrm{\gimel }} }\\ \displaystyle +\iota { \left( \frac{{\mathrm{\v{T} }}_{\Gamma +1}}{\sum _{e=1}^{\Gamma +1}{\mathrm{\v{T} }}_{e}} { \left( {\mathfrak{Y}}_{{\Xi }_{\Gamma +1}}^{IP} \right) }^{\mathrm{\gimel }}- \left( \frac{{\mathrm{\v{T} }}_{\Gamma +1}}{\sum _{e=1}^{\Gamma +1}{\mathrm{\v{T} }}_{e}} -1 \right) \right) }^{ \frac{1}{\mathrm{\gimel }} },\\ \displaystyle -1+{ \left( \frac{{\mathrm{\v{T} }}_{\Gamma +1}}{\sum _{e=1}^{\Gamma +1}{\mathrm{\v{T} }}_{e}} { \left( 1+{\mathfrak{Y}}_{{\Xi }_{\Gamma +1}}^{RN} \right) }^{\mathrm{\gimel }}- \left( \frac{{\mathrm{\v{T} }}_{\Gamma +1}}{\sum _{e=1}^{\Gamma +1}{\mathrm{\v{T} }}_{e}} -1 \right) \right) }^{ \frac{1}{\mathrm{\gimel }} }\\ \displaystyle +\iota \left( -1+{ \left( \frac{{\mathrm{\v{T} }}_{\Gamma +1}}{\sum _{e=1}^{\Gamma +1}{\mathrm{\v{T} }}_{e}} { \left( 1+{\mathfrak{Y}}_{{\Xi }_{\Gamma +1}}^{IN} \right) }^{\mathrm{\gimel }}- \left( \frac{{\mathrm{\v{T} }}_{\Gamma +1}}{\sum _{e=1}^{\Gamma +1}{\mathrm{\v{T} }}_{e}} -1 \right) \right) }^{ \frac{1}{\mathrm{\gimel }} } \right) \end{array} \right) \nonumber\\\displaystyle = \left( \begin{array}{@{}c@{}} \displaystyle { \left( \sum _{e=1}^{\Gamma +1} \frac{{\mathrm{\v{T} }}_{e}}{\sum _{e=1}^{\Gamma +1}{\mathrm{\v{T} }}_{e}} { \left( {\mathfrak{Y}}_{{\Xi }_{e}}^{RP} \right) }^{\mathrm{\gimel }}-\sum _{e=1}^{\Gamma +1} \frac{{\mathrm{\v{T} }}_{e}}{\sum _{e=1}^{\Gamma +1}{\mathrm{\v{T} }}_{e}} +1 \right) }^{ \frac{1}{\mathrm{\gimel }} }\\ \displaystyle +\iota { \left( \sum _{e=1}^{\Gamma +1} \frac{{\mathrm{\v{T} }}_{e}}{\sum _{e=1}^{\Gamma +1}{\mathrm{\v{T} }}_{e}} { \left( {\mathfrak{Y}}_{{\Xi }_{e}}^{IP} \right) }^{\mathrm{\gimel }}-\sum _{e=1}^{\Gamma +1} \frac{{\mathrm{\v{T} }}_{e}}{\sum _{e=1}^{\Gamma +1}{\mathrm{\v{T} }}_{e}} +1 \right) }^{ \frac{1}{\mathrm{\gimel }} },\\ \displaystyle -1+{ \left( \sum _{e=1}^{\Gamma +1} \frac{{\mathrm{\v{T} }}_{e}}{\sum _{e=1}^{\Gamma +1}{\mathrm{\v{T} }}_{e}} { \left( 1+{\mathfrak{Y}}_{{\Xi }_{e}}^{RN} \right) }^{\mathrm{\gimel }}-\sum _{e=1}^{\Gamma +1} \frac{{\mathrm{\v{T} }}_{e}}{\sum _{e=1}^{\Gamma +1}{\mathrm{\v{T} }}_{e}} +1 \right) }^{ \frac{1}{\mathrm{\gimel }} }\\ \displaystyle +\iota \left( -1+{ \left( \sum _{e=1}^{\Gamma +1} \frac{{\mathrm{\v{T} }}_{e}}{\sum _{e=1}^{\Gamma +1}{\mathrm{\v{T} }}_{e}} { \left( 1+{\mathfrak{Y}}_{{\Xi }_{e}}^{IN} \right) }^{\mathrm{\gimel }}-\sum _{e=1}^{\Gamma +1} \frac{{\mathrm{\v{T} }}_{e}}{\sum _{e=1}^{\Gamma +1}{\mathrm{\v{T} }}_{e}} +1 \right) }^{ \frac{1}{\mathrm{\gimel }} } \right) \end{array} \right) \end{eqnarray*}

Thus, the Eq. (14) is valid for $\check {\mathrm{k}}=\Gamma +1$, this implies that Eq. (14) is valid $\forall \check {\mathrm{k}}$.

**Axiom 7: (Idempotency)** Consider ${\Xi }_{e}= \left( {\mathfrak{Y}}_{{\Xi }_{e}}^{P},{\mathfrak{Y}}_{{\Xi }_{e}}^{N} \right) = \left( {\mathfrak{Y}}_{{\Xi }_{e}}^{RP}+\iota {\mathfrak{Y}}_{{\Xi }_{e}}^{IP},{\mathfrak{Y}}_{{\Xi }_{e}}^{RN}+\iota {\mathfrak{Y}}_{{\Xi }_{e}}^{IN} \right) ,e=1,2,..,\check {\mathrm{k}}$ as a group of BCFNs, and if ${\Xi }_{e}=\Xi = \left( {\mathfrak{Y}}_{\Xi }^{P},{\mathfrak{Y}}_{\Xi }^{N} \right) = \left( {\mathfrak{Y}}_{\Xi }^{RP}+\iota {\mathfrak{Y}}_{\Xi }^{IP},{\mathfrak{Y}}_{\Xi }^{RN}+\iota {\mathfrak{Y}}_{\Xi }^{IN} \right) \forall e$, then

\begin{eqnarray*}BCFSSPRWG \left( {\Xi }_{1},{\Xi }_{2},\ldots ,{\Xi }_{\check {\mathrm{k}}} \right) =\Xi \end{eqnarray*}

**Axiom 8: (Monotonicity)** Consider ${\Xi }_{e}= \left( {\mathfrak{Y}}_{{\Xi }_{e}}^{P},{\mathfrak{Y}}_{{\Xi }_{e}}^{N} \right) = \left( {\mathfrak{Y}}_{{\Xi }_{e}}^{RP}+\iota {\mathfrak{Y}}_{{\Xi }_{e}}^{IP},{\mathfrak{Y}}_{{\Xi }_{e}}^{RN}+\iota {\mathfrak{Y}}_{{\Xi }_{e}}^{IN} \right) ,$ and ${\Xi }_{e}^{{}^{{^{\prime}}}}= \left( {\mathfrak{Y}}_{{\Xi }_{e}^{{}^{{^{\prime}}}}}^{P},{\mathfrak{Y}}_{{\Xi }_{e}^{{}^{{^{\prime}}}}}^{N} \right) = \left( {\mathfrak{Y}}_{{\Xi }_{e}^{{}^{{^{\prime}}}}}^{RP}+\iota {\mathfrak{Y}}_{{\Xi }_{e}^{{}^{{^{\prime}}}}}^{IP},{\mathfrak{Y}}_{{\Xi }_{e}^{{}^{{^{\prime}}}}}^{RN}+\iota {\mathfrak{Y}}_{{\Xi }_{e}^{{}^{{^{\prime}}}}}^{IN} \right) ,e=1,2,..,\check {\mathrm{k}}$ as two groups of BCFNs, and if ${\mathfrak{Y}}_{{\Xi }_{e}}^{RP}\leq {\mathfrak{Y}}_{{\Xi }_{e}^{{}^{{^{\prime}}}}}^{RP}$, ${\mathfrak{Y}}_{{\Xi }_{e}}^{IP}\leq {\mathfrak{Y}}_{{\Xi }_{e}^{{}^{{^{\prime}}}}}^{IP}$, ${\mathfrak{Y}}_{{\Xi }_{e}}^{RN}\leq {\mathfrak{Y}}_{{\Xi }_{e}^{{}^{{^{\prime}}}}}^{RN}$, and ${\mathfrak{Y}}_{{\Xi }_{e}}^{IN}\leq {\mathfrak{Y}}_{{\Xi }_{e}^{{}^{{^{\prime}}}}}^{IN}$ , then

\begin{eqnarray*}BCFSSPRWG \left( {\Xi }_{1},{\Xi }_{2},\ldots ,{\Xi }_{\check {\mathrm{k}}} \right) \leq BCFSSPRWG \left( {\Xi }_{1}^{{}^{{^{\prime}}}},{\Xi }_{2}^{{}^{{^{\prime}}}},\ldots ,{\Xi }_{\check {\mathrm{ k}}}^{{}^{{^{\prime}}}} \right) \end{eqnarray*}

**Axiom 9: (Boundedness)** Consider ${\Xi }_{e}= \left( {\mathfrak{Y}}_{{\Xi }_{e}}^{P},{\mathfrak{Y}}_{{\Xi }_{e}}^{N} \right) = \left( {\mathfrak{Y}}_{{\Xi }_{e}}^{RP}+\iota {\mathfrak{Y}}_{{\Xi }_{e}}^{IP},{\mathfrak{Y}}_{{\Xi }_{e}}^{RN}+\iota {\mathfrak{Y}}_{{\Xi }_{e}}^{IN} \right) ,e=1,2,..,\check {\mathrm{k}}$ as a group of BCFNs and if ${\Xi }^{-}= \left( {\scriptsize \begin{array}{@{}c@{}} \displaystyle {\min }_{e} \left\{ {\mathfrak{Y}}_{{\Xi }_{e}}^{RP} \right\} +\iota {\min }_{e} \left\{ {\mathfrak{Y}}_{{\Xi }_{e}}^{IP} \right\} ,\\ \displaystyle {\max }_{e} \left\{ {\mathfrak{Y}}_{{\Xi }_{e}}^{RN} \right\} +\iota {\max }_{e} \left\{ {\mathfrak{Y}}_{{\Xi }_{e}}^{IN} \right\} \end{array}} \right) ,$ and ${\Xi }^{+}= \left( {\scriptsize \begin{array}{@{}c@{}} \displaystyle {\max }_{e} \left\{ {\mathfrak{Y}}_{{\Xi }_{e}}^{RP} \right\} +\iota {\max }_{e} \left\{ {\mathfrak{Y}}_{{\Xi }_{e}}^{IP} \right\} ,\\ \displaystyle {\min }_{e} \left\{ {\mathfrak{Y}}_{{\Xi }_{e}}^{RN} \right\} +\iota {\min }_{e} \left\{ {\mathfrak{Y}}_{{\Xi }_{e}}^{IN} \right\} \end{array}} \right) $, then

\begin{eqnarray*}{\Xi }^{-}\leq BCFSSPRWG \left( {\Xi }_{1},{\Xi }_{2},\ldots ,{\Xi }_{\check {\mathrm{k}}} \right) \leq {\Xi }^{+} \end{eqnarray*}

**Definition 11:** Consider ${\Xi }_{e}= \left( {\mathfrak{Y}}_{{\Xi }_{e}}^{P},{\mathfrak{Y}}_{{\Xi }_{e}}^{N} \right) = \left( {\mathfrak{Y}}_{{\Xi }_{e}}^{RP}+\iota {\mathfrak{Y}}_{{\Xi }_{e}}^{IP},{\mathfrak{Y}}_{{\Xi }_{e}}^{RN}+\iota {\mathfrak{Y}}_{{\Xi }_{e}}^{IN} \right) ,e=1,2,..,\check {\mathrm{k}}$ as a group of BCFNs, then

(15) \begin{eqnarray*}BCFSSPROWG \left( {\Xi }_{1},{\Xi }_{2},\ldots ,{\Xi }_{\check {\mathrm{k}}} \right) =\begin{array}{@{}c@{}} \displaystyle \check {\mathrm{k}}\\ \displaystyle \otimes \\ \displaystyle e=1 \end{array}{ \left( {\Xi }_{\varsigma \left( e \right) } \right) }^{ \frac{{\mathrm{\v{T} }}_{e}}{\sum _{e=1}^{\check {\mathrm{k}}}{\mathrm{\v{T} }}_{e}} }\end{eqnarray*}

is introduced as BCFSSPROWG operator, where Ť_1_ = 1, ${\mathrm{\v{T} }}_{e}={\mathop{\prod }\nolimits }_{e=1}^{\check {\mathrm{k}}-1}{\mathfrak{S}}_{\mathfrak{B}} \left( {\Xi }_{e} \right) ,e=1,2,\ldots ,\check {\mathrm{k}},{\mathfrak{S}}_{\mathfrak{B}} \left( {\Xi }_{e} \right) $ would deduce the score value of BCFN Ξ_*ȅ*_ and $ \left( \varsigma \left( 1 \right) ,\varsigma \left( 2 \right) ,..,\varsigma \left( \check {\mathrm{k}} \right) \right) $ is a permutation of $ \left( 1,2,..,\check {\mathrm{k}} \right) $ with ∀̦*e* ${\Xi }_{\varsigma \left( e-1 \right) }\geq {\Xi }_{-\varsigma \left( e \right) }$.

**Theorem 4:** Consider ${\Xi }_{e}= \left( {\mathfrak{Y}}_{{\Xi }_{e}}^{P},{\mathfrak{Y}}_{{\Xi }_{e}}^{N} \right) = \left( {\mathfrak{Y}}_{{\Xi }_{e}}^{RP}+\iota {\mathfrak{Y}}_{{\Xi }_{e}}^{IP},{\mathfrak{Y}}_{{\Xi }_{e}}^{RN}+\iota {\mathfrak{Y}}_{{\Xi }_{e}}^{IN} \right) ,e=1,2,..,\check {\mathrm{k}}$ as a group of BCFNs, then by utilizing BCFSSPROWG, we achieve the aggregated result and

(16) \begin{eqnarray*}BCFSSPROWG \left( {\Xi }_{1},{\Xi }_{2},\ldots ,{\Xi }_{\check {\mathrm{k}}} \right) = \left( \begin{array}{@{}c@{}} \displaystyle { \left( \sum _{e=1}^{\check {\mathrm{k}}} \frac{{\mathrm{\v{T} }}_{e}}{\sum _{e=1}^{\check {\mathrm{k}}}{\mathrm{\v{T} }}_{e}} { \left( {\mathfrak{Y}}_{{\Xi }_{\varsigma \left( e \right) }}^{RP} \right) }^{\mathrm{\gimel }} \right) }^{ \frac{1}{\mathrm{\gimel }} }\\ \displaystyle +\iota { \left( \sum _{e=1}^{\check {\mathrm{k}}} \frac{{\mathrm{\v{T} }}_{e}}{\sum _{e=1}^{\check {\mathrm{k}}}{\mathrm{\v{T} }}_{e}} { \left( {\mathfrak{Y}}_{{\Xi }_{\varsigma \left( e \right) }}^{IP} \right) }^{\mathrm{\gimel }} \right) }^{ \frac{1}{\mathrm{\gimel }} },\\ \displaystyle -1+{ \left( \sum _{e=1}^{\check {\mathrm{k}}} \frac{{\mathrm{\v{T} }}_{e}}{\sum _{e=1}^{\check {\mathrm{k}}}{\mathrm{\v{T} }}_{e}} { \left( 1+{\mathfrak{Y}}_{{\Xi }_{\varsigma \left( e \right) }}^{RN} \right) }^{\mathrm{\gimel }} \right) }^{ \frac{1}{\mathrm{\gimel }} }\\ \displaystyle +\iota \left( -1+{ \left( \sum _{e=1}^{\check {\mathrm{k}}} \frac{{\mathrm{\v{T} }}_{e}}{\sum _{e=1}^{\check {\mathrm{k}}}{\mathrm{\v{T} }}_{e}} { \left( 1+{\mathfrak{Y}}_{{\Xi }_{\varsigma \left( e \right) }}^{IN} \right) }^{\mathrm{\gimel }} \right) }^{ \frac{1}{\mathrm{\gimel }} } \right) \end{array} \right) \end{eqnarray*}

**Axiom 10: (Idempotency)** Consider ${\Xi }_{e}= \left( {\mathfrak{Y}}_{{\Xi }_{e}}^{P},{\mathfrak{Y}}_{{\Xi }_{e}}^{N} \right) = \left( {\mathfrak{Y}}_{{\Xi }_{e}}^{RP}+\iota {\mathfrak{Y}}_{{\Xi }_{e}}^{IP},{\mathfrak{Y}}_{{\Xi }_{e}}^{RN}+\iota {\mathfrak{Y}}_{{\Xi }_{e}}^{IN} \right) ,e=1,2,..,\check {\mathrm{k}}$ as a group of BCFNs, and if ${\Xi }_{e}=\Xi = \left( {\mathfrak{Y}}_{\Xi }^{P},{\mathfrak{Y}}_{\Xi }^{N} \right) = \left( {\mathfrak{Y}}_{\Xi }^{RP}+\iota {\mathfrak{Y}}_{\Xi }^{IP},{\mathfrak{Y}}_{\Xi }^{RN}+\iota {\mathfrak{Y}}_{\Xi }^{IN} \right) \forall e$, then

\begin{eqnarray*}BCFSSPROWG \left( {\Xi }_{1},{\Xi }_{2},\ldots ,{\Xi }_{\check {\mathrm{k}}} \right) =\Xi \end{eqnarray*}

**Axiom 11: (Monotonicity)** Consider ${\Xi }_{e}= \left( {\mathfrak{Y}}_{{\Xi }_{e}}^{P},{\mathfrak{Y}}_{{\Xi }_{e}}^{N} \right) = \left( {\mathfrak{Y}}_{{\Xi }_{e}}^{RP}+\iota {\mathfrak{Y}}_{{\Xi }_{e}}^{IP},{\mathfrak{Y}}_{{\Xi }_{e}}^{RN}+\iota {\mathfrak{Y}}_{{\Xi }_{e}}^{IN} \right) ,$ and ${\Xi }_{e}^{{}^{{^{\prime}}}}= \left( {\mathfrak{Y}}_{{\Xi }_{e}^{{}^{{^{\prime}}}}}^{P},{\mathfrak{Y}}_{{\Xi }_{e}^{{}^{{^{\prime}}}}}^{N} \right) = \left( {\mathfrak{Y}}_{{\Xi }_{e}^{{}^{{^{\prime}}}}}^{RP}+\iota {\mathfrak{Y}}_{{\Xi }_{e}^{{}^{{^{\prime}}}}}^{IP},{\mathfrak{Y}}_{{\Xi }_{e}^{{}^{{^{\prime}}}}}^{RN}+\iota {\mathfrak{Y}}_{{\Xi }_{e}^{{}^{{^{\prime}}}}}^{IN} \right) ,e=1,2,..,\check {\mathrm{k}}$ as two groups of BCFNs, and if ${\mathfrak{Y}}_{{\Xi }_{e}}^{RP}\leq {\mathfrak{Y}}_{{\Xi }_{e}^{{}^{{^{\prime}}}}}^{RP}$, ${\mathfrak{Y}}_{{\Xi }_{e}}^{IP}\leq {\mathfrak{Y}}_{{\Xi }_{e}^{{}^{{^{\prime}}}}}^{IP}$, ${\mathfrak{Y}}_{{\Xi }_{e}}^{RN}\leq {\mathfrak{Y}}_{{\Xi }_{e}^{{}^{{^{\prime}}}}}^{RN}$, and ${\mathfrak{Y}}_{{\Xi }_{e}}^{IN}\leq {\mathfrak{Y}}_{{\Xi }_{e}^{{}^{{^{\prime}}}}}^{IN}$ , then

\begin{eqnarray*}BCFSSPROWG \left( {\Xi }_{1},{\Xi }_{2},\ldots ,{\Xi }_{\check {\mathrm{k}}} \right) \leq BCFSSPROWG \left( {\Xi }_{1}^{{}^{{^{\prime}}}},{\Xi }_{2}^{{}^{{^{\prime}}}},\ldots ,{\Xi }_{\check {\mathrm{ k}}}^{{}^{{^{\prime}}}} \right) \end{eqnarray*}

**Axiom 12: (Boundedness)** Consider ${\Xi }_{e}= \left( {\mathfrak{Y}}_{{\Xi }_{e}}^{P},{\mathfrak{Y}}_{{\Xi }_{e}}^{N} \right) = \left( {\mathfrak{Y}}_{{\Xi }_{e}}^{RP}+\iota {\mathfrak{Y}}_{{\Xi }_{e}}^{IP},{\mathfrak{Y}}_{{\Xi }_{e}}^{RN}+\iota {\mathfrak{Y}}_{{\Xi }_{e}}^{IN} \right) ,e=1,2,..,\check {\mathrm{k}}$ as a group of BCFNs and if ${\Xi }^{-}= \left( {\scriptsize \begin{array}{@{}c@{}} \displaystyle {\min }_{e} \left\{ {\mathfrak{Y}}_{{\Xi }_{e}}^{RP} \right\} +\iota {\min }_{e} \left\{ {\mathfrak{Y}}_{{\Xi }_{e}}^{IP} \right\} ,\\ \displaystyle {\max }_{e} \left\{ {\mathfrak{Y}}_{{\Xi }_{e}}^{RN} \right\} +\iota {\max }_{e} \left\{ {\mathfrak{Y}}_{{\Xi }_{e}}^{IN} \right\} \end{array}} \right) ,$ and ${\Xi }^{+}= \left( {\scriptsize \begin{array}{@{}c@{}} \displaystyle {\max }_{e} \left\{ {\mathfrak{Y}}_{{\Xi }_{e}}^{RP} \right\} +\iota {\max }_{e} \left\{ {\mathfrak{Y}}_{{\Xi }_{e}}^{IP} \right\} ,\\ \displaystyle {\min }_{e} \left\{ {\mathfrak{Y}}_{{\Xi }_{e}}^{RN} \right\} +\iota {\min }_{e} \left\{ {\mathfrak{Y}}_{{\Xi }_{e}}^{IN} \right\} \end{array}} \right) $, then

\begin{eqnarray*}{\Xi }^{-}\leq BCFSSPROWG \left( {\Xi }_{1},{\Xi }_{2},\ldots ,{\Xi }_{\check {\mathrm{k}}} \right) \leq {\Xi }^{+} \end{eqnarray*}

## Application

Disorders of the mind can be sporadic or continuous. Additionally, they have an impact on a person’s capacity for daily living and interpersonal relationships. There are ways to enhance general mental well-being, but some diseases are more severe and may call for expert help. The four prevalent mental disorders are listed below:

**1. Psychotics disorders**: The ability to distinguish between what is real and what is not may be impaired in those with psychotic illnesses. Various mental illnesses alter a person’s perception of reality. According to scientists, the development of psychotic diseases may be influenced by some viruses, issues with particular brain circuits, excessive stress or trauma, and several drug misuse behaviors. Schizophrenic disorder, schizophrenic tendencies, acute psychotic illness, hallucinations, and drug psychotic disorder are the most prevalent psychotic disorders.

**2. Dementia**: Dementia is a word that encompasses a variety of distinct brain disorders, despite being wrongly thought of as a physical entity. People with dementia-related illnesses may undergo cognitive deficits, which are frequently sufficient to impact everyday functioning and independence. Brain damage contributes to sixty to eighty percent of cases of dementia, even though this category comprises a variety of illnesses. It gradually impairs cognition and cognitive capacity and ultimately renders one incapable of doing even basic duties. Parkinsonism, Pick’s disease, hereditary chorea, and Korsakoff syndrome are additional types of dementia.

**3. Mood disorders** One in ten persons is thought to have a type of mood illness. While mood fluctuations are common, people with psychological disorders suffer more serious and chronic symptoms that might affect their daily activities. People may continually feel depressed, nervous, or “empty,” as well as have low self-confidence, impaired judgment, low energy, and other depressed mood depending on the individual disease. Treatment options for mood disorders include psychotherapy, medications, and self-care. Major depressive disorder, mild chronic depression, manic depression, and stimulant mood disorders are the most prevalent mood disorders.

**4. Anxiety disorders**: About forty million persons aged eighteen and over in the U.S. suffer from the most prevalent group of mental health conditions. People who are affected by anxiety frequently and alarmingly feel scared and uncomfortable. While many people may feel these emotions, for example, during a hiring process or communication skills engagement, individuals who suffer from anxiety disorders experience them frequently and in normally non-stressful situations. Additionally, an anxiety attack may linger for 6 months. The term “anxiety” actually refers to a wide range of distinct conditions, including neurotic disorders, panic attacks, agoraphobia, battle fatigue, and social phobia.

For the identification and prioritization of these types of mental disorders, we deduced an approach of DM depending on the introduced SS AOs under the BCF information as follows.

### Method of DM

Hold a group of $\check {\mathrm{k}}$ number of alternatives $\Lambda = \left\{ {\Lambda }_{1},{\Lambda }_{2},\ldots ,{\Lambda }_{\check {\mathrm{k}}} \right\} $ and J the number of attributes $\mathfrak{C}= \left\{ {\mathfrak{C}}_{1},{\mathfrak{C}}_{2},\ldots ,{\mathfrak{C}}_{\mathrm{J}} \right\} $ which holds the condition of prioritization that is ℭ_1_ > ℭ_2_ > … > ℭ_J_. This means that if $e< \breve {\mathrm{g}}$, then ℭ_*ȅ*_ is prior than ${\mathfrak{C}}_{\breve {\mathrm{g}}}$. Consider ${D}_{\mathfrak{M}}={ \left( {\Xi }_{e\breve {\mathrm{g}}} \right) }_{\check {\mathrm{k}}\times \mathrm{J}}={ \left( {\mathfrak{Y}}_{{\Xi }_{e\breve {\mathrm{ g}}}}^{P},{\mathfrak{Y}}_{{\Xi }_{e\breve {\mathrm{ g}}}}^{N} \right) }_{\check {\mathrm{k}}\times \mathrm{J}}={ \left( {\mathfrak{Y}}_{{\Xi }_{e\breve {\mathrm{ g}}}}^{RP}+\iota {\mathfrak{Y}}_{{\Xi }_{e\breve {\mathrm{ g}}}}^{IP},{\mathfrak{Y}}_{{\Xi }_{e\breve {\mathrm{ g}}}}^{RN}+\iota {\mathfrak{Y}}_{{\Xi }_{e\breve {\mathrm{ g}}}}^{IN} \right) }_{\check {\mathrm{k}}\times \mathrm{J}}$ as a decision matrix, in the setting of BCFS, where ${\mathfrak{Y}}_{{\Xi }_{e\breve {\mathrm{ g}}}}^{P}$ classifies positive truth degree and ${\mathfrak{Y}}_{{\Xi }_{e\breve {\mathrm{ g}}}}^{N}$ classifies negative truth degree and ${\mathfrak{Y}}_{{\Xi }_{e\breve {\mathrm{ g}}}}^{RP},{\mathfrak{Y}}_{{\Xi }_{e\breve {\mathrm{ g}}}}^{IP}\in \left[ 0,1 \right] $ and ${\mathfrak{Y}}_{{\Xi }_{e\breve {\mathrm{ g}}}}^{RN},{\mathfrak{Y}}_{{\Xi }_{e\breve {\mathrm{ g}}}}^{IN}\in \left[ -1,0 \right] $. To handle this BCF decision matrix we investigate the following approach of DM

**Step 1:** There are numerous circumstances where the attributes can be cost or benefit kind. Thus, in such sort of circumstances, the decision matrix should be standardized by employing the underneath formula.

(17) \begin{eqnarray*}{D}_{\mathfrak{M}}^{\mathfrak{N}}= \left\{ \begin{array}{@{}l@{}} \displaystyle \left( \begin{array}{@{}c@{}} \displaystyle {\mathfrak{Y}}_{{\Xi }_{e\breve {\mathrm{ g}}}}^{RP}+\iota {\mathfrak{Y}}_{{\Xi }_{e\breve {\mathrm{ g}}}}^{IP},\\ \displaystyle {\mathfrak{Y}}_{{\Xi }_{e\breve {\mathrm{ g}}}}^{RN}+\iota {\mathfrak{Y}}_{{\Xi }_{e\breve {\mathrm{ g}}}}^{IN} \end{array} \right)  benefit~kind \\ \displaystyle \left( \begin{array}{@{}c@{}} \displaystyle 1-{\mathfrak{Y}}_{{\Xi }_{e\breve {\mathrm{ g}}}}^{RP}+\iota \left( 1-{\mathfrak{Y}}_{{\Xi }_{e\breve {\mathrm{ g}}}}^{IP} \right) ,\\ \displaystyle -1-{\mathfrak{Y}}_{{\Xi }_{e\breve {\mathrm{ g}}}}^{RN}+\iota \left( -1-{\mathfrak{Y}}_{{\Xi }_{e\breve {\mathrm{ g}}}}^{IN} \right) \end{array} \right)  cost~kind  \end{array} \right. \end{eqnarray*}

**Step 2:** Derive the values of ${\mathrm{\v{T} }}_{e\breve {\mathrm{g}}} \left( e=1,2,..,\check {\mathrm{k}} \right) $ and $ \left( \breve {\mathrm{g}}=1,2,..,\mathrm{J} \right) $

(18) \begin{eqnarray*}{\mathrm{\v{T} }}_{e\breve {\mathrm{g}}}=\prod _{=1}^{\breve {\mathrm{g}}-1}{\mathfrak{S}}_{\mathfrak{B}} \left( {\Xi }_{e} \right) \end{eqnarray*}

where, $e=1,2,\ldots .,\check {\mathrm{k}}$, $\breve {\mathrm{g}}=1,2,\ldots ,\mathrm{J}$ and Ť_*ȅ*1_ = 1 for $e=1,2,\ldots .,\check {\mathrm{k}}$.

**Step 3:** Aggregate all data interpreted by the expert in the decision matrix by utilizing one of the originated operators, that is, BCFSSPRWA, BCFSSPROWA, BCFSSPRWG, and BCFSSPROWG operators

**Step 4:** For ranking order, derive the score or accuracy values of the aggregated results and choose the most superb alternative.

**Step 5:** End.

### Numerical example

A mental disorder, also known as a psychiatric disorder, is a clinically significant behavioral or psychological syndrome or pattern that occurs in an individual, and that is associated with distress or disability, or with a significantly increased risk of suffering death, pain, disability, or loss of freedom. Psychologists are professionals who specialize in the scientific study of human behavior and mental processes. They play a critical role in promoting mental health and well-being by assessing, diagnosing, and treating mental health disorders. Mental disorders have various types. In this example, we consider that a psychologist at a medical university wants to determine the most prevalent type of mental disorder. For this, the psychologist study various types of mental disorders and shortlisted the four most important and common types of mental disorders. These are Λ_1_ = *Psychotics* *disorder*, Λ_2_ = *Dementia*, Λ_3_ = *Mood* *disorder*, and Λ_4_ = *Anxiety* *disorder*. Further, the psychologist considers four attributes related to these four types of mental disorders which are ℭ_1_ = *Change* *in* *feelings*, ℭ_2_ = *Energy* *level*, ℭ_3_ = *Mood* *swings*, ℭ_4_ = *Interest* *level*. These attributes have two poles, change in feelings: good changes in feelings and bad changes in feelings, energy level: low energy and high energy, mood swings: happy and sad, interest level: low interest-level and high-interest level. Further, for more study of bipolarity, one can read the manuscript presented by Zeb et al. (2021).

Thus, the assessment values of these mental disorders are in the environment of BCFN and displayed in Table 1.

**Table 1:** The data was investigaed by the psychologist.

	ℭ_1_	ℭ_2_	ℭ_3_	ℭ_4_
Λ_1_	$ \left( \begin{array}{@{}c@{}} \displaystyle 0.451+\iota 0.56,\\ \displaystyle -0.75-\iota 0.37 \end{array} \right) $	$ \left( \begin{array}{@{}c@{}} \displaystyle 0.632+\iota 0.432,\\ \displaystyle -0.474-\iota 0.456 \end{array} \right) $	$ \left( \begin{array}{@{}c@{}} \displaystyle 0.465+\iota 0.398,\\ \displaystyle -0.781-\iota 0.298 \end{array} \right) $	$ \left( \begin{array}{@{}c@{}} \displaystyle 0.576+\iota 0.571,\\ \displaystyle -0.198-\iota 0.61 \end{array} \right) $
Λ_2_	$ \left( \begin{array}{@{}c@{}} \displaystyle 0.871+\iota 0.76,\\ \displaystyle -0.55-\iota 0.26 \end{array} \right) $	$ \left( \begin{array}{@{}c@{}} \displaystyle 0.712+\iota 0.542,\\ \displaystyle -0.363-\iota 0.345 \end{array} \right) $	$ \left( \begin{array}{@{}c@{}} \displaystyle 0.576+\iota 0.487,\\ \displaystyle -0.26-\iota 0.14 \end{array} \right) $	$ \left( \begin{array}{@{}c@{}} \displaystyle 0.91+\iota 0.642,\\ \displaystyle -0.115-\iota 0.2 \end{array} \right) $
Λ_3_	$ \left( \begin{array}{@{}c@{}} \displaystyle 0.612+\iota 0.617,\\ \displaystyle -0.551-\iota 0.361 \end{array} \right) $	$ \left( \begin{array}{@{}c@{}} \displaystyle 0.523+\iota 0.353,\\ \displaystyle -0.585-\iota 0.567 \end{array} \right) $	$ \left( \begin{array}{@{}c@{}} \displaystyle 0.365+\iota 0.29,\\ \displaystyle -0.951-\iota 0.471 \end{array} \right) $	$ \left( \begin{array}{@{}c@{}} \displaystyle 0.479+\iota 0.713,\\ \displaystyle -0.852-\iota 0.876 \end{array} \right) $
Λ_4_	$ \left( \begin{array}{@{}c@{}} \displaystyle 0.761+\iota 0.23,\\ \displaystyle -0.393-\iota 0.76 \end{array} \right) $	$ \left( \begin{array}{@{}c@{}} \displaystyle 0.434+\iota 0.564,\\ \displaystyle -0.696-\iota 0.678 \end{array} \right) $	$ \left( \begin{array}{@{}c@{}} \displaystyle 0.713+\iota 0.651,\\ \displaystyle -0.92-\iota 0.81 \end{array} \right) $	$ \left( \begin{array}{@{}c@{}} \displaystyle 0.371+\iota 0.371,\\ \displaystyle -0.687-\iota 0.27 \end{array} \right) $

In Table 1, $ \left( 0.451+\iota 0.56,-0.75-\iota 0.37 \right) $ is a BCFN, where 0.451 is the real part and 0.56 is the unreal part of the positive truth degree, −0.75 is the real part and −0.37 is the unreal part of the negative truth degree.

**Step 1:** In the above BCF decision matrix, all attributes are the benefits kind so we are ignoring this step.

**Step 2:** We derive all values of ${\mathrm{\v{T} }}_{e\breve {\mathrm{g}}}$by employing Eq. (18) and have

\begin{eqnarray*}{\mathrm{\v{T} }}_{e\breve {\mathrm{g}}}= \left[ \begin{array}{@{}cccc@{}} \displaystyle 1&\displaystyle 0.645&\displaystyle 0.235&\displaystyle 0.113\\ \displaystyle 1&\displaystyle 0.69&\displaystyle 0.449&\displaystyle 0.299\\ \displaystyle 1&\displaystyle 0.438&\displaystyle 0.25&\displaystyle 0.077\\ \displaystyle 1&\displaystyle 0.778&\displaystyle 0.187&\displaystyle 0.076 \end{array} \right] \end{eqnarray*}

**Step 3:** We aggregate all data interpreted in the BCF decision matrix by utilizing BCFSSPRWA, BCFSSPROWA, BCFSSPRWG, and BCFSSPROWG operators and the results are portrayed in Table 2.

**Table 2:** The aggregated argument of Table 1.

Operators	Λ_1_	Λ_2_	Λ_3_	Λ_4_
BCFSSPRWA	$ \left( \begin{array}{@{}c@{}} \displaystyle 0.55+\iota 0.517,\\ \displaystyle -0.438-\iota 0.38 \end{array} \right) $	$ \left( \begin{array}{@{}c@{}} \displaystyle 0.861+\iota 0.699,\\ \displaystyle -0.216-\iota 0.207 \end{array} \right) $	$ \left( \begin{array}{@{}c@{}} \displaystyle 0.573+\iota 0.578,\\ \displaystyle -0.59-\iota 0.407 \end{array} \right) $	$ \left( \begin{array}{@{}c@{}} \displaystyle 0.712+\iota 0.499,\\ \displaystyle -0.473-\iota 0.611 \end{array} \right) $
BCFSSPROWA	$ \left( \begin{array}{@{}c@{}} \displaystyle 0.586+\iota 0.532,\\ \displaystyle -0.245-\iota 0.461 \end{array} \right) $	$ \left( \begin{array}{@{}c@{}} \displaystyle 0.887+\iota 0.685,\\ \displaystyle -0.152-\iota 0.194 \end{array} \right) $	$ \left( \begin{array}{@{}c@{}} \displaystyle 0.577+\iota 0.612,\\ \displaystyle -0.588-\iota 0.414 \end{array} \right) $	$ \left( \begin{array}{@{}c@{}} \displaystyle 0.577+\iota 0.612,\\ \displaystyle -0.414-\iota 0.588 \end{array} \right) $
BCFSSPRWG	$ \left( \begin{array}{@{}c@{}} \displaystyle 0.495+\iota 0.479,\\ \displaystyle -0.721-\iota 0.426 \end{array} \right) $	$ \left( \begin{array}{@{}c@{}} \displaystyle 0.734+\iota 0.594,\\ \displaystyle -0.456-\iota 0.268 \end{array} \right) $	$ \left( \begin{array}{@{}c@{}} \displaystyle 0.51+\iota 0.415,\\ \displaystyle -0.907-\iota 0.675 \end{array} \right) $	$ \left( \begin{array}{@{}c@{}} \displaystyle 0.529+\iota 0.284,\\ \displaystyle -0.827-\iota 0.745 \end{array} \right) $
BCFSSPROWG	$ \left( \begin{array}{@{}c@{}} \displaystyle 0.556+\iota 0.495,\\ \displaystyle -0.607-\iota 0.551 \end{array} \right) $	$ \left( \begin{array}{@{}c@{}} \displaystyle 0.76+\iota 0.603,\\ \displaystyle -0.4-\iota 0.234 \end{array} \right) $	$ \left( \begin{array}{@{}c@{}} \displaystyle 0.538+\iota 0.46,\\ \displaystyle -0.867-\iota 0.768 \end{array} \right) $	$ \left( \begin{array}{@{}c@{}} \displaystyle 0.476+\iota 0.275,\\ \displaystyle -0.827-\iota 0.73 \end{array} \right) $

**Figure 1:**
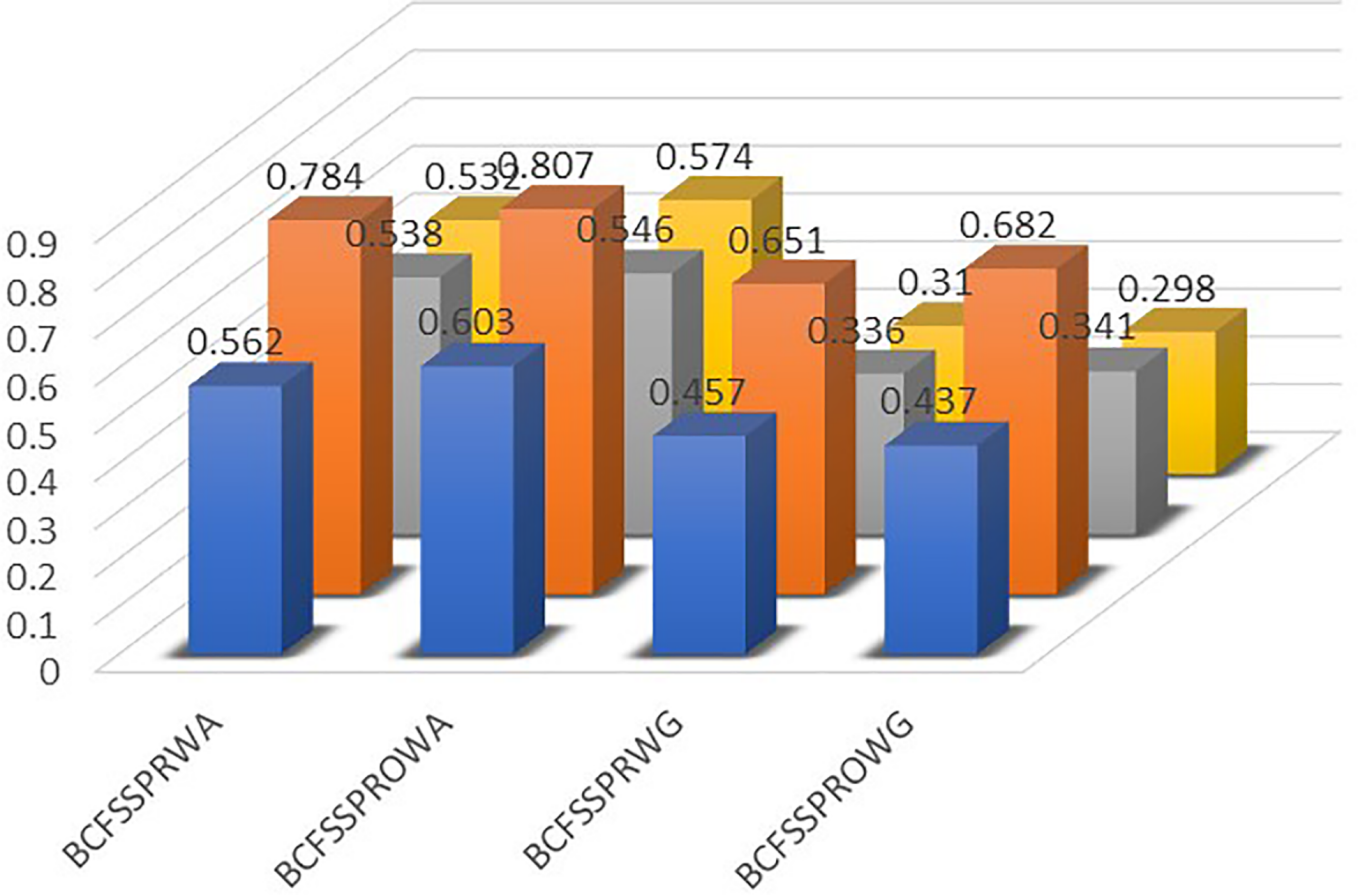
The graphical interpretation of Table 4.

**Step 4:** we drive the ranking order by utilizing the score values and the score values are determined by Eq. (2) explored in Table 3 and ranking are explored in Table 4 and graphical presentation is revealed in Fig. 1.

**Table 3:** The score value of Table 2.

Operators	${\mathfrak{S}}_{\mathfrak{B}} \left( {\Lambda }_{1} \right) $	${\mathfrak{S}}_{\mathfrak{B}} \left( {\Lambda }_{2} \right) $	${\mathfrak{S}}_{\mathfrak{B}} \left( {\Lambda }_{3} \right) $	${\mathfrak{S}}_{\mathfrak{B}} \left( {\Lambda }_{4} \right) $
BCFSSPRWA	0.562	0.784	0.538	0.532
BCFSSPROWA	0.603	0.807	0.546	0.574
BCFSSPRWG	0.457	0.651	0.336	0.31
BCFSSPROWG	0.473	0.682	0.341	0.298

The ranking shows that Λ_2_ that dementia is the most prevalent type of mental disorder.

**Step 5:** End.

### Sensitivity analysis

Here, for the sensitivity analysis, we take various values of the variable parameter ℷ in the DM procedure by employing introduced operators. By taking ℷ =  − 1, −3, −5, −10, −25, −50, we have the outcomes displayed in Table 5 and Table 6.

**Table 4:** The ranking among various types of mental disorders.

Operators	Ranking
BCFSSPRWA	Λ_2_ > Λ_1_ > Λ_3_ > Λ_4_
BCFSSPROWA	Λ_2_ > Λ_1_ > Λ_4_ > Λ_3_
BCFSSPRWG	Λ_2_ > Λ_1_ > Λ_3_ > Λ_4_
BCFSSPROWG	Λ_2_ > Λ_1_ > Λ_3_ > Λ_4_

**Table 5:** The outcomes for ℷ =  − 1,  − 3,  − 5,  − 10,  − 25,  − 50.

ℷ	Operators	${\mathfrak{S}}_{\mathfrak{B}} \left( {\Lambda }_{1} \right) $	${\mathfrak{S}}_{\mathfrak{B}} \left( {\Lambda }_{2} \right) $	${\mathfrak{S}}_{\mathfrak{B}} \left( {\Lambda }_{3} \right) $	${\mathfrak{S}}_{\mathfrak{B}} \left( {\Lambda }_{4} \right) $
−1	BCFSSPRWA	0.524	0.742	0.521	0.483
	BCFSSPROWA	0.581	0.782	0.529	0.52
	BCFSSPRWG	0.473	0.67	0.417	0.367
	BCFSSPROWG	0.51	0.708	0.425	0.362
−3	BCFSSPRWA	0.562	0.784	0.538	0.532
	BCFSSPROWA	0.603	0.807	0.546	0.574
	BCFSSPRWG	0.457	0.651	0.336	0.31
	BCFSSPROWG	0.473	0.682	0.341	0.298
−5	BCFSSPRWA	0.593	0.808	0.55	0.577
	BCFSSPROWA	0.617	0.82	0.558	0.608
	BCFSSPRWG	0.445	0.634	0.293	0.285
	BCFSSPROWG	0.45	0.66	0.305	0.271
−10	BCFSSPRWA	0.628	0.83	0.567	0.63
	BCFSSPROWA	0.638	0.835	0.574	0.646
	BCFSSPRWG	0.421	0.605	0.253	0.26
	BCFSSPROWG	0.421	0.622	0.268	0.247
−25	BCFSSPRWA	0.655	0.845	0.587	0.664
	BCFSSPROWA	0.66	0.846	0.591	0.671
	BCFSSPRWG	0.391	0.572	0.225	0.238
	BCFSSPROWG	0.392	0.58	0.233	0.23
−50	BCFSSPRWA	0.665	0.849	0.595	0.676
	BCFSSPROWA	0.668	0.85	0.597	0.679
	BCFSSPRWG	0.378	0.557	0.216	0.228
	BCFSSPROWG	0.379	0.561	0.22	0.224

**Table 6:** The ranking, based on the outcomes for ℷ =  − 1,  − 3,  − 5,  − 10,  − 25,  − 50.

ℷ	Operators	Ranking
−1	BCFSSPRWA	Λ_2_ > Λ_1_ > Λ_3_ > Λ_4_
	BCFSSPROWA	Λ_2_ > Λ_1_ > Λ_4_ > Λ_3_
	BCFSSPRWG	Λ_2_ > Λ_1_ > Λ_3_ > Λ_4_
	BCFSSPROWG	Λ_2_ > Λ_1_ > Λ_3_ > Λ_4_
−3	BCFSSPRWA	Λ_2_ > Λ_1_ > Λ_3_ > Λ_4_
	BCFSSPROWA	Λ_2_ > Λ_1_ > Λ_4_ > Λ_3_
	BCFSSPRWG	Λ_2_ > Λ_1_ > Λ_3_ > Λ_4_
	BCFSSPROWG	Λ_2_ > Λ_1_ > Λ_3_ > Λ_4_
−5	BCFSSPRWA	Λ_2_ > Λ_1_ > Λ_4_ > Λ_3_
	BCFSSPROWA	Λ_2_ > Λ_1_ > Λ_4_ > Λ_3_
	BCFSSPRWG	Λ_2_ > Λ_1_ > Λ_3_ > Λ_4_
	BCFSSPROWG	Λ_2_ > Λ_1_ > Λ_3_ > Λ_4_
−10	BCFSSPRWA	Λ_2_ > Λ_1_ > Λ_4_ > Λ_3_
	BCFSSPROWA	Λ_2_ > Λ_1_ > Λ_4_ > Λ_3_
	BCFSSPRWG	Λ_2_ > Λ_1_ > Λ_4_ > Λ_3_
	BCFSSPROWG	Λ_2_ > Λ_1_ > Λ_3_ > Λ_4_
−25	BCFSSPRWA	Λ_2_ > Λ_1_ > Λ_4_ > Λ_3_
	BCFSSPROWA	Λ_2_ > Λ_1_ > Λ_4_ > Λ_3_
	BCFSSPRWG	Λ_2_ > Λ_1_ > Λ_4_ > Λ_3_
	BCFSSPROWG	Λ_2_ > Λ_1_ > Λ_3_ > Λ_4_
−50	BCFSSPRWA	Λ_2_ > Λ_1_ > Λ_4_ > Λ_3_
	BCFSSPROWA	Λ_2_ > Λ_1_ > Λ_4_ > Λ_3_
	BCFSSPRWG	Λ_2_ > Λ_1_ > Λ_4_ > Λ_3_
	BCFSSPROWG	Λ_2_ > Λ_1_ > Λ_4_ > Λ_3_

From Tables 5 and 6, we noticed that there are minor changes in the ranking of the alternatives by employing various values of variable parameters ℷ that is −1, −3, −5, −10, −25, −50. For instance, by taking the value of variable parameter ℷ =  − 10, and employing the BCFSSPRWA operator, we have the ranking Λ_2_ > Λ_1_ > Λ_4_ > Λ_3_, which implies that Λ_2_ is the finest alternative and Λ_3_ is the worst one. But by taking the value of variable parameter ℷ =  − 3, and employing the BCFSSPRWA operator, we have the ranking Λ_2_ > Λ_1_ > Λ_3_ > Λ_4_, which implies that Λ_2_ is the finest alternative and Λ_2_ is the worst one. The finest alternative is unchanged *i.e.,* Λ_2_ in the whole interval $ \left[ -50,-1 \right] $ by employing BCFSSPRWA, BCFSSPROWA, BCFSSPRWG, and BCFSSPROWG operators. Further, we also noticed that by employing BCFSSPRWA and BCFSSPROWA operators, the values of each alternative enhancing by decreasing the value of parameter ℷ. While by employing BCFSSPRWG and BCFSSPROWG operators the score values of each alternative decrease by decreasing the value of parameter ℷ. In the DM procedure, the value of parameter ℷ can be selected according to the optimistic or pessimistic decision.

## Comparative Study

Comparative studies of the originated operator with numerous current operators are interpreted in this section for exhibiting the priority and superiority of the originated work. For doing the comparative study takes some AOs operators

• in the setting of bipolar fuzzy (BF) information such as Dombi introduced by Jana, Pal & Wang (2019), Hamacher introduced by Wei et al. (2018), and sine trigonometric devised by Riaz et al. (2022).
• in the setting of complex fuzzy (CF) information such as arithmetic AOs interpreted by Bi et al. (2019) and geometric AOs devised by Bi, Dai & Hu (2018).
• in the setting of bipolar complex fuzzy information introduced by Mahmood et al. (2022b).

Also, take the AOs based on the SS t-norm and t-conorms in other structures such as

• the SS power AOs investigated by Biswas & Deb (2021) in the structure of Pythagorean FS,
• SS prioritized AOs deduced by Tian et al. (2022) in the model of picture fuzzy information,
• SS prioritized AOs originated by Liu, Khan & Mahmood (2019) in the model of neutrosophic FS

**Figure 2:**
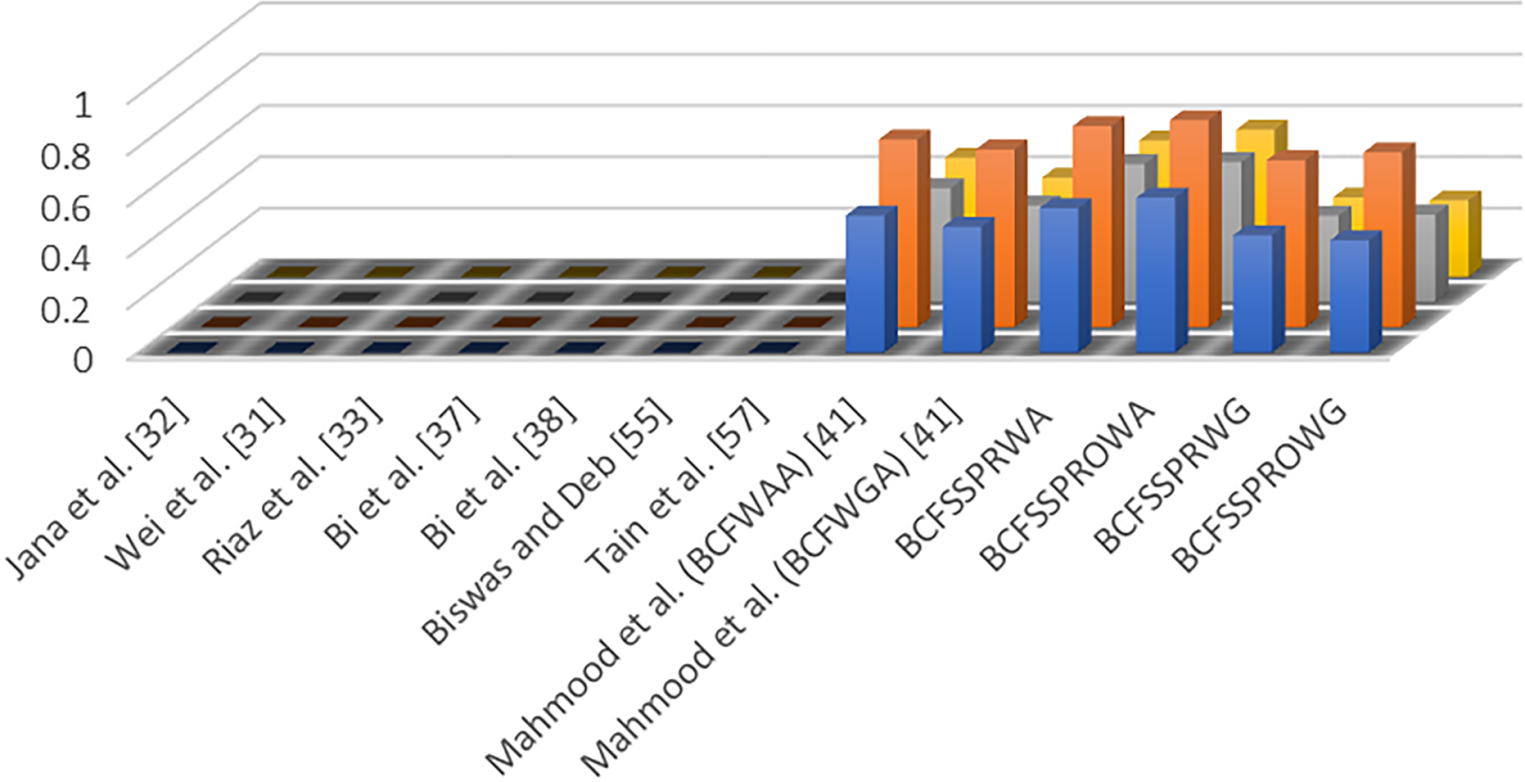
The graphical interpretation of the comparison.

As the deduced operators are in the model of the BCF set so let us assume the information of Table 1 which is in the model of BCF information and apply the deduced and prevailing operators to the information of Table 1. The overall results after utilizing deduced and current operators are established in Table 7 and Table 8 and a graphical presentation is exhibited in Fig. 2.

**Table 7:** The score argument of the invented and current work.

Source	${\mathfrak{S}}_{\mathfrak{B}} \left( {\Lambda }_{1} \right) $	${\mathfrak{S}}_{\mathfrak{B}} \left( {\Lambda }_{2} \right) $	${\mathfrak{S}}_{\mathfrak{B}} \left( {\Lambda }_{3} \right) $	${\mathfrak{S}}_{\mathfrak{B}} \left( {\Lambda }_{4} \right) $
Jana, Pal & Wang (2019)	Give out	Give out	Give out	Give out
Wei et al. (2018)	Give out	Give out	Give out	Give out
Riaz et al. (2022)	Give out	Give out	Give out	Give out
Bi et al. (2019)	Give out	Give out	Give out	Give out
Bi, Dai & Hu (2018)	Give out	Give out	Give out	Give out
Biswas & Deb (2021)	Give out	Give out	Give out	Give out
Tian et al. (2022)	Give out	Give out	Give out	Give out
Liu, Khan & Mahmood (2019)	Give out	Give out	Give out	Give out
Mahmood et al. (2022b) (BCFWAA)	0.533	0.732	0.444	0.465
Mahmood et al. (2022b) (BCFWGA)	0.489	0.693	0.376	0.387
Originated operator (BCFSSPRWA)	0.562	0.784	0.538	0.532
Originated operator (BCFSSPROWA)	0.603	0.807	0.546	0.574
Originated operator (BCFSSPRWG)	0.457	0.651	0.336	0.31
Originated operator (BCFSSPROWG)	0.473	0.682	0.341	0.298

**Table 8:** The ranking among the invented and current work.

Source	Ranking
Jana, Pal & Wang (2019)	Give out
Wei et al. (2018)	Give out
Riaz et al. (2022)	Give out
Bi et al. (2019)	Give out
Bi, Dai & Hu (2018)	Give out
Biswas & Deb (2021)	Give out
Tian et al. (2022)	Give out
Liu, Khan & Mahmood (2019)	Give out
Mahmood et al. (2022b) (BCFWAA)	Λ_2_ > Λ_1_ > Λ_4_ > Λ_3_
Mahmood et al. (2022b) (BCFWGA)	Λ_2_ > Λ_1_ > Λ_4_ > Λ_3_
Originated operator (BCFSSPRWA)	Λ_2_ > Λ_1_ > Λ_3_ > Λ_4_
Originated operator (BCFSSPROWA)	Λ_2_ > Λ_1_ > Λ_4_ > Λ_3_
Originated operator (BCFSSPRWG)	Λ_2_ > Λ_1_ > Λ_3_ > Λ_4_
Originated operator (BCFSSPROWG)	Λ_2_ > Λ_1_ > Λ_3_ > Λ_4_

The prevailing AOs such as Dombi introduced by Jana, Pal & Wang (2019), Hamacher introduced by Wei et al. (2018), sine trigonometric established by Riaz et al. (2022) under BF information can cope with BF information and fuzzy information but can’t overcome the information containing extra fuzzy information that is second dimension because the unreal parts in both positive truth grade and negative truth grade are missing in the model of BFS. However, the introduced AOs can cope with bipolar fuzzy information just by overlooking the unreal parts in both positive and negative truth grades. Thus, the devised AOs can be reduced to the structure of BFS. Similarly, the arithmetic and geometric AOs introduced by Bi et al. (2019) and Bi, Dai & Hu (2018) respectively under CF information can cope with BF information and fuzzy information but can’t overcome the information containing counter property that is a negative aspect of the object because the negative truth grade is missing in the structure of CFS. However, the devised AOs can cope with complex fuzzy information just by ignoring the negative truth grade. Consequently, the introduced AOs can be reduced to the model of CFS. That is why the AOs under BFS and CFS failed to cope with the information in Table 1. Further, through investigated operators, DM approach, and AOs established by Mahmood et al. (2022b), the required result has been achieved which is given in Table 5, and based on the achieved outcomes the ranking is displayed in Table 6. According to the achieved outcomes and ranking presented through investigation and the prevailing operators deduced by Mahmood et al. (2022b), Λ_2_ is the most prevalent mental disorder in the existing alternatives. It is now obvious from the above discussion that the introduced work is more generalized than the existing work in the environment of FS, BFS, and CFS. Moreover, the parameter variable ℷ < 0 made the introduced operator more flexible and handy than the existing operators. The introduced operator can be reduced to the operators introduced by Mahmood et al. (2022b). This implies that the introduced work is more superior and effective than the mentioned works.

While the rest of the operators that are SS power AOs (Biswas & Deb, 2021) in the structure of Pythagorean fuzzy information, SS prioritized AOs (Tian et al., 2022; Liu, Khan & Mahmood, 2019) in the model of picture fuzzy information and neutrosophic fuzzy information failed to solve the established data as the operators are not in the model of BCF information even the AOs based on the SS t-norm and t-conorms. This implies that the existing AOs based on the SS t-norm and t-conorm can’t cope with the BCF information.

## Conclusion

The primary goals of this article were as follows:

1. We introduced SS operational laws based on the BCFN.
2. We introduced BCFSSPRWA, BCFSSPROWA, BCFSSPRWG, and BCFSSPROWG operators based on the interpreted SS operational laws for BCFNs.
3. We devised a DM approach based on the SS AOs in the environment of BCF information.
4. We analyzed the mental disorder and their types with the assistance of the approach of DM based on the SS AOs in the environment of BCF information.
5. Then, we prioritized the types of mental disorders by taking artificial data and utilizing the introduced approach of DM and determined that Λ_2_ is the most prevalent mental disorder of the considered 4 kinds of mental disorders.
6. The introduced operator was compared with certain current operators for revealing the priority and superiority of the introduced work.

Furthermore, in the section of comparative study, we noticed that the introduced operator can cope with various other information that as fuzzy information, BF information, and CF information, and the introduced operators can be converted into the structure of FS, BFS, and CFS. The parameter variable ℷ < 0 made the introduced operator more flexible and handy than the existing operators. The concocted work can be utilized in various areas such as DM, artificial intelligence, computer sciences, *etc*.

In the future, we hope to examine the concept of bipolar complex intuitionistic FS (Al-Husban, 2022; Jan et al., 2022), bipolar complex intuitionistic fuzzy graph (Nandhinii & Amsaveni, 2022), and bipolar complex fuzzy soft set (Mahmood et al., 2022c) and expand our work on these conceptions. Moreover, we hope to investigate novel notions such as rough sets, soft matrices vague sets, *etc.*, and try to relate the introduced work with these notions.

The full and short form of important words are listed in Table 9.

**Table 9:** The full form and short form of important keywords.

Short form	Full form
FS	Fuzzy set
BFS	Bipolar fuzzy set
CFS	Complex fuzzy set
BCFS	Bipolar complex fuzzy set
BCF	Bipolar complex fuzzy
BCFN	Bipolar complex fuzzy number
SS	Schweizer-Sklar
AO	Aggregation operators
BCFSSPRWA	Bipolar complex fuzzy Schweizer-Sklar prioritized weighted averaging
BCFSSPROWA	Bipolar complex fuzzy Schweizer-Sklar prioritized ordered weighted averaging
BCFSSPRWG	Bipolar complex fuzzy Schweizer-Sklar prioritized weighted geometric
BCFSSPROWG	Bipolar complex fuzzy Schweizer-Sklar prioritized ordered weighted geometric
DM	Decision Making

##  Supplemental Information

10.7717/peerj-cs.1434/supp-1 Supplemental Information 1 Raw data

10.7717/peerj-cs.1434/supp-2 Supplemental Information 2 Formulas used for calculation

## Additional Information and Declarations

### Data Availability

The following information was supplied regarding data availability: The data is available in the Supplementary File.
