# Supplementary material for: Evaluation of mental disorder with prioritization of its type by utilizing the bipolar complex fuzzy decision-making approach based on Schweizer-Sklar prioritized aggregation operators
Source: PeerJ Comput Sci. 2023 Nov 7;9:e1434. doi: 10.7717/peerj-cs.1434 (PMC13011981; doi:10.7717/peerj-cs.1434)
Supplement: Supplemental Information 1 [file peerj-cs-09-1434-s001.docx]

|  | $\mathfrak{C}_{\mathfrak{At-}\boldsymbol{1}}$ | $\mathfrak{C}_{\mathfrak{At-}\boldsymbol{2}}$ | $\mathfrak{C}_{\mathfrak{At-}\boldsymbol{3}}$ | $\mathfrak{C}_{\mathfrak{At-}\boldsymbol{4}}$ |
| --- | --- | --- | --- | --- |
| $\boldsymbol{\Xi}_{\mathfrak{Ap-}\boldsymbol{1}}$ | $\left( \begin{aligned} 0.451+\iota0.56, \\ -0. 75-\iota0.37 \end{aligned} \right)$ | $\left( \begin{aligned} 0.632+\iota0.432, \\ -0. 474-\iota0.456 \end{aligned} \right)$ | $\left( \begin{aligned} 0.465+\iota0.398, \\ -0. 781-\iota0.298 \end{aligned} \right)$ | $\left( \begin{aligned} 0.576+\iota0.571, \\ -0. 198-\iota0.61 \end{aligned} \right)$ |
| $\boldsymbol{\Xi}_{\mathfrak{Ap-}\boldsymbol{2}}$ | $\left( \begin{aligned} 0.871+\iota0.76, \\ -0. 55-\iota0.26 \end{aligned} \right)$ | $\left( \begin{aligned} 0.712+\iota0.542, \\ -0. 363-\iota0.345 \end{aligned} \right)$ | $\left( \begin{aligned} 0.576+\iota0.487, \\ -0. 26-\iota0.14 \end{aligned} \right)$ | $\left( \begin{aligned} 0.91+\iota0.642, \\ -0. 115-\iota0.2 \end{aligned} \right)$ |
| $\boldsymbol{\Xi}_{\mathfrak{Ap-}\boldsymbol{3}}$ | $\left( \begin{aligned} 0.612+\iota0.617, \\ -0. 551-\iota0.361 \end{aligned} \right)$ | $\left( \begin{aligned} 0.523+\iota0.353, \\ -0. 585-\iota0.567 \end{aligned} \right)$ | $\left( \begin{aligned} 0.365+\iota0.29, \\ -0. 951-\iota0.471 \end{aligned} \right)$ | $\left( \begin{aligned} 0.479+\iota0.713, \\ -0. 852-\iota0.876 \end{aligned} \right)$ |
| $\boldsymbol{\Xi}_{\mathfrak{Ap-}\boldsymbol{4}}$ | $\left( \begin{aligned} 0.761+\iota0.23, \\ -0. 393-\iota0.76 \end{aligned} \right)$ | $\left( \begin{aligned} 0.434+\iota0.564, \\ -0.696 -\iota0. 678 \end{aligned} \right)$ | $\left( \begin{aligned} 0.713+\iota0.651, \\ -0. 92-\iota0.81 \end{aligned} \right)$ | $\left( \begin{aligned} 0.371+\iota0.371, \\ -0.687 -\iota0.27 \end{aligned} \right)$ |
